# Mating ecology explains patterns of genome elimination

**DOI:** 10.1111/ele.12383

**Published:** 2014-10-17

**Authors:** Andy Gardner, Laura Ross

**Affiliations:** 1 School of Biology, University of St Andrews, Dyers BraeSt Andrews, KY16 9TH, UK; 2 Institute of Evolutionary Biology, University of EdinburghKing's Buildings, Edinburgh, EH9 3JT, UK

**Keywords:** Extinction, genomic imprinting, haplodiploidy, inbreeding, meiotic drive, paternal genome elimination, paternal genome loss, sex determination, sex ratio, sib-mating

## Abstract

Genome elimination – whereby an individual discards chromosomes inherited from one parent, and transmits only those inherited from the other parent – is found across thousands of animal species. It is more common in association with inbreeding, under male heterogamety, in males, and in the form of paternal genome elimination. However, the reasons for this broad pattern remain unclear. We develop a mathematical model to determine how degree of inbreeding, sex determination, genomic location, pattern of gene expression and parental origin of the eliminated genome interact to determine the fate of genome-elimination alleles. We find that: inbreeding promotes paternal genome elimination in the heterogametic sex; this may incur population extinction under female heterogamety, owing to eradication of males; and extinction is averted under male heterogamety, owing to countervailing sex-ratio selection. Thus, we explain the observed pattern of genome elimination. Our results highlight the interaction between mating system, sex-ratio selection and intragenomic conflict.

## Introduction

**Figure 1:**
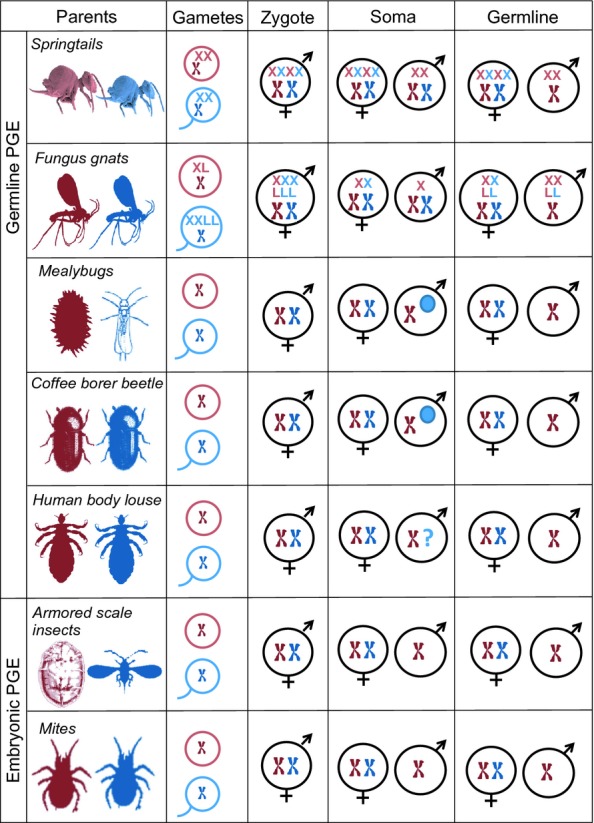
Parent-of-origin-specific genome elimination (GE), whereby an individual discards the chromosomes inherited from one parent, and transmits only those inherited from the other parent. We include only those cases where GE is sex-limited. Rows represent independent origins of GE. Column 1: adult generation. Column 2: gametes produced. Column 3: embryos shortly after fertilisation. Column 4: offspring soma. Column 5: offspring germline. Blue: male. Red: female. X: presence of X-chromosome (colour indicates parental origin). L in fungus-gnat entry: a germline-linked chromosome. Blue circle in mealybugs and coffee-borer-beetles entries: complete heterochromatisation of paternal genome. ‘?’ in body-louse entry: lack of information about somatic effects. ‘Germline PGE’: eliminated genome retained throughout development but not transmitted to offspring. ‘Embryonic PGE’: eliminated genome lost early in development, resulting in haploidy.

Under standard mendelian inheritance, individuals receive one set of chromosomes from each of their parents, and transmit one set of chromosomes to each of their offspring, without bias according to each chromosome's parent of origin. However, across thousands of animal species, some individuals (typically members of one sex) systematically transmit only those chromosomes that they inherited from a particular parent (Table 1 and Fig. 1; Burt & Trivers 2006). For example, in the citrus mealybug *Planococcus citri*, a male's sperm carry only the chromosomes he inherited from his mother, all paternal chromosomes having been eliminated, whereas a female's oocytes carry chromosomes from both of her parents. This ‘genome elimination’ (GE) is a whole-genome form of meiotic drive and, accordingly, its evolutionary rationale makes sense from a selfish-gene perspective: a gene that ensures it is passed on to all – as opposed to only half – of an individual's offspring enjoys a two-fold selective advantage, and may increase in frequency unless the number of surviving offspring is more than halved as a consequence (Bull 1979). Indeed, the real evolutionary puzzle is to explain why GE occurs only in some species and not in others (Normark 2004).

**Table 1:** Overview of all taxonomic groups with PGE

Class	Order	Clade	Number of species	Type of PGE	Male soma	Sex chromosomes	Ancestral SD	Sib-mating/Inbreeding^*^	References
Acari	Mesostigmata	Phytoseiidae, Otopheidomenidae, Ascoidea	2000 (10)	Embryonic PGE	Haploid (genome loss)	No sex chr.	XX/XY or XX/X0	Strong evidence (WM, WF, SB, SM, PS)	(Nelson-Rees *et al.* 1980; Norton *et al.* 1993; Nagelkerke & Sabelis 1998; Burt & Trivers 2006)
Collembola	Symphypleona	Symphypleona	1180 (6)	Germline PGE	Diploid with X elimination	X1X2X1X2/X1X200	XX/X0	Some evidence (WM, WF, PG, SB)	(Dallai *et al.* 2001; Burt & Trivers 2006)
Insecta	Coleoptera	Cryphalini	190 (1)	Germline PGE	Diploid with paternal genome silencing	No sex chr.	XX/XY	Strong evidence (WM, SB, SM, PG)	(Brun *et al.* 1995; Borsa & Kjellberg 1996; Gauthier 2010)
Insecta	Diptera	Cecidomyiidae	6168 (22)	Germline PGE	Diploid with X elimination	XX/X0	XX/XY	No evidence	(Painter 1966; Stuart & Hatchett 1991; Burt & Trivers 2006; Benatti *et al.* 2010)
Insecta	Diptera	Sciaridae	2300 (10)	Germline PGE	Diploid with X elimination	XX/X0	XX/XY	No evidence	(Metz 1938; Crouse 1960; Haig 1993a, b; Goday & Esteban 2001; Sánchez 2010)
Insecta	Hemiptera	Neococcoidea	4000 (270)	Germline PGE	Diploid with paternal genome silencing	No sex chr.	XX/X0	Some evidence (WF, PS, PG)	(Brown & Nur 1964; Nur 1990; Gavrilov 2007; Ross *et al.* 2010a; Martins *et al.* 2012)
Insecta^$^	Hemiptera	Diaspididae	2650 (139)	Embryonic PGE	Haploid (genome loss)	No sex chr.	XX/X0	Some evidence (WF, PS)	(Brown & Nur 1964; Nur 1990)
Insecta	Phthiraptera	*Pediculus humanus humanus*	? (1)	Germline PGE	Unknown	No sex chr.	XX/XY?	Some evidence (PS, PG, SB, WM, WF)	(Perotti *et al.* 2004; McMeniman & Barker 2005; Ascunce *et al.* 2013)

Rows represent independent origins of PGE, except for Diaspididae (indicated by^$^), which represents change in type of PGE (germline → embryonic). Number of species column: estimate assumes PGE is conserved across whole clade; number of species for which there is direct evidence of PGE given in parentheses. Types of PGE: ‘embryonic’ when complete paternal genome is eliminated from both soma and germline in early embryogenesis; ‘germline’ when paternal genome only completely eliminated in the germline during spermatogenesis. Male soma and sex-chromosome information based on karyotype analysis of species with PGE. Ancestral-SD system inferred from karyotype data for diploid sister groups. Inbreeding inferred from *F*_IS_, mating system and sex-ratio data (WM, wingless males; WF, wingless females; SB, female-biased sex ratios; PG, population-genetic evidence of low genetic diversity and excess homozygosity; PS, life history leading to strong meta-population structure; SM, frequent sib-mating).

There is a clear pattern in the incidence and type of GE that occurs in the animal kingdom: it is commonly found in association with inbreeding, under male heterogamety, in males, and in the form of paternal genome elimination (Table 1 and Fig. 1). This suggests that a species' mating ecology is an important factor in predisposing it to GE. A rich literature spanning a century of work in ecology, population genetics and cytology has yielded several hypotheses as to how inbreeding impacts upon the evolution of GE, both directly and in its interaction with a species' sex determination system (Table 2). However, these ideas lead to different – indeed, sometimes diametrically opposite – predictions, and the complexity of the problem means that a full, quantitative analysis is lacking (Bull 1983; Burt & Trivers 2006).

**Table 2:** An overview of adaptive hypotheses for the evolution of paternal genome elimination (PGE)

Hypothesis	Prediction	References	Notes
Meiotic drive I	Inbreeding inhibits GE, because drive is only worthwhile in heterozygotes	Brown (1964), Bull (1979)	This effect is captured in the present model.
Meiotic drive II	Inbreeding promotes GE, as eliminated genome is more likely to acquiesce to the driving genome's interests	Burt & Trivers (2006)	This effect is captured in the present model.
Local mate competition I	Inbreeding promotes GE, as it favours female bias, and GE may enable maternal control of sex allocation	Hamilton (1967), Borgia (1980), Normark (2004)	Although straightforward for evolution of male haploidy *per se*, the argument is obscure for GE *per se*. This effect is neglected in the present model.
Local mate competition II	Inbreeding promotes GE, as it favours female bias, and GE may lead to female bias in some scenarios	Bull (1979), Haig (1993a)	Bull (apparently incorrectly) attributed this to Hamilton & Borgia, and dismissed it as lacking generality. This effect is captured in the present model.
Maternally transmitted endosymbiont	Inbreeding promotes GE, because it favours female bias, brought about by GE induced by endosymbiont in order to enhance its own transmission	Normark (2004), Kuijper & Pen (2010)	Formal analysis by Kuijper & Pen considered the interests of endosymbiont and maternal genes only. Analysis only applies to embryonic, not to germline, GE. Endosymbionts are neglected in the present model.

Here, we develop a mathematical kin selection model to determine how degree of inbreeding, mode of sex determination, genomic location, pattern of gene expression and parental origin of the eliminated genome interact to determine the fate of GE alleles. We identify those scenarios under which GE may arise in the population by performing invasion analyses, and we identify those scenarios in which GE may be maintained in the population by performing equilibrium analyses and assessing the impact of GE upon population viability. Our aim is to assess the constraints that a species' mating ecology imposes upon its ability to evolve – and survive – GE, thereby explaining its pattern of incidence in the animal world. Although specifically focusing upon GE, our analysis yields general insights into how mating system and sex-ratio selection shape conflicts within and between individuals, with application to sex-chromosome meiotic drive, endosymbiotic parasitism and haplodiploidy.

## Materials and Methods

### Mathematical model

We assume a gonochoristic, diploid population, subdivided into a large number of mating groups, each containing a large number of individuals. We denote by *a* the probability that a female and a male, randomly chosen from the same mating group, share the same mother. We assume that each female mates with a large number of males, and at random within her mating group, such that the probability of mating partners sharing the same father is effectively zero. Thus, *a* represents the incidence of mating between maternal siblings, and varying this key ecological parameter allows us to explore the whole continuum from outbreeding (*a *=* *0) to chronic inbreeding (*a *=* *1). After mating, males die and mated females disperse to new patches, as in Hamilton's (1967) classic model of local mate competition (LMC).

**Figure 2:**
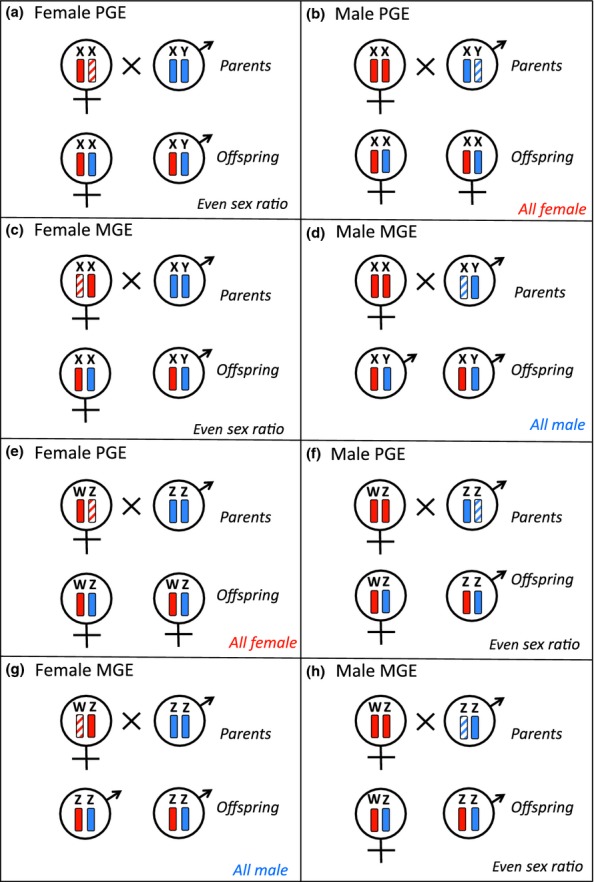
The consequences of genome elimination (GE) for offspring sex ratio. GE in the homogametic sex – that is, females under XX or XO sex determination (panels a & c) and males under ZW or ZO sex determination (panels f & h) – has no impact on offspring sex ratio. Paternal genome elimination (PGE) in the heterogametic sex – that is, males under XX or XO sex determination (panel b) and females under ZW or ZO sex determination (panel e) – leads to a female-biased offspring sex ratio. Maternal genome elimination (MGE) in the heterogametic sex – that is, males under XX or XO sex determination (panel d) and females under ZW or ZO sex determination (panel g) – leads to a male-biased offspring sex ratio.

We consider that GE alleles may be located on autosomes or sex chromosomes, and may induce maternal genome elimination (MGE) or paternal genome elimination (PGE). We allow for the genes to be active in females or males, to induce effects either in the carrier or the carrier's daughters or sons, and – for genes inducing GE in the carrier – to have different effects if the gene is inherited from the mother or the father (i.e. genomic imprinting; Haig 2002). We assume ‘germline’ GE, in which one parent's genome is excluded from the focal individual's gametes, but is present and active in the individual's somatic tissue, as is this is likely the ancestral form of GE (see Discussion). We assume that GE in females reduces their number of surviving offspring by a fraction α and that GE in males reduces their number of surviving offspring by a fraction β, as a consequence of upsetting normal chromosomal segregation. We assume that sex is determined by the individual's own genotype, and consider both male (XY and XO) and female (ZW and ZO) heterogamety. An important feature of the model is that GE in the heterogametic sex results in offspring sex ratio bias, towards males for MGE and towards females for PGE (Fig. 2). No sex-ratio bias obtains if GE is absent or restricted to the homogametic sex (Fig. 2).

### Invasion analysis

We analyse our model using the neighbour-modulated-fitness methodology of Taylor & Frank (1996; Supporting Information). This is a recipient-centred approach to kin selection, which considers the impact of social partners on a focal individual's fitness, and gives the same results as the actor-centred inclusive-fitness approach, which considers the impact of a focal individual on the fitness of her social partners (Hamilton 1964; Gardner *et al.* 2011). Here, the recipients are genes in potential zygotes, and they are separated into classes according to the sex of the individual, the sex of the parent of origin and the sex of the grandparent of origin. The condition for natural selection to favour an allele for GE is that instances of that allele are fitter (i.e. send more gene copies into future generations) than instances of alternative alleles in the population. That is, 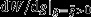, where *W* is expected relative fitness, *g* is genetic predisposition for GE, and 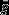 is the population frequency of GE (Taylor & Frank 1996). A zygote's expected fitness depends on its probability of conception and its probability of surviving viability selection (both of which depend on the action of GE in the zygote's parents) and expected mating success conditional upon surviving to adulthood (which may depend on the action of GE among those parents contributing offspring to the zygote's future mating group).

Invasion of GE occurs when the corresponding allele increases in frequency from rarity, that is 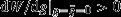. We reformulate this invasion condition to determine the potential for GE (Supporting Information). Specifically, for GE in females, the invasion condition may be expressed in the form α < γ, where α is the actual cost of GE, in terms of number of surviving offspring, and γ is the maximum cost that is tolerated with GE still able to invade, and is a function of model parameters. A positive potential for GE (γ > 0) indicates that costly GE may invade, a negative potential for GE (γ < 0) indicates that GE would need to provide a benefit, in terms of number of surviving offspring, in order for it to be able to invade, and zero potential for GE (γ = 0) indicates that GE cannot invade if it incurs any cost and will invade if it provides any benefit. The potential for GE may also be defined for males, by reformulating the invasion condition as β < γ. In addition to allowing comparisons and contrasts across different mating ecologies, the potential for GE provides a way of gauging a particular genic actor's interests with respect to GE and, hence, allows quantification of genetic conflicts of interest.

### Equilibrium analysis

Focusing attention upon scenarios in which GE invades, we next investigate its subsequent evolutionary fate (Supporting Information). The condition for increase in an allele's frequency is computed in the same way as in the invasion analysis. However, we now assume that GE incurs no viability cost (α = β = 0), mainly for simplicity and also because we consider that natural selection may fine-tune the mechanism to reduce such deleterious by-product effects (in reality, these are unlikely to be completely absent). Having established that GE can invade, we check for an intermediate equilibrium level by solving 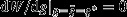. And we determine when GE may go to fixation according to the condition 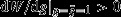. Straightforward fixation of GE in the heterogametic sex leads to the eradication of all individuals of one sex from the population (males for PGE, females for MGE; Fig. 2). Accordingly, we consider that fixation leads to population extinction (Hamilton 1967). Finally, for scenarios in which GE is maintained at an intermediate level, we consider its long-term fate following the establishment of a novel mechanism of sex determination, which eliminates any impact of GE upon the sex ratio (Supporting Information).

## Results

### Origin of genome elimination

**Figure 3:**
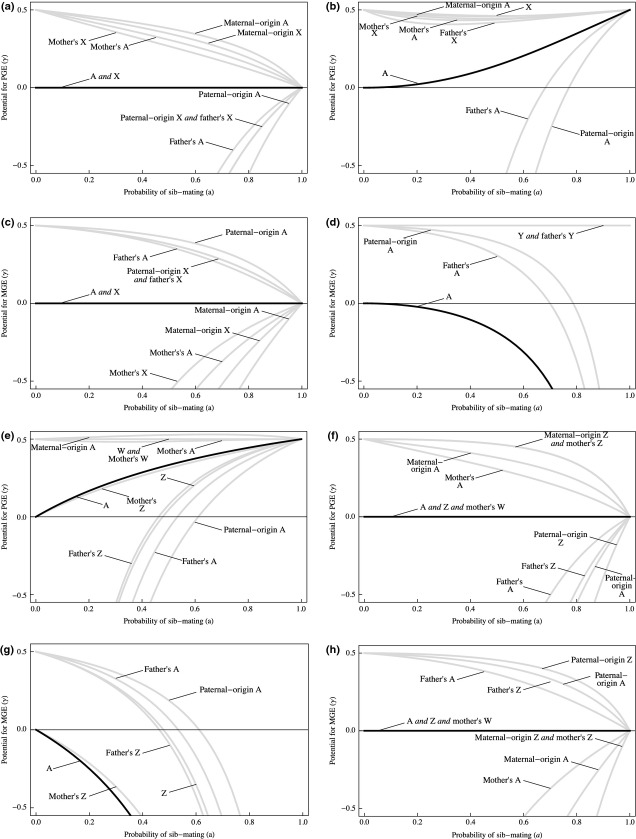
The origin of genome elimination (GE). Results of the invasion analyses, for: (a) Paternal genome elimination (PGE) in females under XY or XO sex determination; (b) PGE in males, XY/XO; (c) Maternal genome elimination (MGE) in females, XY/XO; (d) MGE in males, XY/XO; (e) PGE in females, ZW/ZO; (f) PGE in males, ZW/ZO; (g) MGE in females, ZW/ZO; (h) MGE in males, ZW/ZO. In each case, potential for GE is shown for each class of genic actor, for whole-range of sib-mating (0 ≤  *a *≤* *1). A: autosomal gene. X: X-linked gene. Y: Y-linked gene. Z: Z-linked gene. W: W-linked gene. Unimprinted autosomal genes (black lines) have positive potential for GE only under sib-mating (*a *>* *0), and for PGE in heterogametic males (panel b) or PGE in heterogametic females (panel e), and so these are the scenarios in which GE robustly invades.

We assess the impact of the degree of inbreeding, mode of sex determination, genomic location, pattern of gene expression and parental origin of the eliminated genome on the evolutionary invasion of GE alleles. For each scenario, we calculate the potential for GE (Fig. 3). This is mediated by two key effects: first, GE is a form of genetic drive, and as such it provides a two-fold direct transmission advantage for driving genes and a corresponding direct transmission disadvantage for those genes that are driven against; second, GE may lead to sex-ratio bias among an individual's offspring, and this may provide a selective advantage or disadvantage depending upon the direction of bias and the population's mating system.

#### Drive

The immediate consequences of drive are illustrated by considering PGE in females under XY sex determination (Fig. 3 panel a). The qualitative results for this permutation of the model readily generalise to all forms of GE in the homogametic sex, which has no impact on offspring sex ratio (PGE and MGE in females under XY/XO and in males under ZW/ZO; Fig. 3 panels a, c, f and h). Here, there are four types of genic actor. First, there are genes that directly benefit, on average, from drive: the female's maternal-origin autosomal and maternal-origin X-chromosomal genes, and her mother's autosomal and X-chromosomal genes. These genes have positive potential for PGE: in particular, γ = 0.5 under outbreeding (*a *=* *0), reflecting how the two-fold benefit of driving can offset even a halving of offspring number. Second, there are genes who suffer, on average, from drive: the female's paternal-origin autosomal and paternal-origin X-chromosomal genes, and her father's autosomal and X-chromosomal genes. These genes have negative potential for PGE: in particular, γ = −∞ under outbreeding (*a *=* *0), reflecting how no increase in offspring number can compensate for their elimination from the individual's gametes. Third, there are genes that are equally likely to directly benefit or suffer from drive: the female's unimprinted autosomal and X-chromosomal genes. These genes have zero potential for PGE, γ = 0, reflecting how they are favoured to prevent PGE if this reduces offspring number (α > 0) and to promote PGE if this increases offspring number (α < 0). Fourth, there are genes that are not directly affected by drive: the female's father's Y-chromosomal genes. Their potential for PGE is undefined, meaning that PGE is a neutral trait (West 2009).

A gene's inclusive fitness is not solely governed by its own replicative success but also by that of its homologues, to the extent that they share identity by descent (Gardner & Welch 2011). Accordingly, inbreeding (*a *>* *0) changes the above results quantitatively (but not qualitatively), such that a gene of the first type, who enjoys a direct benefit owing to drive, also suffers an indirect, kin-selected cost, owing to the disadvantage incurred by the identical-by-descent genes it drives against. Consequently, its potential for PGE falls from γ = 0.5 in the extreme of outbreeding (*a *=* *0) to γ = 0 in the extreme of chronic inbreeding (*a *=* *1). To the extent that PGE is controlled by such ‘maternal’ genes, sib-mating inhibits the evolution of PGE (Brown 1964; Bull 1979). Conversely, a gene of the second type, who suffers a direct cost owing to drive, also enjoys an indirect benefit, owing to the advantage accrued by its identical-by-descent homologues. Accordingly, its potential for PGE increases from γ = −∞ in the extreme of outbreeding to γ = 0 in the extreme of chronic inbreeding. To the extent that PGE is controlled by such ‘paternal’ genes, inbreeding promotes its evolution (Burt & Trivers 2006). Inbreeding has no effect on the potential for PGE for genes of the third and fourth types, who are unaffected by drive on average or at all.

To summarise, and to generalise to other instances of GE in the homogametic sex, there is extensive scope for genetic conflicts of interest over GE, diminishing as individuals are increasingly inbred, and no clear overall advantage or disadvantage of GE (Fig. 3 panels a, c, f and h). Definite predictions of the outcomes of such conflicts are impossible without specific information as to the additive genetic variance in GE contributed by each type of gene. However, a ‘parliament-of-genes’ approach (Leigh 1971; Supporting Information) suggests the outcome will be close to that favoured by the individual's unimprinted autosomal genes, owing to the necessarily close involvement of the individual's genome with the process of GE, the numerical superiority of autosomal genes (MacArthur 1961; Leigh 1971), and the cancelling-out of the interests of maternal-origin and paternal-origin imprinted genes (Haig 2002). Applying this pragmatic approach, GE appears relatively implausible in females under male heterogamety (Fig. 3 panels a and c) and in males under female heterogamety (Fig. 3 panels f and h). Accordingly, we discard these model permutations from further consideration.

#### Sex-ratio bias

In addition to the immediate effects of drive, GE may also accrue fitness effects owing to its consequences for offspring sex ratio. Sex-ratio bias arises from GE only when this occurs in the heterogametic sex (males under XY/XO, females under ZW/ZO; Fig. 3 panels b, d, e and g). In each case, PGE yields female bias, and MGE yields male bias (Fig. 2). As a consequence of LMC, sib-mating (*a *>* *0) may favour a relatively female-biased sex ratio (Hamilton 1967). This gives a selective advantage to PGE, and a selective disadvantage to MGE, in the heterogametic sex, relative to that obtained from consideration of the immediate effects of drive only (see above). This sex-ratio-selection effect need not be uniform across all genic actors, as the coefficient of inbreeding depends upon mode of inheritance, and parents may be in conflict with their offspring and each other over desired sex allocation (Hamilton 1967; Trivers 1974; Werren & Hatcher 2000). These details are captured by the model.

The resulting potential for GE is illustrated by considering it operating in heterogametic males. The potential for PGE is increased for almost all genic actors as a consequence of the LMC effect (Fig. 3 panel b). In particular, the male's unimprinted autosomal genes exhibit positive potential for PGE, rising from γ = 0 under the extreme of outbreeding (*a *=* *0) to γ = 0.5 under the extreme of chronic inbreeding (*a *=* *1). The exception is the Y-chromosomal genes that maintain a potential for PGE of γ = −∞ irrespective of the extent of sib-mating. This owes to our assumption of polyandry, which leads maternal-brothers to be unrelated through their fathers. Similarly, the potential for MGE is decreased for almost all genetic actors, because the resulting male bias is selectively disadvantageous under LMC (Fig. 3 panel d). Again, the exception is for the Y-chromosomal genes, which gain the usual two-fold drive benefit and hence exhibit a potential for MGE of γ = 0.5.

To summarise, and to generalise to other instances of GE in the heterogametic sex, there is extensive scope for genetic conflicts of interest, diminishing as populations are increasingly inbred, but with a robust overall advantage accruing to PGE, and a robust overall disadvantage accruing to MGE, under sib-mating, owing to the impact of GE on offspring sex ratio. Thus, MGE is relatively implausible in males under male heterogamety (Fig. 3 panel d) and in females under female heterogamety (Fig. 3 panel g), because it leads to a male-biased offspring sex ratio (Fig. 2d and g). Accordingly, we discard these model permutations from further consideration.

### Maintenance of genome elimination

**Figure 4:**
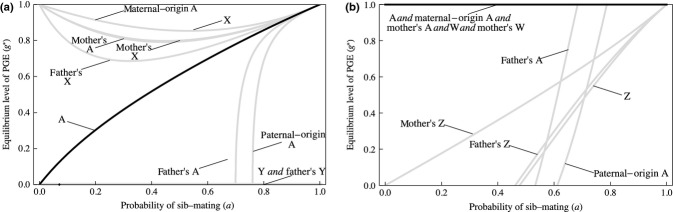
The maintenance of genome elimination (GE). Results of the evolutionary equilibrium analyses, for: (a) Paternal genome elimination (PGE) in heterogametic males (i.e. XY or XO sex determination); and (b) PGE in heterogametic females (i.e. ZW or ZO sex determination). In each case, the equilibrium level of PGE is shown for each class of genic actor, over the whole-range of sib-mating (0 ≤ *a *≤* *1). A indicates an autosomal gene, X indicates an X-linked gene, Y indicates a Y-linked gene, Z indicates a Z-linked gene and W indicates a W-linked gene. Unimprinted autosomal genes (black lines) favour an intermediate level of PGE under male heterogamety (panel a), owing to countervailing sex-ratio selection, and fixation of PGE – leading to population extinction – under female heterogamety (panel b), owing to a lack of countervailing sex-ratio selection, so PGE in heterogametic males is the only scenario in which the population robustly survives the evolution of GE.

We have narrowed the invasion of GE to two scenarios: PGE in males under male heterogamety (Fig. 3 panel b); and PGE in females under female heterogamety (Fig. 3 panel e). Moreover, we have identified inbreeding as an important driver of PGE in both instances. We now consider the scope for evolutionary maintenance of GE in each of these scenarios (Fig. 4).

#### PGE in heterogametic males

Although inbreeding facilitates the invasion of PGE in heterogametic males, this trait cannot generally increase to fixation. This is because, upon reaching a certain frequency, it will have brought the population sex ratio to its ‘optimal’ level, and further increases in the frequency of PGE are prevented by countervailing selection for reduced female bias acting directly on the PGE alleles (Burt & Trivers 2006). The equilibrium point depends upon the degree of inbreeding and the genomic location and mode of action of the genes underlying PGE (Fig. 4 panel a). Applying the parliament-of-genes approach, such that it is the interests of the unimprinted autosomal genes that are expected to win out, the equilibrium level of PGE increases approximately linearly, from 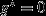 under outbreeding (*a *=* *0) to 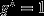 under the chronic inbreeding (*a *=* *1). That is, an intermediate equilibrium obtains for all intermediate degrees of inbreeding (0 < *a *<* *1).

In the longer term, it is useful to consider how this PGE may subsequently evolve should a new mode of sex determination, that eliminates the sex-ratio effects of GE, arise (Haig 1993a, b). We find that the ‘maternal’ genic actors, who derive a direct benefit from the immediate effect of drive, are favoured to increase PGE to fixation. In the event that they achieve this outcome, population viability is maintained because, under the new mode of sex determination, the fixation of PGE does not eradicate males. The ‘paternal’ genic actors are, conversely, favoured to completely suppress PGE. From the perspective of the individual's unimprinted autosomal genes, PGE is an entirely neutral trait. Accordingly, the parliament of genes is likely to be swayed by whichever extremist faction happens to have most power, and the long-term evolutionary fate of PGE therefore depends on the details of whether control lies mostly with the ‘maternal’ genes or with the ‘paternal’ genes.

#### PGE in heterogametic females

Although countervailing selection for reduced female bias prevents fixation of PGE in heterogametic males, this need not happen for PGE in heterogametic females (Fig. 4 panel b). Indeed, if – following the parliament-of-genes logic that control of PGE lies with the female's unimprinted autosomal genes – then our model predicts fixation of PGE in heterogametic females. This leads to the complete eradication of males, and hence population extinction. The absence of countervailing sex-ratio selection owes to PGE in heterogametic females leading to males having reduced reproductive value (Supporting Information). Indeed, in the limit of complete PGE in females, males have zero reproductive value, and all reproductive value belongs to females' maternal-origin genes. Hamilton (1967) provides more discussion of population extinction caused by sex-ratio distorters.

## Discussion

The two-fold transmission advantage enjoyed by GE alleles has raised the problem of why such biased inheritance is observed only in some species, and in some forms, but not others. In particular, it typically occurs in association with inbreeding, under male heterogamety, in males and in the form of PGE. Our analysis has clarified that, whilst some genes do gain a transmission advantage from drive, others suffer a disadvantage from reduced transmission, and ecological factors are important in providing a robust advantage for GE. In particular, we find that: (1) inbreeding promotes the elimination of the paternal genome in the heterogametic sex (i.e. males in XY/XO systems and females in ZW/ZO systems), (2) this may lead to population extinction under female heterogamety (i.e. ZW/ZO systems), owing to the eradication of males and (c) extinction is averted under male heterogamety (i.e. XY/XO systems), owing to countervailing sex-ratio selection. That is, a species' mating ecology imposes constraints upon its predisposition to evolve and survive GE, and this explains the widely observed pattern of PGE in heterogametic males under inbreeding.

### Intragenomic conflict over genome elimination

Our analysis has considered genetic conflicts of interest within the nuclear family and within the individual's own genome. Separate consideration of the individual's maternal-origin genes vs. the genes carried by the individual's mother reveals that these distinct sets of genes have distinct evolutionary interests, as do the individual's paternal-origin genes vs. the genes carried by the individual's father. For example, genes residing on a maternal-origin autosome enjoy the full transmission benefit of PGE whereas genes residing on one of the mother's autosomes enjoy a smaller benefit as they are not guaranteed to have been passed on to the individual who exhibits the PGE phenotype. This clarifies that intragenomic imprinting conflicts are conceptually distinct from (though may arise in similar contexts to) conflicts of interest between parents, despite these two phenomena having often been conflated (as discussed by Haig 2002).

Within male-heterogametic systems, PGE appears to be more common under XO than XY inheritance, as no PGE species with recognisable sex chromosomes has a Y chromosome (Table 1). Burt & Trivers (2006) suggested that this owes to the Y chromosome being strongly opposed to PGE in males, and inhibiting its evolution. In support of this suggestion, we find PGE is never favoured by Y-chromosomal genes, which suffer complete transmission failure under PGE, and this drive disadvantage cannot be offset by any offspring-survival or sex-ratio benefit, for any degree of inbreeding. Relaxing our assumption of polyandry could change model predictions in this respect, as this would lead to relatedness between mate competitors with respect to their Y-chromosomal genes, enhancing selection for female bias (cf Hamilton 1967), but the qualitative conclusion that the Y chromosome is less inclined to PGE than are the autosomes appears to be robust. However, in many species the Y chromosome is relatively degenerate, containing few active genes, and hence may have insufficient power to inhibit PGE. Accordingly, loss of the Y chromosome more likely occurred subsequently to the transition to PGE. Indeed, in most cases, the diploid sister groups of PGE taxa have XY sex determination (Table 1).

### Mating system and genome elimination

Our model of sib-mating is motivated by the empirical observation that GE often coincides with a life history that leads to high levels of inbreeding (Table 1). Our model shows that sib-mating and resulting selection for female-biased sex ratios can facilitate the evolution of GE, and we suggest that this explains the apparent association. However, sib-mating need not be essential for the evolution of GE. Other forms of inbreeding, such as those arising from population viscosity, may also favour female-biased sex ratios (Gardner *et al.* 2009). Moreover, some dipteran species with PGE lack inbreeding altogether (Burt & Trivers 2006). It is also possible that GE drives the evolution of inbreeding, rather than the reverse, yielding an alternative explanation for their apparent association. For example, some species with PGE exhibit haploid gene expression in males (Table 1 and Fig. 1), which could promote inbreeding by purging recessive deleterious alleles and hence ameliorating the costs of homozygosity. However, inbreeding appears frequently among several PGE species that exhibit diploid-male gene expression (e.g. lice and springtails).

Our model makes a number of other assumptions about ancestral mating system. First, for analytical convenience, we have assumed extreme female promiscuity, though monandry may be more realistic for some species (Hamilton 1967). As GE in males may lead to a reduction in the number of viable sperm, lower female promiscuity could promote GE in males by reducing between-male sperm competition. Second, we have assumed a classic LMC scenario in which females mate at their natal patch prior to dispersal (Hamilton 1967), which is more realistic for some PGE species (lice, mites, coffee-borer beetles and springtails) than others (dipterans and scale insects). However, these details are not essential, and the major purpose of the model is to illustrate that any mating ecology that results in selection for sex-ratio bias may govern the evolution of GE.

### Germline vs. embryonic genome elimination

The defining feature of PGE in males is the absence of the paternal genome among the individual's gametes, and our model focuses upon this central feature. That is, it more closely captures the ‘germline PGE’ of those species in which elimination of the paternal genome occurs during spermatogenesis, rather than the ‘embryonic PGE’ of those species in which elimination occurs during early embryogenesis, which potentially involves additional fitness costs associated with somatically haploid males (Table 1 and Fig. 1). Such costs of haploidy mean that germline PGE is likely the ancestral form, and embryonic PGE the more derived form. Indeed, embryonic PGE has evolved from germline PGE at least in the scale insects (Nur 1990; Herrick & Seger 1999; Ross *et al.* 2010a).

Among those species with germline PGE, there are various somatic effects ranging from elimination of paternally derived sex chromosomes to complete transcriptional silencing of the paternal genome (Fig. 1 and Table 1). Our model provides two non-mutually exclusive explanations for this. First, once PGE has evolved, the paternally derived genes are under strong selection to evolve counteradaptations (Herrick & Seger 1999; Ross *et al.* 2010b). To avoid this, the maternal or maternally derived genes are selected to either: disable the paternal genome, leading to whole-genome heterochromatisation, as seen in scale insects and beetles; or else completely eliminate it, leading to embryonic PGE, as seen in mites and armoured scale insects. Second, fixation of PGE in males requires the evolution of a new sex-determining system that overrides genetic sex determination. For example, in species where sex is determined by X-dosage, the elimination of one X chromosome would convert a female (XX) into a male (X0), as occurs in springtails (Dallai *et al.* 2001) and Sciara flies (Haig 1993b). Indeed, sex-ratio selection might also favour the silencing or early elimination of the whole paternal genome, converting females (XX) to hemizygous males (X).

Our model explains the elimination of paternal – as opposed to maternal – chromosomes owing to its impact upon offspring sex ratio. Another reason why the paternal genome may be more vulnerable to elimination is anisogamy (Parker *et al.* 1972): an egg contains many more proteins and RNAs than does a sperm, so if elimination is under parental control, the mother's interests may be expected to dominate. This is likely to be important in the evolution of embryonic PGE, but less so in the evolution of germline PGE. Moreover, such imbalance in power fails to explain other patterns of GE, such as its confinement to males.

### Other forms of genome elimination

Our model has considered the most frequently occurring form of GE, whereby one haploid genome is eliminated according to its parent of origin. However, there are a number of reproductive systems that exhibit alternative forms of GE, typically involving hybridisation events where one species temporarily ‘borrows’ a genome from a related species, to express in its somatic tissues, but eliminates it from the germline (Beukeboom & Vrijenhoek 1998; Burt & Trivers 2006). In many, but not all, of these cases the hybridogenetic species is all-female. Our model shows that, in principle, this mating system could have evolved via PGE in heterogametic females, with extinction avoided despite the eradication of males because females were able to mate with males from a closely related species, but this is unlikely to be of empirical importance. First, if this were how hybridogenesis typically evolved, we would expect an overrepresentation of ancestral female heterogamety among these taxa, and although ZW is present in the ancestors of two hybridogenetic teleost fish, the ancestors of hybridogenetic frogs and stick insects were clearly XY (The Tree of Sex Consortium 2014). Second, in all cases, hybridogenesis apparently arose from an ancient hybridisation event, of which GE was a consequence rather than a cause (Burt & Trivers 2006).

However, there is one other case where genome elimination is dependent upon parent of origin. Androgenesis – found in two species of ant, four species of clam and one species of cypress tree – involves only sperm-contributing genes to zygotes, and maternal genomes being entirely eliminated (Beukeboom & Vrijenhoek 1998; Burt & Trivers 2006). In ants, it evolved from an already-asymmetric inheritance system (haplodiploidy) and resulted from selection pressures particular to eusociality. In clams and cypress, it evolved from hermaphroditism and occurs in every offspring (unlike PGE, which is restricted to males; Burt & Trivers 2006), so it resembles a modified form of parthenogenesis (with all reproductive value belonging to males rather than females) and may be better captured by classic models for the evolution of parthenogenesis.

Finally, PGE shares several key features with haplodiploidy (Hartl & Brown 1970; Bull 1979; Burt & Trivers 2006). In particular, all genes transmitted by males derive from their mothers, and are passed on only by their daughters. PGE and haplodiploidy often co-occur in closely related taxonomic groups, including scale insects, mites and beetles, which suggest that similar selection pressures underlie the evolution of both genetic systems. In addition to the drive benefits that are enjoyed by maternal-origin genes, the offspring sex ratio bias effect captured in our model may also apply to the evolution of haplodiploidy, as mating with a haploid male results in female bias that may be favoured under sib-mating (Hamilton 1967; Borgia 1980; Bull 1983). Sib-mating may also promote the evolution of haplodiploidy in other ways, unrelated to the effects considered in the present model, for example by purging recessive deleterious alleles and hence raising the viability of haploid males (Brown 1964). However, an alternative explanation for this empirical association is that haplodiploidy may evolve via PGE (Schrader & Hughes-Schrader 1931). This possibility remains to be formally explored, and presents an exciting avenue for future theoretical and empirical study.
