# Supplementary material for: Mating ecology explains patterns of genome elimination
Source: Ecol Lett. 2014 Oct 17;17(12):1602–12. doi: 10.1111/ele.12383 (PMC4240462; doi:10.1111/ele.12383)
Supplement: Supplementary file 1 — Supplementary [file ele0017-1602-SD1.pdf]

Gardner, A. & Ross, L. "Mating ecology explains patterns of genome elimination" Supplementary Material 1 – Extended Methods.

1. PGE in females and/or males under XY/XO inheritance: invasion analysis

1.1. Autosomal genes

Classes – There are 8 classes of individual, classified according to their sex $s \in \{f,m\}$, the grandparent of origin of their maternal-origin gene $T \in \{M,P\}$ and the grandparent of origin of their paternal-origin gene $U \in \{M,P\}$. Each class is notated in the form sTU, i.e. fMM, fMP, fPM, fPP, mMM, mMP, mPM and mPP.

Survival – Picking a zygote at random, it has some probability of belonging to each of the 8 classes, depending on whether its parents exhibited genome elimination. We model this as if there were 8 potential zygotes, of which one is chosen to come into existence. Moreover, the realised zygote may not survive to maturity, owing to viability costs associated with the genome elimination behaviour of its parents. Denoting the probability that the zygote's mother exhibited PGE by ϕ, the probability that the zygote's father exhibited PGE by μ, the viability cost associated with female PGE by α and the viability cost associated with male PGE by β, the probability that each potential zygote survives to maturity is: $S_{fMM}(\phi,\mu,\alpha,\beta) = (1/8)(1-\phi)(1-\mu) + (1/4)\phi(1-\mu)(1-\alpha) + (1/2)(1-\phi)\mu(1-\beta) + \phi\,\mu\,(1-\alpha)(1-\beta)$; $S_{fMP}(\phi,\mu,\alpha,\beta) = (1/8)(1-\phi)(1-\mu) + (1/4)\phi(1-\mu)(1-\alpha)$; $S_{fPM}(\phi,\mu,\alpha,\beta) = (1/8)(1-\phi)(1-\mu) + (1/2)(1-\phi)\mu(1-\beta)$; $S_{fPP}(\phi,\mu,\alpha,\beta) = (1/8)(1-\phi)(1-\mu)$; $S_{mMM}(\phi,\mu,\alpha,\beta) = (1/8)(1-\phi)(1-\mu) + (1/4)\phi(1-\mu)(1-\alpha)$; $S_{mMP}(\phi,\mu,\alpha,\beta) = (1/8)(1-\phi)(1-\mu) + (1/4)\phi(1-\mu)(1-\alpha)$; $S_{mPM}(\phi,\mu,\alpha,\beta) = (1/8)(1-\phi)(1-\mu)$; and $S_{mPP}(\phi,\mu,\alpha,\beta) = (1/8)(1-\phi)(1-\mu)$.

Reproductive success – Upon survival to maturity, every female achieves a unit of expected reproductive success, and every male achieves $(1-z(\phi',\mu',\alpha,\beta))/z(\phi',\mu',\alpha,\beta)$ units of expected reproductive success, where: $z(\phi',\mu',\alpha,\beta) = (\sum_{T\in\{M,P\},U\in\{M,P\}} S_{mTU}(\phi',\mu',\alpha,\beta)) / (\sum_{s\in\{f,m\},T\in\{M,P\},U\in\{M,P\}} S_{sTU}(\phi',\mu',\alpha,\beta))$ is the sex ratio (proportion male) in his mating group; ϕ' is the level of PGE among the females contributing offspring to the mating group; and μ' is the level of PGE among the fathers contributing offspring to the mating group. Thus, contingent upon survival, expected reproductive success is $R_{fTU}(\phi',\mu',\alpha,\beta) = 1$ for females and $R_{mTU}(\phi',\mu',\alpha,\beta) = (1-z(\phi',\mu',\alpha,\beta))/z(\phi',\mu',\alpha,\beta)$ for males.

Fitness – Expected fitness is given by the product of survival to maturity and expected reproductive success contingent upon surviving to maturity, i.e. $w_{sTU}(\phi,\phi',\mu,\mu',\alpha,\beta) = S_{sTU}(\phi,\mu,\alpha,\beta)\times R_{sTU}(\phi',\mu',\alpha,\beta)$. Because PGE is vanishingly rare in the population, the average fitness among all individuals of a particular class is given by $\bar{w}_{sTU}(\alpha,\beta) = w_{sTU}(0,0,0,0,\alpha,\beta)$, and each individual's fitness can be expressed relative to the average of their class, by $W_{sTU}(\phi,\phi',\mu,\mu',\alpha,\beta) = w_{sTU}(\phi,\phi',\mu,\mu',\alpha,\beta)/\ \bar{w}_{sTU}(\alpha,\beta)$.

Gene fitness –There are 16 classes of gene, because every class of individual carries one maternal-origin gene and one paternal-origin gene. Thus, gene classes are denoted in the form sTUv, where v $\in$ {1,2} according to whether the gene is of maternal (v = 1) or paternal (v = 2) origin. The fitness of a gene, defined in terms of its probability of being transmitted to a potential (rather than a realised) zygote, is proportional to the fitness of the individual who carries it. Consequently, the relative fitness of a gene is equal to the relative fitness of its carrier: $W_{\mathrm{sTU1}}(\phi,\phi',\mu,\mu',\alpha,\beta) = W_{\mathrm{sTU2}}(\phi,\phi',\mu,\mu',\alpha,\beta) = W_{\mathrm{sTU}}(\phi,\phi',\mu,\mu',\alpha,\beta)$.

Reproductive value – The flow of genes between classes determines each class's reproductive value. Each gene's class in any generation may provide some information and some uncertainty about that gene's origin in the previous generation. For example, genes of class fMM1 in the present generation are derived from genes of class fTU1 in the previous generation, where T = M or P with equal probability and U = M or P with equal probability. Consequently, the fMM1 gene derived from a fMM1 gene with probability $\wp_{\mathrm{fMM1}\leftarrow\mathrm{fMM1}}$ = ¼, from a fMP1 gene with probability $\wp_{\mathrm{fMM1}\leftarrow\mathrm{fMP1}}$ = ¼, from a fPM1 gene with probability $\wp_{\mathrm{fMM1}\leftarrow\mathrm{fPM1}}$ = ¼ and from a fPP1 gene with probability $\wp_{\mathrm{fMM1}\leftarrow\mathrm{fPP1}}$ = ¼. The reproductive value of a given class is shared among all the classes who contribute genes to that class, in proportion to the contributions that they make: $c_{\mathrm{sTUv}} = \sum_{\mathrm{s^*}\in\{\mathrm{f,m}\},\mathrm{T^*}\in\{\mathrm{M,P}\},\mathrm{U^*}\in\{\mathrm{M,P}\},\mathrm{v^*}\in\{1,2\}} \wp_{\mathrm{s^*T^*U^*v^*}\leftarrow\mathrm{sTUv}}\, c_{\mathrm{s^*T^*U^*v^*}}$. This defines a system of 16 linear equations, which can be written as $\mathbf{c} = \mathbf{c}\bullet\mathbf{G}$, where $\mathbf{c} = \{c_{\mathrm{fMM1}},c_{\mathrm{fMM2}},c_{\mathrm{fMP1}},\ldots,c_{\mathrm{mPP2}}\}$ is the vector of class reproductive values and $\mathbf{G}$ is the gene-flow matrix. Solving (i.e. finding the left eigenvector of $\mathbf{G}$ corresponding to the eigenvalue 1) obtains c_{sTUv} = 1/16 for all gene classes.

PGE in females – Genes can be assigned genic values g according to their heritable tendency for any trait of interest. The condition for natural selection to favour an increase in this trait is $\mathrm{d}W/\mathrm{d}g > 0$, where $W = \sum_{\mathrm{s}\in\{\mathrm{f,m}\},\mathrm{T}\in\{\mathrm{M,P}\},\mathrm{U}\in\{\mathrm{M,P}\},\mathrm{v}\in\{1,2\}} c_{\mathrm{sTUv}}\, W_{\mathrm{sTUv}}$. Here, g is the genic value associated with an autosomal gene in a potential zygote. Thus, the condition for increase in female PGE is $\sum_{\mathrm{s}\in\{\mathrm{f,m}\},\mathrm{T}\in\{\mathrm{M,P}\},\mathrm{U}\in\{\mathrm{M,P}\},\mathrm{v}\in\{1,2\}} c_{\mathrm{sTUv}} (\mathrm{d}W_{\mathrm{sTUv}}/\mathrm{d}\phi) \times (\mathrm{d}\phi/\mathrm{d}G) \times (\mathrm{d}G/\mathrm{d}g_{\mathrm{sTUv}}) > 0$, where G is the average genic value among those genes at the same locus whose expression controls PGE in the focal individual's mother. Consequently: $\mathrm{d}\phi/\mathrm{d}G$ is an arbitrary mapping between genic value and phenotypic value, and can be set to 1; and $\mathrm{d}G/\mathrm{d}g_{\mathrm{sTUv}} = p_{\mathrm{sTUv}}$ is the consanguinity between the focal class-sTUv gene and the genes, residing at the same locus, who control PGE in the mother of the focal gene's carrier. The consanguinities will depend upon which class or classes of genes control PGE in females -- see the Consanguinity section below, and Table A1.1.1, for details.

PGE in males – Similarly, the condition for increase in male PGE is $\sum_{\mathrm{s}\in\{\mathrm{f,m}\},\mathrm{T}\in\{\mathrm{M,P}\},\mathrm{U}\in\{\mathrm{M,P}\},\mathrm{v}\in\{1,2\}} c_{\mathrm{sTUv}} ((\mathrm{d}W_{\mathrm{sTUv}}/\mathrm{d}\mu) \times (\mathrm{d}\mu/\mathrm{d}G) \times (\mathrm{d}G/\mathrm{d}g_{\mathrm{sTUv}}) + (\mathrm{d}W_{\mathrm{sTUv}}/\mathrm{d}\mu') \times (\mathrm{d}\mu'/\mathrm{d}G') \times (\mathrm{d}G'/\mathrm{d}g_{\mathrm{sTUv}})) > 0$, where G is the average genic value among those genes at the same locus whose expression controls PGE in the focal individual's father, and

G' is the average genic value among those genes at the same locus whose expression controls PGE in the males contributing offspring to the focal individual's mating group. Here: $\mathrm{d}\mu/\mathrm{d}G = \mathrm{d}\mu'/\mathrm{d}G' = 1$; $\mathrm{d}G/\mathrm{d}g_{\mathrm{sTUv}} = q_{\mathrm{sTUv}}$ is the consanguinity between the focal class-sTUv gene and the genes, residing at the same locus, who control PGE in the father of the focal gene's carrier; and $\mathrm{d}G'/\mathrm{d}g_{\mathrm{sTUv}} = q'_{\mathrm{sTUv}}$ is the consanguinity between the focal class-sTUv gene and the genes, residing at the same locus, who control PGE in the males who contribute offspring to the focal individual's mating group. Note that, because $\mathrm{d}W_{\mathrm{fTUv}}/\mathrm{d}\mu' = 0$ for all classes of genes in females, we need only calculate consanguinities q'_{mTUv} for classes of genes in males. Again, the consanguinities will depend upon which class or classes of genes control PGE in males -- see the Consanguinity section below, and Table A1.1.2, for details.

Consanguinity – The coefficient of inbreeding is the consanguinity of mating partners. This may be expressed as $\rho = a((1/4) \times (1+\rho)/2 + (1/2) \times \rho + (1/4) \times \rho)$. That is: with probability a the male and female share the same mother; so with probability ¼ we pick the maternal-origin genes from both individuals and hence their consanguinity is simply the consanguinity of their mother to herself, or $(1+\rho)/2$; and with probability ½ we pick the maternal-origin gene from one of the mating partners and the paternal-origin gene from the other, in which case their consanguinity is that of mating partners, or ρ; and with probability ¼ we pick the paternal-origin genes from both individuals, in which case their consanguinity is that of two males in the same mating group, or ρ. Solving this equation obtains $\rho = a/(8-7a)$, so that there is full outbreeding in the absence of sib mating ($\rho = 0$ when $a = 0$) and there is full inbreeding if all mating is between sibs ($\rho = 1$ when $a = 1$). From this coefficient can be defined other consanguinities between mating partners: $\rho_{\mathrm{M-}} = a((1/2) \times (1+\rho)/2 + (1/2) \times \rho)$, from the perspective of the female's maternal-origin gene; $\rho_{\mathrm{P-}} = a\,\rho$, from the perspective of the female's paternal-origin gene; $\rho_{\mathrm{-M}} = a((1/2) \times (1+\rho)/2 + (1/2) \times \rho)$, from the perspective of the male's maternal-origin gene; $\rho_{\mathrm{-P}} = a\,\rho$, from the perspective of the male's paternal-origin gene; $\rho_{\mathrm{MM}} = a\,(1+\rho)/2$, from the perspective of both mating partners' maternal genes; $\rho_{\mathrm{MP}} = a\,\rho$, from the perspective of the female's maternal gene and the male's paternal gene; $\rho_{\mathrm{PM}} = a\,\rho$, from the perspective of the female's paternal gene and the male's maternal gene; and $\rho_{\mathrm{PP}} = a\,\rho$, from the perspective of both mating partners' paternal genes. And these coefficients define all the consanguinities needed to solve the model (listed in Tables A1.1.1 & A1.1.2).

Potential for PGE – The condition for increase in PGE is $\mathrm{d}W/\mathrm{d}g > 0$. The left-hand side of this inequality is the marginal fitness and, above, we have calculated these for female PGE and for male PGE, for different scenarios regarding which genes control PGE. Evaluating the marginal fitnesses at $\phi = \phi' = \bar{\phi} = \mu = \mu' = \bar{\mu} = 0$, the inequalities give the condition for invasion of female or male PGE in a non-PGE population. Setting the marginal fitness for female PGE equal to zero, and solving for α, obtains the threshold cost of female PGE γ such that invasion will occur if $\alpha < \gamma$ and invasion will not occur if $\alpha > \gamma$. This defines the potential for female PGE. If $\gamma > 0$, then it is possible for costly PGE to invade (provided the cost is sufficiently small),

Recipient Class	Actor Class A	A_{Mat}	A_{Pat}	A_{Mot}	A_{Fat}
fMM1	(1+ρ)/2	1	ρ	(1+ρ)/2	ρ
fMM2	ρ_{-M}	ρ_{MM}	ρ_{PM}	a(1+ρ)/2	a ρ
fMP1	(1+ρ)/2	1	ρ	(1+ρ)/2	ρ
fMP2	ρ_{-P}	ρ_{MP}	ρ_{PP}	a ρ	a ρ
fPM1	(1+ρ)/2	ρ	1	ρ	(1+ρ)/2
fPM2	ρ_{-M}	ρ_{MM}	ρ_{PM}	a(1+ρ)/2	a ρ
fPP1	(1+ρ)/2	ρ	1	ρ	(1+ρ)/2
fPP2	ρ_{-P}	ρ_{MP}	ρ_{PP}	a ρ	a ρ
mMM1	(1+ρ)/2	1	ρ	(1+ρ)/2	ρ
mMM2	ρ_{-M}	ρ_{MM}	ρ_{PM}	a(1+ρ)/2	a ρ
mMP1	(1+ρ)/2	1	ρ	(1+ρ)/2	ρ
mMP2	ρ_{-P}	ρ_{MP}	ρ_{PP}	a ρ	a ρ
mPM1	(1+ρ)/2	ρ	1	ρ	(1+ρ)/2
mPM2	ρ_{-M}	ρ_{MM}	ρ_{PM}	a(1+ρ)/2	a ρ
mPP1	(1+ρ)/2	ρ	1	ρ	(1+ρ)/2
mPP2	ρ_{-P}	ρ_{MP}	ρ_{PP}	a ρ	a ρ

Table A1.1.1. Consanguinities for the female PGE invasion analysis: autosomal genes. The five autosomal actor classes are the female's autosomal genes (A), her maternal-origin autosomal genes (A_{Mat}), her paternal-origin autosomal genes (A_{Pat}), her mother's autosomal genes (A_{Mot}) and her father's autosomal genes (A_{Fat}). Shown here are their consanguinities p_{sTUv} to each of the recipient classes sTUv among the focal female's offspring.

and if $\gamma < 0$, then it is possible that beneficial PGE (i.e. providing a viability benefit rather than a viability cost) may not invade (provided the benefit is sufficiently small). Thus, the potential γ provides a quantitative measure of the extent to which a particular class of genes desires PGE in females. Similarly, setting the marginal fitness for male PGE to zero, and solving for β, obtains the potential for male PGE γ. These potentials are illustrated in Figure 3 of the main text.

1.2. X-linked genes

Classes – There are 4 classes of individual, classified according to their sex s ∈ {f,m} and the grandparent of origin of their maternal-origin gene T ∈ {M,P}. If the individual is female, then all of her paternal-origin X-linked genes are derived from her paternal grandmother. And if the individual is male, he has no paternal-origin X-linked genes. Thus, each class is notated in the form sTU, where U ∈ {M,-}, i.e. fMM, fPM, mM- and mP-.

Survival – The probabilities of survival for each class are: $S_{fMM}(\phi,\mu,\alpha,\beta) = (1/4)(1-\phi)(1-\mu) + (1/2)\phi(1-\mu)(1-\alpha) + (1/2)(1-\phi)\mu(1-\beta) + \phi\,\mu\,(1-\alpha)(1-\beta)$; $S_{fPM}(\phi,\mu,\alpha,\beta) =$

Recipient Class	Actor Class<br>A	A_{Mat}	A_{Pat}	A_{Mot}	A_{Fat}
fMM1	ρ_{M-}	ρ_{MM}	ρ_{MP}	$a(1+\rho)/2$	$a\,\rho$
fMM2	$(1+\rho)/2$	1	ρ	$(1+\rho)/2$	ρ
fMP1	ρ_{M-}	ρ_{MM}	ρ_{MP}	$a(1+\rho)/2$	$a\,\rho$
fMP2	$(1+\rho)/2$	ρ	1	ρ	$(1+\rho)/2$
fPM1	ρ_{P-}	ρ_{PM}	ρ_{PP}	$a\,\rho$	$a\,\rho$
fPM2	$(1+\rho)/2$	1	ρ	$(1+\rho)/2$	ρ
fPP1	ρ_{P-}	ρ_{PM}	ρ_{PP}	$a\,\rho$	$a\,\rho$
fPP2	$(1+\rho)/2$	ρ	1	ρ	$(1+\rho)/2$
mMM1	ρ_{M-}	ρ_{MM}	ρ_{MP}	$a(1+\rho)/2$	$a\,\rho$
mMM2	$(1+\rho)/2$	1	ρ	$(1+\rho)/2$	ρ
mMP1	ρ_{M-}	ρ_{MM}	ρ_{MP}	$a(1+\rho)/2$	$a\,\rho$
mMP2	$(1+\rho)/2$	ρ	1	ρ	$(1+\rho)/2$
mPM1	ρ_{P-}	ρ_{PM}	ρ_{PP}	$a\,\rho$	$a\,\rho$
mPM2	$(1+\rho)/2$	1	ρ	$(1+\rho)/2$	ρ
mPP1	ρ_{P-}	ρ_{PM}	ρ_{PP}	$a\,\rho$	$a\,\rho$
mPP2	$(1+\rho)/2$	ρ	1	ρ	$(1+\rho)/2$
mMM1′	$a\,\rho_{M-}$	$a\,\rho_{MM}$	$a\,\rho_{MP}$	$a^2(1+\rho)/2$	$a^2\,\rho$
mMM2′	$a\,\rho_{-M}$	$a\,\rho_{MM}$	$a\,\rho_{MP}$	$a^2(1+\rho)/2$	$a^2\,\rho$
mMP1′	$a\,\rho_{M-}$	$a\,\rho_{MM}$	$a\,\rho_{MP}$	$a^2(1+\rho)/2$	$a^2\,\rho$
mMP2′	$a\,\rho_{-P}$	$a\,\rho_{MP}$	$a\,\rho_{PP}$	$a^2\,\rho$	$a^2\,\rho$
mPM1′	$a\,\rho_{P-}$	$a\,\rho_{PM}$	$a\,\rho_{PP}$	$a^2\,\rho$	$a^2\,\rho$
mPM2′	$a\,\rho_{-M}$	$a\,\rho_{MM}$	$a\,\rho_{MP}$	$a^2(1+\rho)/2$	$a^2\,\rho$
mPP1′	$a\,\rho_{P-}$	$a\,\rho_{PM}$	$a\,\rho_{PP}$	$a^2\,\rho$	$a^2\,\rho$
mPP2′	$a\,\rho_{P-}$	$a\,\rho_{MP}$	$a\,\rho_{PP}$	$a^2\,\rho$	$a^2\,\rho$

Table A1.1.2. Consanguinities for the male PGE invasion analysis: autosomal genes. The five autosomal actor classes are the male's autosomal genes (A), his maternal-origin autosomal genes (A_{Mat}), his paternal-origin autosomal genes (A_{Pat}), his mother's autosomal genes (A_{Mot}) and his father's autosomal genes (A_{Fat}). Shown here are their consanguinities q_{sTUv} to each of the recipient classes sTUv among the male's offspring, and the consanguinities q'_{mTUv} to each of the recipient classes mTUv′, among the males competing for mates with the focal male's sons.

$(1/4)(1-\phi)(1-\mu) + (1/2)(1-\phi)\mu(1-\beta)$; $S_{mM-}(\phi,\mu,\alpha,\beta) = (1/4)(1-\phi)(1-\mu) + (1/2)\phi(1-\mu)(1-\alpha)$; and $S_{mP-}(\phi,\mu,\alpha,\beta) = (1/4)(1-\phi)(1-\mu)$.

Reproductive success – Contingent upon survival, expected reproductive success is $R_{fTU}(\phi',\mu',\alpha,\beta) = 1$ for females and $R_{mTU}(\phi',\mu',\alpha,\beta) = (1-z(\phi',\mu',\alpha,\beta))/z(\phi',\mu',\alpha,\beta)$ for males, where $z(\phi',\mu',\alpha,\beta) = (S_{mM-}(\phi',\mu',\alpha,\beta) + S_{mP-}(\phi',\mu',\alpha,\beta))/(S_{fMM}(\phi',\mu',\alpha,\beta) + S_{fPM}(\phi',\mu',\alpha,\beta) + S_{mM-}(\phi',\mu',\alpha,\beta) + S_{mP-}(\phi',\mu',\alpha,\beta))$.

Fitness – Expected fitness is given by the product of survival to maturity and expected reproductive success contingent upon surviving to maturity, i.e.

$w_{\mathrm{sTU}}(\phi,\phi',\mu,\mu',\alpha,\beta) = S_{\mathrm{sTU}}(\phi,\mu,\alpha,\beta)\times R_{\mathrm{sTU}}(\phi',\mu',\alpha,\beta)$. As before, because PGE is vanishingly rare in the population, the average fitness among all individuals of a particular class is given by $\bar{w}_{\mathrm{sTU}}(\alpha,\beta) = w_{\mathrm{sTU}}(0,0,0,0,\alpha,\beta)$, and each individual's fitness can be expressed relative to the average of their class, by $W_{\mathrm{sTU}}(\phi,\phi',\mu,\mu',\alpha,\beta) = w_{\mathrm{sTU}}(\phi,\phi',\mu,\mu',\alpha,\beta)/\ \bar{w}_{\mathrm{sTU}}(\alpha,\beta)$.

Gene fitness –There are 6 classes of gene, because individuals of each of the two female classes carry separate maternal-origin and paternal-origin genes, whereas individuals of each of the two male classes carry only maternal-origin genes. As before, gene classes are denoted in the form sTUv, where $v \in \{1,2\}$ according to whether the gene is of maternal (v = 1) or paternal (v = 2) origin. The relative fitness of a gene in a female is given by $W_{\mathrm{fTP1}}(\phi,\phi',\mu,\mu',\alpha,\beta) = W_{\mathrm{fTP2}}(\phi,\phi',\mu,\mu',\alpha,\beta) = W_{\mathrm{fTP}}(\phi,\phi',\mu,\mu',\alpha,\beta)$ and the relative fitness of a gene in a male is given by $W_{\mathrm{mT\text{-}1}}(\phi,\phi',\mu,\mu',\alpha,\beta) = W_{\mathrm{mT\text{-}}}(\phi,\phi',\mu,\mu',\alpha,\beta)$.

Reproductive value – As before, each gene's class in any generation may provide some information and some uncertainty about that gene's origin in the previous generation. For example, genes of class fMM1 in the present generation are derived from genes of class fTM1 in the previous generation, where T = M or P with equal probability. Consequently, the fMM1 gene derived from a fMM1 gene with probability $\wp_{\mathrm{fMM1}\leftarrow\mathrm{fMM1}} = ½$ and from a fPM1 gene with probability $\wp_{\mathrm{fMM1}\leftarrow\mathrm{fPM1}} = ½$. The corresponding system of 6 linear equations may be written in linear algebraic form as $\mathbf{c} = \mathbf{c}\bullet\mathbf{G}$, where $\mathbf{c} = \{c_{\mathrm{fMM1}},c_{\mathrm{fMM2}},c_{\mathrm{fPM1}},c_{\mathrm{fPM2}},c_{\mathrm{mM\text{-}1}},c_{\mathrm{mP\text{-}1}}\}$ is the vector of class reproductive values and $\mathbf{G}$ is the gene-flow matrix. Solving obtains $c_{\mathrm{sTUv}} = 1/6$ for all gene classes.

PGE in females – Following the same procedure as before, the condition for increase in female PGE is $c_{\mathrm{fMM1}}\ ((\mathrm{d}W_{\mathrm{fMM1}}/\mathrm{d}\phi) \times (\mathrm{d}\phi/\mathrm{d}G) \times (\mathrm{d}G/\mathrm{d}g_{\mathrm{fMM1}})) + c_{\mathrm{fMM2}}\ ((\mathrm{d}W_{\mathrm{fMM2}}/\mathrm{d}\phi) \times (\mathrm{d}\phi/\mathrm{d}G) \times (\mathrm{d}G/\mathrm{d}g_{\mathrm{fMM2}})) + c_{\mathrm{fPM1}}\ ((\mathrm{d}W_{\mathrm{fPM1}}/\mathrm{d}\phi) \times (\mathrm{d}\phi/\mathrm{d}G) \times (\mathrm{d}G/\mathrm{d}g_{\mathrm{fPM1}})) + c_{\mathrm{fPM2}}\ ((\mathrm{d}W_{\mathrm{fPM2}}/\mathrm{d}\phi) \times (\mathrm{d}\phi/\mathrm{d}G) \times (\mathrm{d}G/\mathrm{d}g_{\mathrm{fPM2}})) + c_{\mathrm{mM\text{-}1}}\ ((\mathrm{d}W_{\mathrm{mM\text{-}1}}/\mathrm{d}\phi) \times (\mathrm{d}\phi/\mathrm{d}G) \times (\mathrm{d}G/\mathrm{d}g_{\mathrm{mM\text{-}1}})) + c_{\mathrm{mP\text{-}1}}\ ((\mathrm{d}W_{\mathrm{mP\text{-}1}}/\mathrm{d}\phi) \times (\mathrm{d}\phi/\mathrm{d}G) \times (\mathrm{d}G/\mathrm{d}g_{\mathrm{mP\text{-}1}})) > 0$. Once again: $\mathrm{d}\phi/\mathrm{d}G$ is an arbitrary mapping between genic value and phenotypic value, and can be set to 1; and $\mathrm{d}G/\mathrm{d}g_{\mathrm{sTUv}} = p_{\mathrm{sTUv}}$ is the consanguinity between the focal class-sTUv gene and the genes, residing at the same locus, who control PGE in the mother of the focal gene's carrier.

PGE in males – Similarly, the condition for increase in male PGE is $c_{\mathrm{fMM1}}\ ((\mathrm{d}W_{\mathrm{fMM1}}/\mathrm{d}\mu) \times (\mathrm{d}\mu/\mathrm{d}G) \times (\mathrm{d}G/\mathrm{d}g_{\mathrm{fMM1}})) + c_{\mathrm{fMM2}}\ ((\mathrm{d}W_{\mathrm{fMM2}}/\mathrm{d}\mu) \times (\mathrm{d}\mu/\mathrm{d}G) \times (\mathrm{d}G/\mathrm{d}g_{\mathrm{fMM2}})) + c_{\mathrm{fPM1}}\ ((\mathrm{d}W_{\mathrm{fPM1}}/\mathrm{d}\mu) \times (\mathrm{d}\mu/\mathrm{d}G) \times (\mathrm{d}G/\mathrm{d}g_{\mathrm{fPM1}})) + c_{\mathrm{fPM2}}\ ((\mathrm{d}W_{\mathrm{fPM2}}/\mathrm{d}\mu) \times (\mathrm{d}\mu/\mathrm{d}G) \times (\mathrm{d}G/\mathrm{d}g_{\mathrm{fPM2}})) + c_{\mathrm{mM\text{-}1}}\ ((\mathrm{d}W_{\mathrm{mM\text{-}1}}/\mathrm{d}\mu) \times (\mathrm{d}\mu/\mathrm{d}G) \times (\mathrm{d}G/\mathrm{d}g_{\mathrm{mM\text{-}1}}) + (\mathrm{d}W_{\mathrm{mM\text{-}1}}/\mathrm{d}\mu') \times (\mathrm{d}\mu'/\mathrm{d}G') \times (\mathrm{d}G'/\mathrm{d}g_{\mathrm{mM\text{-}1}})) + c_{\mathrm{mP\text{-}1}}\ ((\mathrm{d}W_{\mathrm{mP\text{-}1}}/\mathrm{d}\mu) \times (\mathrm{d}\mu/\mathrm{d}G) \times (\mathrm{d}G/\mathrm{d}g_{\mathrm{mP\text{-}1}}) + (\mathrm{d}W_{\mathrm{mP\text{-}1}}/\mathrm{d}\mu') \times (\mathrm{d}\mu'/\mathrm{d}G') \times (\mathrm{d}G'/\mathrm{d}g_{\mathrm{mP\text{-}1}})) > 0$, where: $\mathrm{d}\mu/\mathrm{d}G = \mathrm{d}\mu'/\mathrm{d}G' = 1$; $\mathrm{d}G/\mathrm{d}g_{\mathrm{sTUv}} = q_{\mathrm{sTUv}}$ is the consanguinity between the focal class-sTUv gene and the genes, residing at the same locus, who control PGE in the father of the focal gene's carrier; and $\mathrm{d}G'/\mathrm{d}g_{\mathrm{mTUv}} = q'_{\mathrm{mTUv}}$ is the consanguinity between the focal class-sTUv gene and

the genes, residing at the same locus, who control PGE in the males who contribute offspring to the focal individual's mating group.

Consanguinity – The coefficient of inbreeding for X-linked genes is $\rho^* = a((1/2) \times (1+\rho^*)/2 + (1/2) \times \rho^*)$. That is: with probability a the male and female share the same mother; so with probability ½ we pick the maternal-origin genes from the female and hence their consanguinity is simply the consanguinity of their mother to herself, or $(1+\rho^*)/2$; and with probability ½ we pick the paternal-origin gene from the female, in which case their consanguinity is that of mating partners, or ρ^*. Solving this equation obtains $\rho^* = a/(4-3a)$. From this coefficient can be defined other consanguinities between mating partners: $\rho^*_{M-} = a(1+\rho^*)/2$, from the perspective of the female's maternal-origin gene; and $\rho^*_{P-} = a\ \rho^*$, from the perspective of the female's paternal-origin gene. Moreover, the consanguinity between two males in the same mating group is $\psi^* = a(1+\rho^*)/2$. These coefficients define all the consanguinities needed to solve the model (listed in Tables A1.2.1 & A1.2.2).

Potential for PGE – The potentials for female and male PGE are calculated as described above. These are illustrated in Figure 3 of the main text.

1.3. Y-linked genes

Classes – There are 2 classes of individual, classified according to their sex $s \in \{f,m\}$. If the individual is female, then she has no Y-linked genes. And if the individual is male, then all of his Y-linked genes are paternal in origin (and came from his paternal grandfather). Thus, class is notated in the form $s \in \{f,m\}$.

Survival – The probabilities of survival for each class are $S_f(\phi,\mu,\alpha,\beta) = (1/2)(1-\phi)(1-\mu) + (1/2)\phi(1-\mu)(1-\alpha) + (1-\phi)\mu(1-\beta) + \phi\ \mu\ (1-\alpha)(1-\beta)$ and $S_m(\phi,\mu,\alpha,\beta) = (1/2)(1-\phi)(1-\mu) + (1/2)\phi(1-\mu)(1-\alpha)$.

Reproductive success – Contingent upon survival, expected reproductive success is $R_f(\phi',\mu',\alpha,\beta) = 1$ for females and $R_m(\phi',\mu',\alpha,\beta) = (1-z(\phi',\mu',\alpha,\beta))/z(\phi',\mu',\alpha,\beta)$ for males, where $z(\phi',\mu',\alpha,\beta) = S_m(\phi',\mu',\alpha,\beta)/(S_f(\phi',\mu',\alpha,\beta) + S_m(\phi',\mu',\alpha,\beta))$.

Fitness – Expected fitness is given by the product of survival to maturity and expected reproductive success contingent upon surviving to maturity, i.e. $w_s(\phi,\phi',\mu,\mu',\alpha,\beta) = S_s(\phi,\mu,\alpha,\beta) \times R_s(\phi',\mu',\alpha,\beta)$. As before, because PGE is vanishingly rare in the population, the average fitness among all individuals of a particular class is given by $\bar{w}_s(\alpha,\beta) = w_s(0,0,0,0,\alpha,\beta)$, and each individual's fitness can be expressed relative to the average of their class, by $W_s(\phi,\phi',\mu,\mu',\alpha,\beta) = w_s(\phi,\phi',\mu,\mu',\alpha,\beta)/\ \bar{w}_s(\alpha,\beta)$.

Gene fitness – There is only one class of Y-linked gene, because females carry no Y-linked genes and males carry one class of Y-linked genes. Accordingly, the relative fitness of a Y-linked gene in a male is simply $W_m(\phi,\phi',\mu,\mu',\alpha,\beta)$.

Recipient Class	Actor Class X	X_{Mat}	X_{Pat}	X_{Mot}	X_{Fat}
fMM1	$(1+\rho^*)/2$	1	ρ^*	$(1+\rho^*)/2$	ρ^*
fMM2	ρ^*	ρ^*_{M-}	ρ^*_{P-}	$a(1+\rho^*)/2$	$a\,\rho^*$
fPM1	$(1+\rho^*)/2$	ρ^*	1	ρ^*	1
fPM2	ρ^*	ρ^*_{M-}	ρ^*_{P-}	$a(1+\rho^*)/2$	$a\,\rho^*$
mM-1	$(1+\rho^*)/2$	1	ρ^*	$(1+\rho^*)/2$	ρ^*
mP-1	$(1+\rho^*)/2$	ρ^*	1	ρ^*	1

Table A1.2.1. Consanguinities for the female PGE invasion analysis: X-linked genes. The five X-linked actor classes are the female's X-linked genes (X), her maternal-origin X-linked genes (X_{Mat}), her paternal-origin X-linked genes (X_{Pat}), her mother's X-linked genes (X_{Mot}) and her father's X-linked genes (X_{Fat}). Shown here are their consanguinities p_{sTUv} to each of the recipient classes sTUv among the focal female's offspring.

Recipient Class	Actor Class X	X_{Mot}	X_{Fat}
fMM1	ρ^*_{M-}	$a(1+\rho^*)/2$	$a\,\rho^*$
fMM2	1	$(1+\rho^*)/2$	ρ^*
fPM1	ρ^*_{P-}	$a\,\rho^*$	$a\,\psi^*$
fPM2	1	$(1+\rho^*)/2$	ρ^*
mM-1	ρ^*_{M-}	$a(1+\rho^*)/2$	$a\,\rho^*$
mP-1	ρ^*_{P-}	$a\,\rho^*$	$a\,\psi^*$
mM-1′	$a\,\rho^*_{M-}$	$a^2(1+\rho^*)/2$	$a^2\,\rho^*$
mP-1′	$a\,\rho^*_{P-}$	$a^2\,\rho^*$	$a^2\,\psi^*$

Table A1.2.2. Consanguinities for the male PGE invasion analysis: X-linked genes. The three X-linked actor classes are the male's X-linked genes (X), his mother's X-linked genes (X_{Mot}) and his father's X-linked genes (X_{Fat}). Shown here are their consanguinities q_{sTUv} to each of the recipient classes sTUv among the male's offspring, and the consanguinities q'_{mTUv} to each of the recipient classes mTUv′, among the males competing for mates with the focal male's sons.

Reproductive value – Since there is only one class of Y-linked gene, all reproductive value belongs to this class: $c_m = 1$.

PGE in females – Following the same procedure as before, the condition for increase in female PGE is $c_m\,((\mathrm{d}W_m/\mathrm{d}\phi) \times (\mathrm{d}\phi/\mathrm{d}G) \times (\mathrm{d}G/\mathrm{d}g_m)) > 0$. Once again: $\mathrm{d}\phi/\mathrm{d}G$ is an arbitrary mapping between genic value and phenotypic value, and can be set to 1; and $\mathrm{d}G/\mathrm{d}g_m = p_m$ is the consanguinity between the focal gene and the genes, residing at the same locus, who control PGE in the mother of the focal gene's carrier.

PGE in males – Similarly, the condition for increase in male PGE is $c_m\,((\mathrm{d}W_m/\mathrm{d}\mu) \times (\mathrm{d}\mu/\mathrm{d}G) \times (\mathrm{d}G/\mathrm{d}g_m) + (\mathrm{d}W_m/\mathrm{d}\mu') \times (\mathrm{d}\mu'/\mathrm{d}G') \times (\mathrm{d}G'/\mathrm{d}g_m)) > 0$, where: $\mathrm{d}\mu/\mathrm{d}G =$

$d\mu'/dG' = 1$; $dG/dg_m = q_m$ is the consanguinity between the focal class-sTUv gene and the genes, residing at the same locus, who control PGE in the father of the focal gene's carrier; and $dG'/dg_m = q'_m$ is the consanguinity between the focal class-sTUv gene and the genes, residing at the same locus, who control PGE in the males who contribute offspring to the focal individual's mating group.

Consanguinity – Coefficients of inbreeding are undefined for Y-linked genes, because females do not carry any such genes. The consanguinity between two males in the same mating group is $\psi^{**} = a\psi^{**}$; that is, with probability a they share the same mother, and hence their consanguinity is that of their fathers, i.e. two males in the same mating group. Solving yields $\psi^{**} = 0$ for all $0 < a < 1$. This coefficient defines all the consanguinities needed to solve the model (listed in Tables A1.3.1 & A1.3.2).

Potential for PGE – The potentials for female and male PGE are calculated as described above. These are illustrated in Figure 3 of the main text.

2. MGE in females and/or males under XY/XO inheritance: invasion analysis

2.1. Autosomal genes

Classes – There are 8 classes of individual, classified according to their sex $s \in \{f,m\}$, the grandparent of origin of their maternal-origin gene $T \in \{M,P\}$ and the grandparent of origin of their paternal-origin gene $U \in \{M,P\}$. Each class is notated in the form sTU, i.e. fMM, fMP, fPM, fPP, mMM, mMP, mPM and mPP.

Survival – Denoting the probability that the zygote's mother exhibited MGE by ϕ, the probability that the zygote's father exhibited MGE by μ, the viability cost associated with female MGE by α and the viability cost associated with male MGE by β, the probability that each potential zygote survives to maturity is: $S_{fMM}(\phi,\mu,\alpha,\beta) = (1/8)(1-\phi)(1-\mu)$; $S_{fMP}(\phi,\mu,\alpha,\beta) = (1/8)(1-\phi)(1-\mu)$; $S_{fPM}(\phi,\mu,\alpha,\beta) = (1/8)(1-\phi)(1-\mu) + (1/4)\phi(1-\mu)(1-\alpha)$; $S_{fPP}(\phi,\mu,\alpha,\beta) = (1/8)(1-\phi)(1-\mu) + (1/4)\phi(1-\mu)(1-\alpha)$; $S_{mMM}(\phi,\mu,\alpha,\beta) = (1/8)(1-\phi)(1-\mu)$; $S_{mMP}(\phi,\mu,\alpha,\beta) = (1/8)(1-\phi)(1-\mu) + (1/2)(1-\phi)\mu(1-\beta)$; $S_{mPM}(\phi,\mu,\alpha,\beta) = (1/8)(1-\phi)(1-\mu) + (1/4)\phi(1-\mu)(1-\alpha)$; and $S_{mPP}(\phi,\mu,\alpha,\beta) = (1/8)(1-\phi)(1-\mu) + (1/4)\phi(1-\mu)(1-\alpha) + (1/2)(1-\phi)\mu(1-\beta) + \phi\mu(1-\alpha)(1-\beta)$.

Reproductive success – Upon survival to maturity, every female achieves a unit of expected reproductive success, and every male achieves $(1-z(\phi',\mu',\alpha,\beta))/z(\phi',\mu',\alpha,\beta)$ units of expected reproductive success, where: $z(\phi',\mu',\alpha,\beta) = (\sum_{T\in\{M,P\},U\in\{M,P\}} S_{mTU}(\phi',\mu',\alpha,\beta)) / (\sum_{s\in\{f,m\},T\in\{M,P\},U\in\{M,P\}} S_{sTU}(\phi',\mu',\alpha,\beta))$ is the sex ratio (proportion male) in his mating group; ϕ' is the level of MGE among the females contributing offspring to the mating group; and μ' is the level of MGE among the fathers contributing offspring to the mating group. Thus, contingent upon survival, expected reproductive success is $R_{fTU}(\phi',\mu',\alpha,\beta) = 1$ for females and $R_{mTU}(\phi',\mu',\alpha,\beta) = (1-z(\phi',\mu',\alpha,\beta))/z(\phi',\mu',\alpha,\beta)$ for males.

Recipient Class	Actor Class Y_{Fat}
m	$a\,\psi^{**}$

Table A1.3.1. Consanguinities for the female PGE invasion analysis: Y-linked genes. The only Y-linked actor class is the female's father's Y-linked genes (Y_{Fat}). Shown here are their consanguinity p_m to the single recipient class m among the focal female's sons.

Recipient Class	Actor Class Y	Y_{Fat}
m	1	1
m′	$a\,\psi^{**}$	$a\,\psi^{**}$

Table A1.3.2. Consanguinities for the male PGE invasion analysis: Y-linked genes. The two Y-linked actor classes are the male's own Y-linked genes (Y) and the Y-linked genes of his father (Y_{Fat}). Shown here are their consanguinity q_m to the recipient class m among the focal male's sons, and the consanguinity q'_m to the recipient class m′ in the males who compete for mates with the focal male's sons.

Fitness – Expected fitness is given by the product of survival to maturity and expected reproductive success contingent upon surviving to maturity, i.e. $w_{sTU}(\phi,\phi',\mu,\mu',\alpha,\beta) = S_{sTU}(\phi,\mu,\alpha,\beta)\times R_{sTU}(\phi',\mu',\alpha,\beta)$. Because MGE is vanishingly rare in the population, the average fitness among all individuals of a particular class is given by $\bar{w}_{sTU}(\alpha,\beta) = w_{sTU}(0,0,0,0,\alpha,\beta)$, and each individual's fitness can be expressed relative to the average of their class, by $W_{sTU}(\phi,\phi',\mu,\mu',\alpha,\beta) = w_{sTU}(\phi,\phi',\mu,\mu',\alpha,\beta)/\ \bar{w}_{sTU}(\alpha,\beta)$.

Gene fitness –There are 16 classes of gene, because every class of individual carries one maternal-origin gene and one paternal-origin gene. Thus, gene classes are denoted in the form sTUv, where $v \in \{1,2\}$ according to whether the gene is of maternal (v = 1) or paternal (v = 2) origin. The fitness of a gene, defined in terms of its probability of being transmitted to a potential (rather than a realised) zygote, is proportional to the fitness of the individual who carries it. Consequently, the relative fitness of a gene is equal to the relative fitness of its carrier: $W_{sTU1}(\phi,\phi',\mu,\mu',\alpha,\beta) = W_{sTU2}(\phi,\phi',\mu,\mu',\alpha,\beta) = W_{sTU}(\phi,\phi',\mu,\mu',\alpha,\beta)$.

Reproductive value – Because class reproductive values are calculated in a population in which genome elimination is vanishingly rare, these are exactly the same as calculated in section 1.1, i.e. $c_{sTUv} = 1/16$ for all gene classes.

MGE in females – The condition for natural selection to favour an increase in MGE is $\sum_{s\in\{f,m\},T\in\{M,P\},U\in\{M,P\},v\in\{1,2\}} c_{sTUv}\ (dW_{sTUv}/d\phi) \times (d\phi/dG) \times (dG/dg_{sTUv}) > 0$, where G is the average genic value among those genes at the same locus whose expression controls MGE in the focal individual's mother. Consequently: $d\phi/dG$ is an arbitrary mapping

between genic value and phenotypic value, and can be set to 1; and $\mathrm{d}G/\mathrm{d}g_{\mathrm{sTUv}} = p_{\mathrm{sTUv}}$ is the consanguinity between the focal class-sTUv gene and the genes, residing at the same locus, who control MGE in the mother of the focal gene's carrier. The consanguinities will depend upon which class or classes of genes control MGE in females – and these are exactly the same as those listed in Table A1.1.1.

MGE in males – Similarly, the condition for increase in male MGE is $\sum_{\mathrm{s}\in\{\mathrm{f,m}\},\mathrm{T}\in\{\mathrm{M,P}\},\mathrm{U}\in\{\mathrm{M,P}\},\mathrm{v}\in\{1,2\}} c_{\mathrm{sTUv}}\left((\mathrm{d}W_{\mathrm{sTUv}}/\mathrm{d}\mu) \times (\mathrm{d}\mu/\mathrm{d}G) \times (\mathrm{d}G/\mathrm{d}g_{\mathrm{sTUv}}) + (\mathrm{d}W_{\mathrm{sTUv}}/\mathrm{d}\mu') \times (\mathrm{d}\mu'/\mathrm{d}G') \times (\mathrm{d}G'/\mathrm{d}g_{\mathrm{sTUv}})\right) > 0$, where G is the average genic value among those genes at the same locus whose expression controls MGE in the focal individual's father, and G' is the average genic value among those genes at the same locus whose expression controls MGE in the males contributing offspring to the focal individual's mating group. Here: $\mathrm{d}\mu/\mathrm{d}G = \mathrm{d}\mu'/\mathrm{d}G' = 1$; $\mathrm{d}G/\mathrm{d}g_{\mathrm{sTUv}} = q_{\mathrm{sTUv}}$ is the consanguinity between the focal class-sTUv gene and the genes, residing at the same locus, who control MGE in the father of the focal gene's carrier; and $\mathrm{d}G'/\mathrm{d}g_{\mathrm{sTUv}} = q'_{\mathrm{sTUv}}$ is the consanguinity between the focal class-sTUv gene and the genes, residing at the same locus, who control MGE in the males who contribute offspring to the focal individual's mating group. Note that, because $\mathrm{d}W_{\mathrm{fTUv}}/\mathrm{d}\mu' = 0$ for all classes of genes in females, we need only calculate consanguinities q'_{mTUv} for classes of genes in males. Again, the consanguinities will depend upon which class or classes of genes control PGE in males -- and these are exactly the same as those listed in Table A1.1.2.

Consanguinity – As noted above, because consanguinities are calculated in a population from which genomic elimination is absent, these are exactly the same as those listed in Tables A1.1.1 & A1.1.2.

Potential for MGE – The potential for female MGE and the potential for male MGE are calculated in the same way as before, and these are illustrated in Figure 3 of the main text.

2.2. X-linked genes

Classes – There are 4 classes of individual, classified according to their sex s ∈ {f,m} and the grandparent of origin of their maternal-origin gene T ∈ {M,P}. If the individual is female, then all of her paternal-origin X-linked genes are derived from her paternal grandmother. And if the individual is male, he has no paternal-origin X-linked genes. Thus, each class is notated in the form sTU, where U ∈ {M,-}, i.e. fMM, fPM, mM- and mP-.

Survival – The probabilities of survival for each class are: $S_{\mathrm{fMM}}(\phi,\mu,\alpha,\beta) = (1/4)(1-\phi)(1-\mu)$; $S_{\mathrm{fPM}}(\phi,\mu,\alpha,\beta) = (1/4)(1-\phi)(1-\mu) + (1/2)\phi(1-\mu)(1-\alpha)$; $S_{\mathrm{mM-}}(\phi,\mu,\alpha,\beta) = (1/4)(1-\phi)(1-\mu) + (1/2)(1-\phi)\mu(1-\beta)$; and $S_{\mathrm{mP-}}(\phi,\mu,\alpha,\beta) = (1/4)(1-\phi)(1-\mu) + (1/2)\phi(1-\mu)(1-\alpha) + (1/2)(1-\phi)\mu(1-\beta) + (1/2)\phi\mu(1-\alpha)(1-\beta)$.

Reproductive success – Contingent upon survival, expected reproductive success is $R_{\mathrm{fTU}}(\phi',\mu',\alpha,\beta) = 1$ for females and $R_{\mathrm{mTU}}(\phi',\mu',\alpha,\beta) = (1\text{-}z(\phi',\mu',\alpha,\beta))/z(\phi',\mu',\alpha,\beta)$ for males, where $z(\phi',\mu',\alpha,\beta) = (S_{\mathrm{mM-}}(\phi',\mu',\alpha,\beta) + S_{\mathrm{mP-}}(\phi',\mu',\alpha,\beta))/(S_{\mathrm{fMM}}(\phi',\mu',\alpha,\beta) + S_{\mathrm{fPM}}(\phi',\mu',\alpha,\beta) + S_{\mathrm{mM-}}(\phi',\mu',\alpha,\beta) + S_{\mathrm{mP-}}(\phi',\mu',\alpha,\beta))$.

Fitness – Expected fitness is given by the product of survival to maturity and expected reproductive success contingent upon surviving to maturity, i.e. $w_{\mathrm{sTU}}(\phi,\phi',\mu,\mu',\alpha,\beta) = S_{\mathrm{sTU}}(\phi,\mu,\alpha,\beta)\times R_{\mathrm{sTU}}(\phi',\mu',\alpha,\beta)$. As before, because MGE is vanishingly rare in the population, the average fitness among all individuals of a particular class is given by $\bar{w}_{\mathrm{sTU}}(\alpha,\beta) = w_{\mathrm{sTU}}(0,0,0,0,\alpha,\beta)$, and each individual's fitness can be expressed relative to the average of their class, by $W_{\mathrm{sTU}}(\phi,\phi',\mu,\mu',\alpha,\beta) = w_{\mathrm{sTU}}(\phi,\phi',\mu,\mu',\alpha,\beta)/\ \bar{w}_{\mathrm{sTU}}(\alpha,\beta)$.

Gene fitness –There are 6 classes of gene, because individuals of each of the two female classes carries separate maternal-origin and paternal-origin genes, whereas individuals of each of the two male classes carry only maternal-origin genes. As before, gene classes are denoted in the form sTUv, where v $\in$ {1,2} according to whether the gene is of maternal (v = 1) or paternal (v = 2) origin. The relative fitness of a gene in a female is given by $W_{\mathrm{fTP1}}(\phi,\phi',\mu,\mu',\alpha,\beta) = W_{\mathrm{fTP2}}(\phi,\phi',\mu,\mu',\alpha,\beta) = W_{\mathrm{fTP}}(\phi,\phi',\mu,\mu',\alpha,\beta)$ and the relative fitness of a gene in a male is given by $W_{\mathrm{mT-1}}(\phi,\phi',\mu,\mu',\alpha,\beta) = W_{\mathrm{mT-}}(\phi,\phi',\mu,\mu',\alpha,\beta)$.

Reproductive value – Because class reproductive values are calculated in a population in which genome elimination is vanishingly rare, these are exactly the same as calculated in section 1.2, i.e. $c_{\mathrm{sTUv}} = 1/6$ for all gene classes.

MGE in females – Following the same procedure as before, the condition for increase in female MGE is c_{fMM1} $((\mathrm{d}W_{\mathrm{fMM1}}/\mathrm{d}\phi) \times (\mathrm{d}\phi/\mathrm{d}G) \times (\mathrm{d}G/\mathrm{d}g_{\mathrm{fMM1}})) + c_{\mathrm{fMM2}}$ $((\mathrm{d}W_{\mathrm{fMM2}}/\mathrm{d}\phi) \times (\mathrm{d}\phi/\mathrm{d}G) \times (\mathrm{d}G/\mathrm{d}g_{\mathrm{fMM2}})) + c_{\mathrm{fPM1}}$ $((\mathrm{d}W_{\mathrm{fPM1}}/\mathrm{d}\phi) \times (\mathrm{d}\phi/\mathrm{d}G) \times (\mathrm{d}G/\mathrm{d}g_{\mathrm{fPM1}})) + c_{\mathrm{fPM2}}$ $((\mathrm{d}W_{\mathrm{fPM2}}/\mathrm{d}\phi) \times (\mathrm{d}\phi/\mathrm{d}G) \times (\mathrm{d}G/\mathrm{d}g_{\mathrm{fPM2}})) + c_{\mathrm{mM-1}}$ $((\mathrm{d}W_{\mathrm{mM-1}}/\mathrm{d}\phi) \times (\mathrm{d}\phi/\mathrm{d}G) \times (\mathrm{d}G/\mathrm{d}g_{\mathrm{mM-1}})) + c_{\mathrm{mP-1}}$ $((\mathrm{d}W_{\mathrm{mP-1}}/\mathrm{d}\phi) \times (\mathrm{d}\phi/\mathrm{d}G) \times (\mathrm{d}G/\mathrm{d}g_{\mathrm{mP-1}})) > 0$. Once again: $\mathrm{d}\phi/\mathrm{d}G$ is an arbitrary mapping between genic value and phenotypic value, and can be set to 1; and $\mathrm{d}G/\mathrm{d}g_{\mathrm{sTUv}} = p_{\mathrm{sTUv}}$ is the consanguinity between the focal class-sTUv gene and the genes, residing at the same locus, who control MGE in the mother of the focal gene's carrier.

MGE in males – Similarly, the condition for increase in male MGE is c_{fMM1} $((\mathrm{d}W_{\mathrm{fMM1}}/\mathrm{d}\mu) \times (\mathrm{d}\mu/\mathrm{d}G) \times (\mathrm{d}G/\mathrm{d}g_{\mathrm{fMM1}})) + c_{\mathrm{fMM2}}$ $((\mathrm{d}W_{\mathrm{fMM2}}/\mathrm{d}\mu) \times (\mathrm{d}\mu/\mathrm{d}G) \times (\mathrm{d}G/\mathrm{d}g_{\mathrm{fMM2}})) + c_{\mathrm{fPM1}}$ $((\mathrm{d}W_{\mathrm{fPM1}}/\mathrm{d}\mu) \times (\mathrm{d}\mu/\mathrm{d}G) \times (\mathrm{d}G/\mathrm{d}g_{\mathrm{fPM1}})) + c_{\mathrm{fPM2}}$ $((\mathrm{d}W_{\mathrm{fPM2}}/\mathrm{d}\mu) \times (\mathrm{d}\mu/\mathrm{d}G) \times (\mathrm{d}G/\mathrm{d}g_{\mathrm{fPM2}})) + c_{\mathrm{mM-1}}$ $((\mathrm{d}W_{\mathrm{mM-1}}/\mathrm{d}\mu) \times (\mathrm{d}\mu/\mathrm{d}G) \times (\mathrm{d}G/\mathrm{d}g_{\mathrm{mM-1}}) + (\mathrm{d}W_{\mathrm{mM-1}}/\mathrm{d}\mu') \times (\mathrm{d}\mu'/\mathrm{d}G') \times (\mathrm{d}G'/\mathrm{d}g_{\mathrm{mM-1}})) + c_{\mathrm{mP-1}}$ $((\mathrm{d}W_{\mathrm{mP-1}}/\mathrm{d}\mu) \times (\mathrm{d}\mu/\mathrm{d}G) \times (\mathrm{d}G/\mathrm{d}g_{\mathrm{mP-1}}) + (\mathrm{d}W_{\mathrm{mP-1}}/\mathrm{d}\mu') \times (\mathrm{d}\mu'/\mathrm{d}G') \times (\mathrm{d}G'/\mathrm{d}g_{\mathrm{mP-1}})) > 0$, where: $\mathrm{d}\mu/\mathrm{d}G = \mathrm{d}\mu'/\mathrm{d}G' = 1$; $\mathrm{d}G/\mathrm{d}g_{\mathrm{sTUv}} = q_{\mathrm{sTUv}}$ is the consanguinity between the focal class-sTUv gene and the genes, residing at the same locus, who control MGE in the father of the focal gene's carrier; and $\mathrm{d}G'/\mathrm{d}g_{\mathrm{mTUv}} = q'_{\mathrm{mTUv}}$ is the consanguinity between the focal class-sTUv gene and

the genes, residing at the same locus, who control MGE in the males who contribute offspring to the focal individual's mating group.

Consanguinity – As noted above, because consanguinities are calculated in a population from which genomic elimination is absent, these are exactly the same as those listed in Tables A1.2.1 & A1.2.2.

Potential for MGE – The potential for female MGE and the potential for male MGE are calculated in the same way as before, and these are illustrated in Figure 3 of the main text.

2.3. Y-linked genes

Classes – There are 2 classes of individual, classified according to their sex $s \in \{f,m\}$. If the individual is female, then she has no Y-linked genes. And if the individual is male, then all of his Y-linked genes are paternal in origin (and came from his paternal grandfather). Thus, class is notated in the form $s \in \{f,m\}$.

Survival – The probabilities of survival for each class are $S_f(\phi,\mu,\alpha,\beta) = (1/2)(1-\phi)(1-\mu) + (1/2)\phi(1-\mu)(1-\alpha)$ and $S_m(\phi,\mu,\alpha,\beta) = (1/2)(1-\phi)(1-\mu) + (1/2)\phi(1-\mu)(1-\alpha) + (1-\phi)\mu(1-\beta) + \phi\, \mu\, (1-\alpha)(1-\beta)$.

Reproductive success – Contingent upon survival, expected reproductive success is $R_f(\phi',\mu',\alpha,\beta) = 1$ for females and $R_m(\phi',\mu',\alpha,\beta) = (1-z(\phi',\mu',\alpha,\beta))/z(\phi',\mu',\alpha,\beta)$ for males, where $z(\phi',\mu',\alpha,\beta) = S_m(\phi',\mu',\alpha,\beta)/(S_f(\phi',\mu',\alpha,\beta) + S_m(\phi',\mu',\alpha,\beta))$.

Fitness – Expected fitness is given by the product of survival to maturity and expected reproductive success contingent upon surviving to maturity, i.e. $w_s(\phi,\phi',\mu,\mu',\alpha,\beta) = S_s(\phi,\mu,\alpha,\beta)\times R_s(\phi',\mu',\alpha,\beta)$. As before, because MGE is vanishingly rare in the population, the average fitness among all individuals of a particular class is given by $\bar{w}_s(\alpha,\beta) = w_s(0,0,0,0,\alpha,\beta)$, and each individual's fitness can be expressed relative to the average of their class, by $W_s(\phi,\phi',\mu,\mu',\alpha,\beta) = w_s(\phi,\phi',\mu,\mu',\alpha,\beta)/\ \bar{w}_s(\alpha,\beta)$.

Gene fitness – There is only one class of Y-linked gene, because females carry no Y-linked genes and males carry one class of Y-linked genes. Accordingly, the relative fitness of a Y-linked gene in a male is simply $W_m(\phi,\phi',\mu,\mu',\alpha,\beta)$.

Reproductive value – Since there is only one class of Y-linked gene, all reproductive value belongs to this class: $c_m = 1$.

MGE in females – Following the same procedure as before, the condition for increase in female MGE is $c_m\ ((\mathrm{d}W_m/\mathrm{d}\phi) \times (\mathrm{d}\phi/\mathrm{d}G) \times (\mathrm{d}G/\mathrm{d}g_m)) > 0$. Once again: $\mathrm{d}\phi/\mathrm{d}G$ is an arbitrary mapping between genic value and phenotypic value, and can be set to 1; and $\mathrm{d}G/\mathrm{d}g_m = p_m$ is the consanguinity between the focal gene and the genes, residing at the same locus, who control MGE in the mother of the focal gene's carrier.

MGE in males – Similarly, the condition for increase in male MGE is c_m $((dW_m/d\mu) \times (d\mu/dG) \times (dG/dg_m) + (dW_m/d\mu') \times (d\mu'/dG') \times (dG'/dg_m)) > 0$, where: $d\mu/dG = d\mu'/dG' = 1$; $dG/dg_m = q_m$ is the consanguinity between the focal class-sTUv gene and the genes, residing at the same locus, who control MGE in the father of the focal gene's carrier; and $dG'/dg_m = q'_m$ is the consanguinity between the focal class-sTUv gene and the genes, residing at the same locus, who control MGE in the males who contribute offspring to the focal individual's mating group.

Consanguinity – As noted above, because consanguinities are calculated in a population from which genomic elimination is absent, these are exactly the same as those listed in Tables A1.3.1 & A1.3.2.

Potential for MGE – The potential for female MGE and the potential for male MGE are calculated in the same way as before, and these are illustrated in Figure 3 of the main text.

3. PGE in females and/or males under ZW inheritance: invasion analysis

3.1. Autosomal genes

Classes – There are 8 classes of individual, classified according to their sex $s \in \{f,m\}$, the grandparent of origin of their maternal-origin gene $T \in \{M,P\}$ and the grandparent of origin of their paternal-origin gene $U \in \{M,P\}$. Each class is notated in the form sTU, i.e. fMM, fMP, fPM, fPP, mMM, mMP, mPM and mPP.

Survival – Picking a zygote at random, it has some probability of belonging to each of the 8 classes, depending on whether its parents exhibited genome elimination. We model this as if there were 8 potential zygotes, of which one is chosen to come into existence. Moreover, the realised zygote may not survive to maturity, owing to viability costs associated with the genome elimination behaviour of its parents. Denoting the probability that the zygote's mother exhibited PGE by ϕ, the probability that the zygote's father exhibited PGE by μ, the viability cost associated with female PGE by α and the viability cost associated with male PGE by β, the probability that each potential zygote survives to maturity is: $S_{fMM}(\phi,\mu,\alpha,\beta) = (1/8)(1-\phi)(1-\mu) + (1/2)\phi(1-\mu)(1-\alpha) + (1/4)(1-\phi)\mu(1-\beta) + \phi\,\mu\,(1-\alpha)(1-\beta)$; $S_{fMP}(\phi,\mu,\alpha,\beta) = (1/8)(1-\phi)(1-\mu) + (1/2)\phi(1-\mu)(1-\alpha)$; $S_{fPM}(\phi,\mu,\alpha,\beta) = (1/8)(1-\phi)(1-\mu) + (1/4)(1-\phi)\mu(1-\beta)$; $S_{fPP}(\phi,\mu,\alpha,\beta) = (1/8)(1-\phi)(1-\mu)$; $S_{mMM}(\phi,\mu,\alpha,\beta) = (1/8)(1-\phi)(1-\mu) + (1/4)(1-\phi)\mu(1-\beta)$; $S_{mMP}(\phi,\mu,\alpha,\beta) = (1/8)(1-\phi)(1-\mu)$; $S_{mPM}(\phi,\mu,\alpha,\beta) = (1/8)(1-\phi)(1-\mu)+ (1/4)(1-\phi)\mu(1-\beta)$; and $S_{mPP}(\phi,\mu,\alpha,\beta) = (1/8)(1-\phi)(1-\mu)$.

Reproductive success – Upon survival to maturity, every female achieves a unit of expected reproductive success, and every male achieves $(1-z(\phi',\mu',\alpha,\beta))/z(\phi',\mu',\alpha,\beta)$ units of expected reproductive success, where: $z(\phi',\mu',\alpha,\beta) = (\sum_{T\in\{M,P\},U\in\{M,P\}} S_{mTU}(\phi',\mu',\alpha,\beta)) / (\sum_{s\in\{f,m\},T\in\{M,P\},U\in\{M,P\}} S_{sTU}(\phi',\mu',\alpha,\beta))$ is the sex ratio (proportion male)

in his mating group; ϕ' is the level of PGE among the females contributing offspring to the mating group; and μ' is the level of PGE among the fathers contributing offspring to the mating group. Thus, contingent upon survival, expected reproductive success is $R_{\mathrm{fTU}}(\phi',\mu',\alpha,\beta) = 1$ for females and $R_{\mathrm{mTU}}(\phi',\mu',\alpha,\beta) = (1-z(\phi',\mu',\alpha,\beta))/z(\phi',\mu',\alpha,\beta)$ for males.

Fitness – Expected fitness is given by the product of survival to maturity and expected reproductive success contingent upon surviving to maturity, i.e. $w_{\mathrm{sTU}}(\phi,\phi',\mu,\mu',\alpha,\beta) = S_{\mathrm{sTU}}(\phi,\mu,\alpha,\beta)\times R_{\mathrm{sTU}}(\phi',\mu',\alpha,\beta)$. Because PGE is vanishingly rare in the population, the average fitness among all individuals of a particular class is given by $\bar{w}_{\mathrm{sTU}}(\alpha,\beta) = w_{\mathrm{sTU}}(0,0,0,0,\alpha,\beta)$, and each individual's fitness can be expressed relative to the average of their class, by $W_{\mathrm{sTU}}(\phi,\phi',\mu,\mu',\alpha,\beta) = w_{\mathrm{sTU}}(\phi,\phi',\mu,\mu',\alpha,\beta)/\ \bar{w}_{\mathrm{sTU}}(\alpha,\beta)$.

Gene fitness –There are 16 classes of gene, because every class of individual carries one maternal-origin gene and one paternal-origin gene. Thus, gene classes are denoted in the form sTUv, where $\mathrm{v} \in \{1,2\}$ according to whether the gene is of maternal (v = 1) or paternal (v = 2) origin. The fitness of a gene, defined in terms of its probability of being transmitted to a potential (rather than a realised) zygote, is proportional to the fitness of the individual who carries it. Consequently, the relative fitness of a gene is equal to the relative fitness of its carrier: $W_{\mathrm{sTU1}}(\phi,\phi',\mu,\mu',\alpha,\beta) = W_{\mathrm{sTU2}}(\phi,\phi',\mu,\mu',\alpha,\beta) = W_{\mathrm{sTU}}(\phi,\phi',\mu,\mu',\alpha,\beta)$.

Reproductive value – The flow of autosomal genes between classes is exactly the same under XY/XO and ZW inheritance, so the class reproductive values are identical to those derived in section 1.1.1, i.e. $c_{\mathrm{sTUv}} = 1/16$ for all gene classes.

PGE in females – Genes can be assigned genic values g according to their heritable tendency for any trait of interest. The condition for natural selection to favour an increase in this trait is $\mathrm{d}W/\mathrm{d}g > 0$, where $W = \sum_{\mathrm{s}\in\{\mathrm{f,m}\},\mathrm{T}\in\{\mathrm{M,P}\},\mathrm{U}\in\{\mathrm{M,P}\},\mathrm{v}\in\{1,2\}} c_{\mathrm{sTUv}}\ W_{\mathrm{sTUv}}$. Here, g is the genic value associated with an autosomal gene in a potential zygote. Thus, the condition for increase in female PGE is $\sum_{\mathrm{s}\in\{\mathrm{f,m}\},\mathrm{T}\in\{\mathrm{M,P}\},\mathrm{U}\in\{\mathrm{M,P}\},\mathrm{v}\in\{1,2\}} c_{\mathrm{sTUv}}$ $((\mathrm{d}W_{\mathrm{sTUv}}/\mathrm{d}\phi) \times (\mathrm{d}\phi/\mathrm{d}G) \times (\mathrm{d}G/\mathrm{d}g_{\mathrm{sTUv}}) + (\mathrm{d}W_{\mathrm{sTUv}}/\mathrm{d}\phi') \times (\mathrm{d}\phi'/\mathrm{d}G') \times (\mathrm{d}G'/\mathrm{d}g_{\mathrm{sTUv}})) > 0$, where G is the average genic value among those genes at the same locus whose expression controls PGE in the focal individual's mother, and G' is the average genic value among those genes at the same locus whose expression controls PGE in the females contributing offspring to the focal individual's mating group. Here: $\mathrm{d}\phi/\mathrm{d}G = \mathrm{d}\phi'/\mathrm{d}G' = 1$; $\mathrm{d}G/\mathrm{d}g_{\mathrm{sTUv}} = p_{\mathrm{sTUv}}$ is the consanguinity between the focal class-sTUv gene and the genes, residing at the same locus, who control PGE in the mother of the focal gene's carrier; and $\mathrm{d}G'/\mathrm{d}g_{\mathrm{sTUv}} = p'_{\mathrm{sTUv}}$ is the consanguinity between the focal class-sTUv gene and the genes, residing at the same locus, who control PGE in the females who contribute offspring to the focal individual's mating group. Note that, because $\mathrm{d}W_{\mathrm{fTUv}}/\mathrm{d}\phi' = 0$ for all classes of genes in females, we need only calculate consanguinities p'_{mTUv} for classes of genes in males. Again, the consanguinities will

depend upon which class or classes of genes control PGE in females -- see the Consanguinity section below.

PGE in males – Similarly, the condition for increase in male PGE is $\sum_{s\in\{f,m\},T\in\{M,P\},U\in\{M,P\},v\in\{1,2\}} c_{sTUv}\ ((dW_{sTUv}/d\mu) \times (d\mu/dG) \times (dG/dg_{sTUv})) > 0$, where G is the average genic value among those genes at the same locus whose expression controls PGE in the focal individual's father. Here, $d\mu/dG = 1$, and $dG/dg_{sTUv} = q_{sTUv}$ is the consanguinity between the focal class-sTUv gene and the genes, residing at the same locus, who control PGE in the father of the focal gene's carrier. Again, the consanguinities will depend upon which class or classes of genes control PGE in males -- see the Consanguinity section below.

Consanguinity – The inheritance of autosomal genes is exactly the same under XY/XO and ZW inheritance so, just as in section 1.1: the consanguinity of mating partners is $\rho = a/(8\text{-}7a)$. Moreover, as before, we have $\rho_{M-} = a((1/2) \times (1+\rho)/2 + (1/2) \times \rho)$, $\rho_{P-} = a\ \rho$, $\rho_{-M} = a((1/2) \times (1+\rho)/2 + (1/2) \times \rho)$, $\rho_{-P} = a\ \rho$, $\rho_{MM} = a\ (1+\rho)/2$, $\rho_{MP} = a\ \rho$, $\rho_{PM} = a\ \rho$ and $\rho_{PP} = a\ \rho$. These coefficients define all the consanguinities needed to solve the model (listed in Tables A3.1.1 & A3.1.2).

Potential for PGE – The potential for female PGE and the potential for male PGE are calculated in the same way as before, and these are illustrated in Figure 3 of the main text.

3.2. Z-linked genes

Classes – There are 4 classes of individual, classified according to their sex s ∈ {f,m} and the grandparent of origin of their paternal-origin gene U ∈ {M,P}. If the individual is female, she has no maternal-origin Z-linked genes. And if the individual is male, then all of his maternal-origin Z-linked genes are derived from his maternal grandfather. Thus, each class is notated in the form sTU, where T ∈ {M,-}, i.e. f-M, f-P, mPM and mPP.

Survival – The probabilities of survival for each class are: $S_{f\text{-}M}(\phi,\mu,\alpha,\beta) = (1/4)(1\text{-}\phi)(1\text{-}\mu) + (1/2)\phi(1\text{-}\mu)(1\text{-}\alpha) + (1/2)(1\text{-}\phi)\mu(1\text{-}\beta) + \phi\ \mu\ (1\text{-}\alpha)(1\text{-}\beta)$; $S_{f\text{-}P}(\phi,\mu,\alpha,\beta) = (1/4)(1\text{-}\phi)(1\text{-}\mu) + (1/2)\phi(1\text{-}\mu)(1\text{-}\alpha)$; $S_{mPM}(\phi,\mu,\alpha,\beta) = (1/4)(1\text{-}\phi)(1\text{-}\mu) + (1/2)(1\text{-}\phi)\mu(1\text{-}\beta)$; and $S_{mPP}(\phi,\mu,\alpha,\beta) = (1/4)(1\text{-}\phi)(1\text{-}\mu)$.

Reproductive success – Contingent upon survival, expected reproductive success is $R_{fTU}(\phi',\mu',\alpha,\beta) = 1$ for females and $R_{mTU}(\phi',\mu',\alpha,\beta) = (1\text{-}z(\phi',\mu',\alpha,\beta))/z(\phi',\mu',\alpha,\beta)$ for males, where $z(\phi',\mu',\alpha,\beta) = (S_{mPM}(\phi',\mu',\alpha,\beta) + S_{mPP}(\phi',\mu',\alpha,\beta))/(S_{f\text{-}M}(\phi',\mu',\alpha,\beta) + S_{f\text{-}P}(\phi',\mu',\alpha,\beta) + S_{mPM}(\phi',\mu',\alpha,\beta) + S_{mPP}(\phi',\mu',\alpha,\beta))$.

Fitness – Expected fitness is given by the product of survival to maturity and expected reproductive success contingent upon surviving to maturity, i.e. $w_{sTU}(\phi,\phi',\mu,\mu',\alpha,\beta) = S_{sTU}(\phi,\mu,\alpha,\beta)\times R_{sTU}(\phi',\mu',\alpha,\beta)$. As before, because PGE is

Recipient Class	Actor Class<br>A	A_{Mat}	A_{Pat}	A_{Mot}	A_{Fat}
fMM1	$(1+\rho)/2$	1	ρ	$(1+\rho)/2$	ρ
fMM2	ρ_{-M}	ρ_{MM}	ρ_{PM}	$a(1+\rho)/2$	$a\,\rho$
fMP1	$(1+\rho)/2$	1	ρ	$(1+\rho)/2$	ρ
fMP2	ρ_{-P}	ρ_{MP}	ρ_{PP}	$a\,\rho$	$a\,\rho$
fPM1	$(1+\rho)/2$	ρ	1	ρ	$(1+\rho)/2$
fPM2	ρ_{-M}	ρ_{MM}	ρ_{PM}	$a(1+\rho)/2$	$a\,\rho$
fPP1	$(1+\rho)/2$	ρ	1	ρ	$(1+\rho)/2$
fPP2	ρ_{-P}	ρ_{MP}	ρ_{PP}	$a\,\rho$	$a\,\rho$
mMM1	$(1+\rho)/2$	1	ρ	$(1+\rho)/2$	ρ
mMM2	ρ_{-M}	ρ_{MM}	ρ_{PM}	$a(1+\rho)/2$	$a\,\rho$
mMP1	$(1+\rho)/2$	1	ρ	$(1+\rho)/2$	ρ
mMP2	ρ_{-P}	ρ_{MP}	ρ_{PP}	$a\,\rho$	$a\,\rho$
mPM1	$(1+\rho)/2$	ρ	1	ρ	$(1+\rho)/2$
mPM2	ρ_{-M}	ρ_{MM}	ρ_{PM}	$a(1+\rho)/2$	$a\,\rho$
mPP1	$(1+\rho)/2$	ρ	1	ρ	$(1+\rho)/2$
mPP2	ρ_{-P}	ρ_{MP}	ρ_{PP}	$a\,\rho$	$a\,\rho$
mMM1′	$a(1+\rho)/2$	a	$a\,\rho$	$a(1+\rho)/2$	$a\,\rho$
mMM2′	$a\rho_{-M}$	$a\,\rho_{MM}$	$a\,\rho_{PM}$	$a^2(1+\rho)/2$	$a^2\,\rho$
mMP1′	$a(1+\rho)/2$	a	$a\,\rho$	$a(1+\rho)/2$	$a\,\rho$
mMP2′	$a\rho_{-P}$	$a\,\rho_{MP}$	$a\,\rho_{PP}$	$a^2\,\rho$	$a^2\,\rho$
mPM1′	$a(1+\rho)/2$	$a\,\rho$	a	$a\,\rho$	$a(1+\rho)/2$
mPM2′	$a\rho_{-M}$	$a\,\rho_{MM}$	$a\,\rho_{PM}$	$a^2(1+\rho)/2$	$a^2\,\rho$
mPP1′	$a(1+\rho)/2$	$a\,\rho$	a	$a\,\rho$	$a(1+\rho)/2$
mPP2′	$a\rho_{-P}$	$a\,\rho_{MP}$	$a\,\rho_{PP}$	$a^2\,\rho$	$a^2\,\rho$

Table A3.1.1. Consanguinities for the female PGE invasion analysis: autosomal genes. The five autosomal actor classes are the female's autosomal genes (A), her maternal-origin autosomal genes (A_{Mat}), her paternal-origin autosomal genes (A_{Pat}), her mother's autosomal genes (A_{Mot}) and her father's autosomal genes (A_{Fat}). Shown here are their consanguinities p_{sTUv} to each of the recipient classes sTUv among the focal female's offspring, and the consanguinities p'_{mTUv} to each of the recipient classes mTUv′, among the males competing for mates with the focal female's sons.

fitness can be expressed relative to the average of their class, by $W_{sTU}(\phi,\phi',\mu,\mu',\alpha,\beta) = w_{sTU}(\phi,\phi',\mu,\mu',\alpha,\beta)/\ \bar{w}_{sTU}(\alpha,\beta)$.

vanishingly rare in the population, the average fitness among all individuals of a particular class is given by $\bar{w}_{sTU}(\alpha,\beta) = w_{sTU}(0,0,0,0,\alpha,\beta)$, and each individual's

Gene fitness –There are 6 classes of gene, because individuals of each of the two female classes carry only paternal-origin genes, whereas individuals of each of the two male classes carry separate maternal-origin and paternal-origin genes. As before, gene classes are denoted in the form sTUv, where $v \in \{1,2\}$ according to

Recipient Class	Actor Class A	A_{Mat}	A_{Pat}	A_{Mot}	A_{Fat}
fMM1	ρ_{M-}	ρ_{MM}	ρ_{MP}	$a(1+\rho)/2$	$a\,\rho$
fMM2	$(1+\rho)/2$	1	ρ	$(1+\rho)/2$	ρ
fMP1	ρ_{M-}	ρ_{MM}	ρ_{MP}	$a(1+\rho)/2$	$a\,\rho$
fMP2	$(1+\rho)/2$	ρ	1	ρ	$(1+\rho)/2$
fPM1	ρ_{P-}	ρ_{PM}	ρ_{PP}	$a\,\rho$	$a\,\rho$
fPM2	$(1+\rho)/2$	1	ρ	$(1+\rho)/2$	ρ
fPP1	ρ_{P-}	ρ_{PM}	ρ_{PP}	$a\,\rho$	$a\,\rho$
fPP2	$(1+\rho)/2$	ρ	1	ρ	$(1+\rho)/2$
mMM1	ρ_{M-}	ρ_{MM}	ρ_{MP}	$a(1+\rho)/2$	$a\,\rho$
mMM2	$(1+\rho)/2$	1	ρ	$(1+\rho)/2$	ρ
mMP1	ρ_{M-}	ρ_{MM}	ρ_{MP}	$a(1+\rho)/2$	$a\,\rho$
mMP2	$(1+\rho)/2$	ρ	1	ρ	$(1+\rho)/2$
mPM1	ρ_{P-}	ρ_{PM}	ρ_{PP}	$a\,\rho$	$a\,\rho$
mPM2	$(1+\rho)/2$	1	ρ	$(1+\rho)/2$	ρ
mPP1	ρ_{P-}	ρ_{PM}	ρ_{PP}	$a\,\rho$	$a\,\rho$
mPP2	$(1+\rho)/2$	ρ	1	ρ	$(1+\rho)/2$

Table A3.1.2. Consanguinities for the male PGE analysis: autosomal genes. The five autosomal actor classes are the male's autosomal genes (A), his maternal-origin autosomal genes (A_{Mat}), his paternal-origin autosomal genes (A_{Pat}), his mother's autosomal genes (A_{Mot}) and his father's autosomal genes (A_{Fat}). Shown here are their consanguinities q_{sTUv} to each of the recipient classes sTUv among the male's offspring.

whether the gene is of maternal (v = 1) or paternal (v = 2) origin. The relative fitness of a gene in a female is given by $W_{f\text{-}U2}(\phi,\phi',\mu,\mu',\alpha,\beta) = W_{f\text{-}U}(\phi,\phi',\mu,\mu',\alpha,\beta)$ and the relative fitness of a gene in a male is given by $W_{mPU1}(\phi,\phi',\mu,\mu',\alpha,\beta) = W_{mPU2}(\phi,\phi',\mu,\mu',\alpha,\beta) = W_{mPU}(\phi,\phi',\mu,\mu',\alpha,\beta)$.

Reproductive value – As before, each gene's class in any generation may provide some information and some uncertainty about that gene's origin in the previous generation. For example, genes of class f-P2 in the present generation are derived from genes of class mPU2 in the previous generation, where U = M or P with equal probability. Consequently, the f-P2 gene derived from a mPM2 gene with probability $\wp_{f\text{-}P2\leftarrow mPM2} = ½$ and from a mPP2 gene with probability $\wp_{f\text{-}P2\leftarrow mPP2} = ½$. The corresponding system of 6 linear equations may be written in linear algebraic form as $\mathbf{c} = \mathbf{c}\bullet\mathbf{G}$, where $\mathbf{c} = \{c_{f\text{-}M2},c_{f\text{-}P2},c_{mPM1},c_{mPM2},c_{mPP1},c_{mPP2}\}$ is the vector of class reproductive values and $\mathbf{G}$ is the gene-flow matrix. Solving obtains $c_{sTUv} = 1/6$ for all gene classes.

PGE in females – Following the same procedure as before, the condition for increase in female PGE is $c_{f\text{-}M2}$ $((\mathrm{d}W_{f\text{-}M2}/\mathrm{d}\phi) \times (\mathrm{d}\phi/\mathrm{d}G) \times (\mathrm{d}G/\mathrm{d}g_{f\text{-}M2})) + c_{f\text{-}P2}$ $((\mathrm{d}W_{f\text{-}P2}/\mathrm{d}\phi) \times (\mathrm{d}\phi/\mathrm{d}G) \times (\mathrm{d}G/\mathrm{d}g_{f\text{-}P2})) + c_{mPM1}$ $((\mathrm{d}W_{mPM1}/\mathrm{d}\phi) \times (\mathrm{d}\phi/\mathrm{d}G) \times (\mathrm{d}G/\mathrm{d}g_{mPM1}) +$

$(\mathrm{d}W_{\mathrm{mPM1}}/\mathrm{d}\phi') \times (\mathrm{d}\phi'/\mathrm{d}G') \times (\mathrm{d}G'/\mathrm{d}g_{\mathrm{mPM1}})) + c_{\mathrm{mPM2}}\ ((\mathrm{d}W_{\mathrm{mPM2}}/\mathrm{d}\phi) \times (\mathrm{d}\phi/\mathrm{d}G) \times (\mathrm{d}G/\mathrm{d}g_{\mathrm{mPM2}}) + (\mathrm{d}W_{\mathrm{mPM2}}/\mathrm{d}\phi') \times (\mathrm{d}\phi'/\mathrm{d}G') \times (\mathrm{d}G'/\mathrm{d}g_{\mathrm{mPM2}})) + c_{\mathrm{mPP1}}\ ((\mathrm{d}W_{\mathrm{mPP1}}/\mathrm{d}\phi) \times (\mathrm{d}\phi/\mathrm{d}G) \times (\mathrm{d}G/\mathrm{d}g_{\mathrm{mPP1}}) + (\mathrm{d}W_{\mathrm{mPP1}}/\mathrm{d}\phi') \times (\mathrm{d}\phi'/\mathrm{d}G') \times (\mathrm{d}G'/\mathrm{d}g_{\mathrm{mPP1}})) + c_{\mathrm{mPP2}}\ ((\mathrm{d}W_{\mathrm{mPP2}}/\mathrm{d}\phi) \times (\mathrm{d}\phi/\mathrm{d}G) \times (\mathrm{d}G/\mathrm{d}g_{\mathrm{mPP2}}) + (\mathrm{d}W_{\mathrm{mPP2}}/\mathrm{d}\phi') \times (\mathrm{d}\phi'/\mathrm{d}G') \times (\mathrm{d}G'/\mathrm{d}g_{\mathrm{mPP2}})) > 0$, where: $\mathrm{d}\phi/\mathrm{d}G = \mathrm{d}\phi'/\mathrm{d}G' = 1$; $\mathrm{d}G/\mathrm{d}g_{\mathrm{sTUv}} = p_{\mathrm{sTUv}}$ is the consanguinity between the focal class-sTUv gene and the genes, residing at the same locus, who control PGE in the mother of the focal gene's carrier; and $\mathrm{d}G'/\mathrm{d}g_{\mathrm{mTUv}} = p'_{\mathrm{mTUv}}$ is the consanguinity between the focal class-sTUv gene and the genes, residing at the same locus, who control PGE in the females who contribute offspring to the focal individual's mating group.

PGE in males – Similarly, the condition for increase in male PGE is $c_{\mathrm{f\text{-}M2}}\ ((\mathrm{d}W_{\mathrm{f\text{-}M2}}/\mathrm{d}\mu) \times (\mathrm{d}\mu/\mathrm{d}G) \times (\mathrm{d}G/\mathrm{d}g_{\mathrm{f\text{-}M2}})) + c_{\mathrm{f\text{-}P2}}\ ((\mathrm{d}W_{\mathrm{f\text{-}P2}}/\mathrm{d}\mu) \times (\mathrm{d}\mu/\mathrm{d}G) \times (\mathrm{d}G/\mathrm{d}g_{\mathrm{f\text{-}P2}})) + c_{\mathrm{mPM1}}\ ((\mathrm{d}W_{\mathrm{mPM1}}/\mathrm{d}\mu) \times (\mathrm{d}\phi/\mathrm{d}G) \times (\mathrm{d}G/\mathrm{d}g_{\mathrm{mPM1}})) + c_{\mathrm{mPM2}}\ ((\mathrm{d}W_{\mathrm{mPM2}}/\mathrm{d}\mu) \times (\mathrm{d}\mu/\mathrm{d}G) \times (\mathrm{d}G/\mathrm{d}g_{\mathrm{mPM2}})) + c_{\mathrm{mPP1}}\ ((\mathrm{d}W_{\mathrm{mPP1}}/\mathrm{d}\mu) \times (\mathrm{d}\mu/\mathrm{d}G) \times (\mathrm{d}G/\mathrm{d}g_{\mathrm{mPP1}})) + c_{\mathrm{mPP2}}\ ((\mathrm{d}W_{\mathrm{mPP2}}/\mathrm{d}\mu) \times (\mathrm{d}\mu/\mathrm{d}G) \times (\mathrm{d}G/\mathrm{d}g_{\mathrm{mPP2}})) > 0$, where $\mathrm{d}\mu/\mathrm{d}G = 1$, and $\mathrm{d}G/\mathrm{d}g_{\mathrm{sTUv}} = q_{\mathrm{sTUv}}$ is the consanguinity between the focal class-sTUv gene and the genes, residing at the same locus, who control PGE in the father of the focal gene's carrier.

Consanguinity – The coefficient of inbreeding for Z-linked genes is $\rho^{***} = a((1/2) \times \rho^{***} + (1/2) \times \psi^{***})$. That is: with probability a the male and female share the same mother; so with probability ½ we pick the paternal-origin gene from the female and the maternal-origin gene from the male and hence their consanguinity is simply the consanguinity of mating partners, ρ^{***}; and with probability ½ we pick the paternal-origin genes from both individuals, in which case their consanguinity is that of two males in the same mating group, or ψ^{***}. Note that $\psi^{***} = a((1/4) \times 1 + (1/2) \times \rho^{***} + (1/4) \times \psi^{***})$. That is, with probability a the two males share the same mother; so with probability ¼ we pick the maternal-origin genes from both males, so their consanguinity is simply the mother's consanguinity to herself, i.e. 1; with probability ½ we pick the maternal-origin gene from one male and the paternal-origin gene from the other, in which case their consanguinity is that between mating partners, ρ^{***}; and with probability ¼ we pick the paternal-origin genes from both males, so their consanguinity is that of two males in the same mating group, ψ^{***}. Simultaneously solving both equations obtains $\rho^{***} = a^2/(8-a(6+a))$ and $\psi^{***} = a(2-a)/(8-a(6+a))$. From these coefficients can be defined other consanguinities between mating partners: $\rho^{***}_{\mathrm{-M}} = a\,\rho^{***}$, from the perspective of the male's maternal-origin gene; and $\rho^{***}_{\mathrm{-P}} = a\,\psi^{***}$, from the perspective of the male's paternal-origin gene. These coefficients define all the consanguinities needed to solve the model (listed in Tables A3.2.1 & A3.2.2).

Potential for PGE – The potentials for female and male PGE are calculated as described above. These are illustrated in Figure 3 of the main text.

Recipient Class	Actor Class Z	Z_{Mot}	Z_{Fat}
f-M2	ρ^{***}_{-M}	a	$a\ \rho^{***}$
f-P2	ρ^{***}_{-P}	$a\ \rho^{***}$	$a\ \psi^{***}$
mPM1	1	ρ^{***}	$(1+\rho^{***})/2$
mPM2	ρ^{***}_{-M}	a	$a\ \rho^{***}$
mPP1	1	ρ^{***}	$(1+\rho^{***})/2$
mPP2	ρ^{***}_{-P}	$a\ \rho^{***}$	$a\ \psi^{***}$
mPM1′	a	$a\ \rho^{***}$	$a(1+\rho^{***})/2$
mPM2′	$a\ \rho^{***}_{-M}$	a^2	$a^2\ \rho^{***}$
mPP1′	a	$a\ \rho^{***}$	$a(1+\rho^{***})/2$
mPP2′	$a\ \rho^{***}_{-P}$	$a^2\ \rho^{***}$	$a^2\ \psi^{***}$

Table A3.2.1. Consanguinities for the female PGE invasion analysis: Z-linked genes. The three Z-linked actor classes are the female's Z-linked genes (Z), her mother's Z-linked genes (Z_{Mot}) and her father's Z-linked genes (Z_{Fat}). Shown here are their consanguinities p_{sTUv} to each of the recipient classes sTUv among the focal female's offspring, and the consanguinities p'_{mTUv} to each of the recipient classes mTUv′, among the males competing for mates with the focal female's sons.

Recipient Class	Actor Class Z	Z_{Mat}	Z_{Pat}	Z_{Mot}	Z_{Fat}
f-M2	$(1+\rho^{***})/2$	1	ρ^{***}	1	ρ^{***}
f-P2	$(1+\rho^{***})/2$	ρ^{***}	1	ρ^{***}	$(1+\rho^{***})/2$
mPM1	ρ^{***}	ρ^{***}_{-M}	ρ^{***}_{-P}	$a\ \rho^{***}$	$a\ \psi^{***}$
mPM2	$(1+\rho^{***})/2$	1	ρ^{***}	1	ρ^{***}
mPP1	ρ^{***}	ρ^{***}_{-M}	ρ^{***}_{-P}	$a\ \rho^{***}$	$a\ \psi^{***}$
mPP2	$(1+\rho^{***})/2$	ρ^{***}	1	ρ^{***}	$(1+\rho^{***})/2$

Table A3.2.2. Consanguinities for the male PGE invasion analysis: Z-linked genes. The five Z-linked actor classes are the male's Z-linked genes (Z), his maternal-origin Z-linked genes (Z_{Mat}), his paternal-origin Z-linked genes (Z_{Pat}), his mother's Z-linked genes (Z_{Mot}) and his father's Z-linked genes (Z_{Fat}). Shown here are their consanguinities q_{sTUv} to each of the recipient classes sTUv among the male's offspring.

3.3. W-linked genes

Classes – There are 2 classes of individual, classified according to their sex s $\in$ {f,m}. If the individual is female, then all of her W-linked genes are maternal in origin (and came from her maternal grandmother). And if the individual is male, he has no W-linked genes. Thus, class is notated in the form s $\in$ {f,m}.

Survival – The probabilities of survival for each class are $S_f(\phi,\mu,\alpha,\beta) = (1/2)(1-\phi)(1-\mu) + \phi(1-\mu)(1-\alpha) + (1/2)(1-\phi)\mu(1-\beta) + \phi\ \mu\ (1-\alpha)(1-\beta)$ and $S_m(\phi,\mu,\alpha,\beta) = (1/2)(1-\phi)(1-\mu) + (1/2)(1-\phi)\mu(1-\beta)$.

Reproductive success – Contingent upon survival, expected reproductive success is $R_f(\phi',\mu',\alpha,\beta) = 1$ for females and $R_m(\phi',\mu',\alpha,\beta) = (1-z(\phi',\mu',\alpha,\beta))/z(\phi',\mu',\alpha,\beta)$ for males, where $z(\phi',\mu',\alpha,\beta) = S_m(\phi',\mu',\alpha,\beta)/(S_f(\phi',\mu',\alpha,\beta) + S_m(\phi',\mu',\alpha,\beta))$.

Fitness – Expected fitness is given by the product of survival to maturity and expected reproductive success contingent upon surviving to maturity, i.e. $w_s(\phi,\phi',\mu,\mu',\alpha,\beta) = S_s(\phi,\mu,\alpha,\beta)\times R_s(\phi',\mu',\alpha,\beta)$. As before, because PGE is vanishingly rare in the population, the average fitness among all individuals of a particular class is given by $\bar{w}_s(\alpha,\beta) = w_s(0,0,0,0,\alpha,\beta)$, and each individual's fitness can be expressed relative to the average of their class, by $W_s(\phi,\phi',\mu,\mu',\alpha,\beta) = w_s(\phi,\phi',\mu,\mu',\alpha,\beta)/\ \bar{w}_s(\alpha,\beta)$.

Gene fitness – There is only one class of W-linked gene, because females carry only one class of W-linked gene and males carry no W-linked genes. Accordingly, the relative fitness of a W-linked gene in a female is simply $W_f(\phi,\phi',\mu,\mu',\alpha,\beta)$.

Reproductive value – Since there is only one class of W-linked gene, all reproductive value belongs to this class: $c_f = 1$.

PGE in females – Following the same procedure as before, the condition for increase in female PGE is $c_f\ ((\mathrm{d}W_f/\mathrm{d}\phi) \times (\mathrm{d}\phi/\mathrm{d}G) \times (\mathrm{d}G/\mathrm{d}g_f)) > 0$. Once again: $\mathrm{d}\phi/\mathrm{d}G$ is an arbitrary mapping between genic value and phenotypic value, and can be set to 1; and $\mathrm{d}G/\mathrm{d}g_f = p_f$ is the consanguinity between the focal gene and the genes, residing at the same locus, who control PGE in the mother of the focal gene's carrier.

PGE in males – Similarly, the condition for increase in male PGE is $c_f\ ((\mathrm{d}W_f/\mathrm{d}\mu) \times (\mathrm{d}\mu/\mathrm{d}G) \times (\mathrm{d}G/\mathrm{d}g_f)) > 0$, where $\mathrm{d}\mu/\mathrm{d}G = 1$ and $\mathrm{d}G/\mathrm{d}g_f = q_f$ is the consanguinity between the focal class-sTUv gene and the genes, residing at the same locus, who control PGE in the father of the focal gene's carrier.

Consanguinity – Coefficients of inbreeding are undefined for W-linked genes, because males do not carry any such genes. Only three consanguinities feature in the W-linkage analysis: between a female's W-genes and her mother's W-genes; between a female's W-genes and her maternal grandmother's W-genes; and between a female's W-genes and her paternal grandmother's W-genes (listed in Tables A3.3.1 & A3.3.2).

Potential for PGE – The potentials for female and male PGE are calculated as described above. These are illustrated in Figure 3 of the main text.

Recipient Class	Actor Class W	W_{Mot}
f	1	1

Table A3.3.1. Consanguinities for the female PGE invasion analysis: W-linked genes. The two W-linked actor classes are the female's own W-linked genes (W) and the female's mother's W-linked genes (W_{Mot}). Shown here are their consanguinity p_f to the single recipient class f among the focal female's daughters.

Recipient Class	Actor Class W_{Mot}
f	a

Table A3.3.2. Consanguinities for the male PGE invasion analysis: W-linked genes. The only W-linked actor class is the male's mother's W-linked genes (W_{Mot}). Shown here are their consanguinity q_f to the recipient class f among the focal male's daughters.

4. MGE in females and/or males under ZW inheritance: invasion analysis

4.1. Autosomal genes

Classes – There are 8 classes of individual, classified according to their sex s $\in$ {f,m}, the grandparent of origin of their maternal-origin gene T $\in$ {M,P} and the grandparent of origin of their paternal-origin gene U $\in$ {M,P}. Each class is notated in the form sTU, i.e. fMM, fMP, fPM, fPP, mMM, mMP, mPM and mPP.

Survival – Denoting the probability that a zygote's mother exhibited MGE by ϕ, the probability that the zygote's father exhibited MGE by μ, the viability cost associated with female MGE by α and the viability cost associated with male MGE by β, the probability that each potential zygote survives to maturity is: $S_{fMM}(\phi,\mu,\alpha,\beta) = (1/8)(1-\phi)(1-\mu)$; $S_{fMP}(\phi,\mu,\alpha,\beta) = (1/8)(1-\phi)(1-\mu) + (1/4)(1-\phi)\mu(1-\beta)$; $S_{fPM}(\phi,\mu,\alpha,\beta) = (1/8)(1-\phi)(1-\mu)$; $S_{fPP}(\phi,\mu,\alpha,\beta) = (1/8)(1-\phi)(1-\mu) + (1/4)(1-\phi)\mu(1-\beta)$; $S_{mMM}(\phi,\mu,\alpha,\beta) = (1/8)(1-\phi)(1-\mu)$; $S_{mMP}(\phi,\mu,\alpha,\beta) = (1/8)(1-\phi)(1-\mu) + (1/4)(1-\phi)\mu(1-\beta)$; $S_{mPM}(\phi,\mu,\alpha,\beta) = (1/8)(1-\phi)(1-\mu) + (1/2)\phi(1-\mu)(1-\alpha)$; and $S_{mPP}(\phi,\mu,\alpha,\beta) = (1/8)(1-\phi)(1-\mu) + (1/2)\phi(1-\mu)(1-\alpha) + (1/4)(1-\phi)\mu(1-\beta) + \phi\ \mu\ (1-\alpha)(1-\beta)$.

Reproductive success – Upon survival to maturity, every female achieves a unit of expected reproductive success, and every male achieves $(1-z(\phi',\mu',\alpha,\beta))/z(\phi',\mu',\alpha,\beta)$ units of expected reproductive success, where: $z(\phi',\mu',\alpha,\beta) = (\sum_{T\in\{M,P\},U\in\{M,P\}} S_{mTU}(\phi',\mu',\alpha,\beta)) / (\sum_{s\in\{f,m\},T\in\{M,P\},U\in\{M,P\}} S_{sTU}(\phi',\mu',\alpha,\beta))$ is the sex ratio (proportion male) in his mating group; ϕ' is the level of MGE among the females contributing offspring to the mating group; and μ' is the level of MGE among the fathers contributing offspring to the mating group. Thus, contingent upon survival, expected

reproductive success is $R_{fTU}(\phi',\mu',\alpha,\beta) = 1$ for females and $R_{mTU}(\phi',\mu',\alpha,\beta) = (1-z(\phi',\mu',\alpha,\beta))/z(\phi',\mu',\alpha,\beta)$ for males.

Fitness – Expected fitness is given by the product of survival to maturity and expected reproductive success contingent upon surviving to maturity, i.e. $w_{sTU}(\phi,\phi',\mu,\mu',\alpha,\beta) = S_{sTU}(\phi,\mu,\alpha,\beta)\times R_{sTU}(\phi',\mu',\alpha,\beta)$. Because MGE is vanishingly rare in the population, the average fitness among all individuals of a particular class is given by $\bar{w}_{sTU}(\alpha,\beta) = w_{sTU}(0,0,0,0,\alpha,\beta)$, and each individual's fitness can be expressed relative to the average of their class, by $W_{sTU}(\phi,\phi',\mu,\mu',\alpha,\beta) = w_{sTU}(\phi,\phi',\mu,\mu',\alpha,\beta)/\ \bar{w}_{sTU}(\alpha,\beta)$.

Gene fitness –There are 16 classes of gene, because every class of individual carries one maternal-origin gene and one paternal-origin gene. Thus, gene classes are denoted in the form sTUv, where $v \in \{1,2\}$ according to whether the gene is of maternal (v = 1) or paternal (v = 2) origin. The fitness of a gene, defined in terms of its probability of being transmitted to a potential (rather than a realised) zygote, is proportional to the fitness of the individual who carries it. Consequently, the relative fitness of a gene is equal to the relative fitness of its carrier: $W_{sTU1}(\phi,\phi',\mu,\mu',\alpha,\beta) = W_{sTU2}(\phi,\phi',\mu,\mu',\alpha,\beta) = W_{sTU}(\phi,\phi',\mu,\mu',\alpha,\beta)$.

Reproductive value – The flow of autosomal genes between classes is exactly the same under XY/XO and ZW inheritance, so the class reproductive values are identical to those derived in section 2.1.1, i.e. $c_{sTUv} = 1/16$ for all gene classes.

MGE in females – The condition for increase in female MGE is $\sum_{s\in\{f,m\},T\in\{M,P\},U\in\{M,P\},v\in\{1,2\}} c_{sTUv}\ ((\mathrm{d}W_{sTUv}/\mathrm{d}\phi) \times (\mathrm{d}\phi/\mathrm{d}G) \times (\mathrm{d}G/\mathrm{d}g_{sTUv}) + (\mathrm{d}W_{sTUv}/\mathrm{d}\phi') \times (\mathrm{d}\phi'/\mathrm{d}G') \times (\mathrm{d}G'/\mathrm{d}g_{sTUv})) > 0$, where G is the average genic value among those genes at the same locus whose expression controls MGE in the focal individual's mother, and G' is the average genic value among those genes at the same locus whose expression controls MGE in the females contributing offspring to the focal individual's mating group. Here: $\mathrm{d}\phi/\mathrm{d}G = \mathrm{d}\phi'/\mathrm{d}G' = 1$; $\mathrm{d}G/\mathrm{d}g_{sTUv} = p_{sTUv}$ is the consanguinity between the focal class-sTUv gene and the genes, residing at the same locus, who control MGE in the mother of the focal gene's carrier; and $\mathrm{d}G'/\mathrm{d}g_{sTUv} = p'_{sTUv}$ is the consanguinity between the focal class-sTUv gene and the genes, residing at the same locus, who control MGE in the females who contribute offspring to the focal individual's mating group. Note that, because $\mathrm{d}W_{fTUv}/\mathrm{d}\phi' = 0$ for all classes of genes in females, we need only calculate consanguinities p'_{mTUv} for classes of genes in males. Again, the consanguinities will depend upon which class or classes of genes control MGE in females -- see the Consanguinity section below.

MGE in males – Similarly, the condition for increase in male MGE is $\sum_{s\in\{f,m\},T\in\{M,P\},U\in\{M,P\},v\in\{1,2\}} c_{sTUv}\ ((\mathrm{d}W_{sTUv}/\mathrm{d}\mu) \times (\mathrm{d}\mu/\mathrm{d}G) \times (\mathrm{d}G/\mathrm{d}g_{sTUv})) > 0$, where G is the average genic value among those genes at the same locus whose expression controls MGE in the focal individual's father. Here, $\mathrm{d}\mu/\mathrm{d}G = 1$, and $\mathrm{d}G/\mathrm{d}g_{sTUv} = q_{sTUv}$ is the consanguinity between the focal class-sTUv gene and the genes, residing at the same

locus, who control MGE in the father of the focal gene's carrier. Again, the consanguinities will depend upon which class or classes of genes control MGE in males -- see the Consanguinity section below.

Consanguinity – The inheritance of autosomal genes is exactly the same under XY/XO and ZW inheritance so, just as in section 2.1: the consanguinity of mating partners is $\rho = a/(8\text{-}7a)$. Moreover, as before, we have $\rho_{M-} = a((1/2) \times (1+\rho)/2 + (1/2) \times \rho)$, $\rho_{P-} = a\,\rho$, $\rho_{-M} = a((1/2) \times (1+\rho)/2 + (1/2) \times \rho)$, $\rho_{-P} = a\,\rho$, $\rho_{MM} = a\,(1+\rho)/2$, $\rho_{MP} = a\,\rho$, $\rho_{PM} = a\,\rho$ and $\rho_{PP} = a\,\rho$. These coefficients define all the consanguinities needed to solve the model (listed in Tables A3.1.1 & A3.1.2).

Potential for MGE – The potential for female MGE and the potential for male MGE are calculated in the same way as before, and these are illustrated in Figure 3 of the main text.

4.2. Z-linked genes

Classes – There are 4 classes of individual, classified according to their sex s ∈ {f,m} and the grandparent of origin of their paternal-origin gene U ∈ {M,P}. If the individual is female, she has no maternal-origin Z-linked genes. And if the individual is male, then all of his maternal-origin Z-linked genes are derived from his maternal grandfather. Thus, each class is notated in the form sTU, where T ∈ {M,-}, i.e. f-M, f-P, mPM and mPP.

Survival – The probabilities of survival for each class are: $S_{\text{f-M}}(\phi,\mu,\alpha,\beta) = (1/4)(1\text{-}\phi)(1\text{-}\mu)$; $S_{\text{f-P}}(\phi,\mu,\alpha,\beta) = (1/4)(1\text{-}\phi)(1\text{-}\mu) + (1/2)(1\text{-}\phi)\mu(1\text{-}\beta)$; $S_{\text{mPM}}(\phi,\mu,\alpha,\beta) = (1/4)(1\text{-}\phi)(1\text{-}\mu) + (1/2)\phi(1\text{-}\mu)(1\text{-}\alpha)$; and $S_{\text{mPP}}(\phi,\mu,\alpha,\beta) = (1/4)(1\text{-}\phi)(1\text{-}\mu) + (1/2)\phi(1\text{-}\mu)(1\text{-}\alpha) + (1/2)(1\text{-}\phi)\mu(1\text{-}\beta) + \phi\mu(1\text{-}\alpha)(1\text{-}\beta)$.

Reproductive success – Contingent upon survival, expected reproductive success is $R_{\text{fTU}}(\phi',\mu',\alpha,\beta) = 1$ for females and $R_{\text{mTU}}(\phi',\mu',\alpha,\beta) = (1\text{-}z(\phi',\mu',\alpha,\beta))/z(\phi',\mu',\alpha,\beta)$ for males, where $z(\phi',\mu',\alpha,\beta) = (S_{\text{mPM}}(\phi',\mu',\alpha,\beta) + S_{\text{mPP}}(\phi',\mu',\alpha,\beta))/(S_{\text{f-M}}(\phi',\mu',\alpha,\beta) + S_{\text{f-P}}(\phi',\mu',\alpha,\beta) + S_{\text{mPM}}(\phi',\mu',\alpha,\beta) + S_{\text{mPP}}(\phi',\mu',\alpha,\beta))$.

Fitness – Expected fitness is given by the product of survival to maturity and expected reproductive success contingent upon surviving to maturity, i.e. $w_{\text{sTU}}(\phi,\phi',\mu,\mu',\alpha,\beta) = S_{\text{sTU}}(\phi,\mu,\alpha,\beta) \times R_{\text{sTU}}(\phi',\mu',\alpha,\beta)$. As before, because MGE is vanishingly rare in the population, the average fitness among all individuals of a particular class is given by $\bar{w}_{\text{sTU}}(\alpha,\beta) = w_{\text{sTU}}(0,0,0,0,\alpha,\beta)$, and each individual's fitness can be expressed relative to the average of their class, by $W_{\text{sTU}}(\phi,\phi',\mu,\mu',\alpha,\beta) = w_{\text{sTU}}(\phi,\phi',\mu,\mu',\alpha,\beta)/\ \bar{w}_{\text{sTU}}(\alpha,\beta)$.

Gene fitness –There are 6 classes of gene, because individuals of each of the two female classes carry only paternal-origin genes, whereas individuals of each of the two male classes carry separate maternal-origin and paternal-origin genes. As

before, gene classes are denoted in the form sTUv, where v ∈ {1,2} according to whether the gene is of maternal (v = 1) or paternal (v = 2) origin. The relative fitness of a gene in a female is given by $W_{\text{f-U2}}(\phi,\phi',\mu,\mu',\alpha,\beta) = W_{\text{f-U}}(\phi,\phi',\mu,\mu',\alpha,\beta)$ and the relative fitness of a gene in a male is given by $W_{\text{mPU1}}(\phi,\phi',\mu,\mu',\alpha,\beta) = W_{\text{mPU2}}(\phi,\phi',\mu,\mu',\alpha,\beta) = W_{\text{mPU}}(\phi,\phi',\mu,\mu',\alpha,\beta)$.

Reproductive value – The flow of Z-linked genes between classes is exactly the same as in section 3.2. Accordingly, $c_{\text{sTUv}} = 1/6$ for all gene classes.

MGE in females – Following the same procedure as before, the condition for increase in female MGE is $c_{\text{f-M2}}$ ((d$W_{\text{f-M2}}$/dϕ) × (dϕ/dG) × (dG/d$g_{\text{f-M2}}$)) + $c_{\text{f-P2}}$ ((d$W_{\text{f-P2}}$/dϕ) × (dϕ/dG) × (dG/d$g_{\text{f-P2}}$)) + c_{mPM1} ((dW_{mPM1}/dϕ) × (dϕ/dG) × (dG/dg_{mPM1}) + (dW_{mPM1}/dϕ′) × (dϕ′/dG') × (dG'/dg_{mPM1})) + c_{mPM2} ((dW_{mPM2}/dϕ) × (dϕ/dG) × (dG/dg_{mPM2}) + (dW_{mPM2}/dϕ′) × (dϕ′/dG') × (dG'/dg_{mPM2})) + c_{mPP1} ((dW_{mPP1}/dϕ) × (dϕ/dG) × (dG/dg_{mPP1}) + (dW_{mPP1}/dϕ′) × (dϕ′/dG') × (dG'/dg_{mPP1})) + c_{mPP2} ((dW_{mPP2}/dϕ) × (dϕ/dG) × (dG/dg_{mPP2}) + (dW_{mPP2}/dϕ′) × (dϕ′/dG') × (dG'/dg_{mPP2})) > 0, where: dϕ/dG = dϕ′/dG' = 1; dG/dg_{sTUv} = p_{sTUv} is the consanguinity between the focal class-sTUv gene and the genes, residing at the same locus, who control MGE in the mother of the focal gene's carrier; and dG'/dg_{mTUv} = p'_{mTUv} is the consanguinity between the focal class-sTUv gene and the genes, residing at the same locus, who control MGE in the females who contribute offspring to the focal individual's mating group.

MGE in males – Similarly, the condition for increase in male MGE is $c_{\text{f-M2}}$ ((d$W_{\text{f-M2}}$/dμ) × (dμ/dG) × (dG/d$g_{\text{f-M2}}$)) + $c_{\text{f-P2}}$ ((d$W_{\text{f-P2}}$/dμ) × (dμ/dG) × (dG/d$g_{\text{f-P2}}$)) + c_{mPM1} ((dW_{mPM1}/dμ) × (dϕ/dG) × (dG/dg_{mPM1})) + c_{mPM2} ((dW_{mPM2}/dμ) × (dμ/dG) × (dG/dg_{mPM2})) + c_{mPP1} ((dW_{mPP1}/dμ) × (dμ/dG) × (dG/dg_{mPP1})) + c_{mPP2} ((dW_{mPP2}/dμ) × (dμ/dG) × (dG/dg_{mPP2})) > 0, where dμ/dG = 1, and dG/dg_{sTUv} = q_{sTUv} is the consanguinity between the focal class-sTUv gene and the genes, residing at the same locus, who control MGE in the father of the focal gene's carrier.

Consanguinity – The coefficients of consanguinity are exactly the same as described in section 3.2 (listed in Tables A3.2.1 & A3.2.2).

Potential for MGE – The potentials for female and male MGE are calculated as described above. These are illustrated in Figure 3 of the main text.

4.3. W-linked genes

Classes – There are 2 classes of individual, classified according to their sex s ∈ {f,m}. If the individual is female, then all of her W-linked genes are maternal in origin (and came from her maternal grandmother). And if the individual is male, he has no W-linked genes. Thus, class is notated in the form s ∈ {f,m}.

Survival – The probabilities of survival for each class are $S_f(\phi,\mu,\alpha,\beta) = (1/2)(1-\phi)(1-\mu) + (1/2)(1-\phi)\mu(1-\beta)$ and $S_m(\phi,\mu,\alpha,\beta) = (1/2)(1-\phi)(1-\mu) + \phi(1-\mu)(1-\alpha) + (1/2)(1-\phi)\mu(1-\beta) + \phi\mu(1-\alpha)(1-\beta)$.

Reproductive success – Contingent upon survival, expected reproductive success is $R_f(\phi',\mu',\alpha,\beta) = 1$ for females and $R_m(\phi',\mu',\alpha,\beta) = (1-z(\phi',\mu',\alpha,\beta))/z(\phi',\mu',\alpha,\beta)$ for males, where $z(\phi',\mu',\alpha,\beta) = S_m(\phi',\mu',\alpha,\beta)/(S_f(\phi',\mu',\alpha,\beta) + S_m(\phi',\mu',\alpha,\beta))$.

Fitness – Expected fitness is given by the product of survival to maturity and expected reproductive success contingent upon surviving to maturity, i.e. $w_s(\phi,\phi',\mu,\mu',\alpha,\beta) = S_s(\phi,\mu,\alpha,\beta)\times R_s(\phi',\mu',\alpha,\beta)$. As before, because MGE is vanishingly rare in the population, the average fitness among all individuals of a particular class is given by $\bar{w}_s(\alpha,\beta) = w_s(0,0,0,0,\alpha,\beta)$, and each individual's fitness can be expressed relative to the average of their class, by $W_s(\phi,\phi',\mu,\mu',\alpha,\beta) = w_s(\phi,\phi',\mu,\mu',\alpha,\beta)/\ \bar{w}_s(\alpha,\beta)$.

Gene fitness – There is only one class of W-linked gene, because females carry only one class of W-linked gene and males carry no W-linked genes. Accordingly, the relative fitness of a W-linked gene in a female is simply $W_f(\phi,\phi',\mu,\mu',\alpha,\beta)$.

Reproductive value – Since there is only one class of W-linked gene, all reproductive value belongs to this class: $c_f = 1$.

MGE in females – Following the same procedure as before, the condition for increase in female MGE is $c_f\ ((\mathrm{d}W_f/\mathrm{d}\phi) \times (\mathrm{d}\phi/\mathrm{d}G) \times (\mathrm{d}G/\mathrm{d}g_f)) > 0$. Once again: $\mathrm{d}\phi/\mathrm{d}G$ is an arbitrary mapping between genic value and phenotypic value, and can be set to 1; and $\mathrm{d}G/\mathrm{d}g_f = p_f$ is the consanguinity between the focal gene and the genes, residing at the same locus, who control MGE in the mother of the focal gene's carrier.

MGE in males – Similarly, the condition for increase in male MGE is $c_f\ ((\mathrm{d}W_f/\mathrm{d}\mu) \times (\mathrm{d}\mu/\mathrm{d}G) \times (\mathrm{d}G/\mathrm{d}g_f)) > 0$, where $\mathrm{d}\mu/\mathrm{d}G = 1$ and $\mathrm{d}G/\mathrm{d}g_f = q_f$ is the consanguinity between the focal class-sTUv gene and the genes, residing at the same locus, who control MGE in the father of the focal gene's carrier.

Consanguinity – The consanguinities are exactly the same as those derived in section 3.3 (listed in Tables A3.3.1 & A3.3.2).

Potential for MGE – The potentials for female and male MGE are calculated as described above. These are listed in illustrated in Figure 3 of the main text.

5. PGE in males under XY/XO inheritance: equilibrium analysis

5.1. Autosomal genes

Classes – There are 8 classes of individual, classified according to their sex s ∈ {f,m}, the grandparent of origin of their maternal-origin gene T ∈ {M,P} and the

grandparent of origin of their paternal-origin gene U ∈ {M,P}. Each class is notated in the form sTU, i.e. fMM, fMP, fPM, fPP, mMM, mMP, mPM and mPP.

Survival – The zygote survival functions are obtained by substituting $\phi = \beta = 0$ into those of section 1.1. This yields $S_{fMM}(\mu) = (1/8)(1-\mu) + (1/2)\mu$; $S_{fMP}(\mu) = (1/8)(1-\mu)$; $S_{fPM}(\mu) = (1/8)(1-\mu) + (1/2)\mu$; $S_{fPP}(\mu) = (1/8)(1-\mu)$; $S_{mMM}(\mu) = (1/8)(1-\mu)$; $S_{mMP}(\mu) = (1/8)(1-\mu)$; $S_{mPM}(\mu) = (1/8)(1-\mu)$; and $S_{mPP}(\mu) = (1/8)(1-\mu)$.

Reproductive success – Upon survival to maturity, every female achieves a unit of expected reproductive success, and every male achieves $(1-z(\mu'))/z(\mu')$ units of expected reproductive success, where: $z(\mu') = (\sum_{T\in\{M,P\},U\in\{M,P\}} S_{mTU}(\mu')) / (\sum_{s\in\{f,m\},T\in\{M,P\},U\in\{M,P\}} S_{sTU}(\mu'))$ is the sex ratio (proportion male) in his mating group and μ' is the level of PGE among the fathers contributing offspring to the mating group. Thus, contingent upon survival, expected reproductive success is $R_{fTU}(\mu') = 1$ for females and $R_{mTU}(\mu') = (1-z(\mu'))/z(\mu')$ for males.

Fitness – Expected fitness is given by the product of survival to maturity and expected reproductive success contingent upon surviving to maturity, i.e. $w_{sTU}(\mu,\mu') = S_{sTU}(\mu)\times R_{sTU}(\mu')$. The average fitness among all individuals of a particular class is given by $\bar{w}_{sTU}(\bar{\mu}) = w_{sTU}(\bar{\mu},\bar{\mu})$, and each individual's fitness can be expressed relative to the average of their class, by $W_{sTU}(\mu,\mu',\bar{\mu}) = w_{sTU}(\mu,\mu')/\bar{w}_{sTU}(\bar{\mu})$.

Gene fitness –There are 16 classes of gene, because every class of individual carries one maternal-origin gene and one paternal-origin gene. Thus, gene classes are denoted in the form sTUv, where v ∈ {1,2} according to whether the gene is of maternal (v = 1) or paternal (v = 2) origin. The fitness of a gene, defined in terms of its probability of being transmitted to a potential (rather than a realised) zygote, is proportional to the fitness of the individual who carries it. Consequently, the relative fitness of a gene is equal to the relative fitness of its carrier: $W_{sTU1}(\mu,\mu',\bar{\mu}) = W_{sTU2}(\mu,\mu',\bar{\mu}) = W_{sTU}(\mu,\mu',\bar{\mu})$.

Reproductive value – The flow of genes between classes determines each class's reproductive value. Each gene's class in any generation may provide some information and some uncertainty about that gene's origin in the previous generation. For example, genes of class fMM1 in the present generation are derived from genes of class fTU1 in the previous generation, where T = M or P and U = M or P. The effects of PGE in a male are accounted for in the survival of his potential offspring: for example, his potential male offspring are eliminated in the event that he undergoes PGE. So the class fTU1 donating genes to the class fMM1 involves T = M or P with equal probability, because there is no bias regarding the source of their maternal genes, but U = M or P with unequal probability, because there is possible bias regarding the source of their paternal genes. Specifically, U = M with probability $\bar{w}_{fTM1}/(\bar{w}_{fTM1} + \bar{w}_{fTP1}) = (1+3\bar{\mu})/(2+2\bar{\mu})$, and U = P with probability $\bar{w}_{fTP1}/(\bar{w}_{fTM1} + \bar{w}_{fTP1}) = (1-\bar{\mu})/(2+2\bar{\mu})$. Accordingly, $\wp_{fMM1\leftarrow fMM1} = (1+3\bar{\mu})/(4+4\bar{\mu})$, $\wp_{fMM1\leftarrow fPM1} = (1+3\bar{\mu})/(4+4\bar{\mu})$, $\wp_{fMM1\leftarrow fMP1} = (1-\bar{\mu})/(4+4\bar{\mu})$

and $\wp_{\text{fMM1}\leftarrow\text{fPP1}} = (1-\bar{\mu})/(4+4\bar{\mu})$. Note that, making the substitution $\bar{\mu} = 0$, all of these gene-flow coefficients are equal to ¼, as in section 1.1. Also note that, if the donor class involves genes in males, then there is no bias, because no male is the product of a PGE father (all PGE events lead to the production of daughters). This defines a system of 16 linear equations, which can be written as **c** = **c•G**, where **c** = $\{c_{\text{fMM1}}, c_{\text{fMM2}}, c_{\text{fMP1}}, \ldots, c_{\text{mPP2}}\}$ is the vector of class reproductive values and **G** is the gene-flow matrix. Solving (i.e. finding the left eigenvector of **G** corresponding to the eigenvalue 1) obtains $c_{\text{fTMv}} = (1+3\bar{\mu})/(16+8\bar{\mu})$, $c_{\text{fTPv}} = (1-\bar{\mu})/(16+8\bar{\mu})$, $c_{\text{mTU1}} = (1+\bar{\mu})/(16+8\bar{\mu})$ and $c_{\text{mTM2}} = (1-\bar{\mu})/(16+8\bar{\mu})$. In the absence of male PGE ($\bar{\mu} = 0$), all gene classes have reproductive value $c_{\text{sTUv}} = 1/16$; that is, a gene chosen at random from the distant future has an equal chance of tracing back to any of the 16 gene classes in the present generation. In the extreme of full male PGE ($\bar{\mu} = 1$), $c_{\text{fTMv}} = 1/6$, $c_{\text{fTPv}} = 0$, $c_{\text{mTU1}} = 1/12$ and $c_{\text{mTM2}} = 0$; that is, two third of the reproductive value of the population belongs to genes in females and only one third belongs to genes in males, just as under haplodiploidy.

PGE in males – The condition for increase in male PGE is $\sum_{s\in\{f,m\},T\in\{M,P\},U\in\{M,P\},v\in\{1,2\}} c_{\text{sTUv}} ((\text{d}W_{\text{sTUv}}/\text{d}\mu) \times (\text{d}\mu/\text{d}G) \times (\text{d}G/\text{d}g_{\text{sTUv}}) + (\text{d}W_{\text{sTUv}}/\text{d}\mu') \times (\text{d}\mu'/\text{d}G') \times (\text{d}G'/\text{d}g_{\text{sTUv}})) > 0$, where G is the average genic value among those genes at the same locus whose expression controls PGE in the focal individual's father, and G' is the average genic value among those genes at the same locus whose expression controls PGE in the males contributing offspring to the focal individual's mating group. Here: $\text{d}\mu/\text{d}G = \text{d}\mu'/\text{d}G' = 1$; $\text{d}G/\text{d}g_{\text{sTUv}} = q_{\text{sTUv}}$ is the consanguinity between the focal class-sTUv gene and the genes, residing at the same locus, who control PGE in the father of the focal gene's carrier; and $\text{d}G'/\text{d}g_{\text{sTUv}} = q'_{\text{sTUv}}$ is the consanguinity between the focal class-sTUv gene and the genes, residing at the same locus, who control PGE in the males who contribute offspring to the focal individual's mating group. Note that, because $\text{d}W_{\text{fTUv}}/\text{d}\mu' = 0$ for all classes of genes in females, we need only calculate consanguinities q'_{mTUv} for classes of genes in males. The consanguinities will depend upon which class or classes of genes control PGE in males -- see the Consanguinity section below, and Table A5.1.1, for details.

Consanguinity – The coefficient of inbreeding is $\rho = a((1/4)\times((1/2) + (1/2)\times(\theta\,\rho_{\text{-M}} + (1-\theta)\rho)) + (1/4)\times(\theta\,\rho_{\text{-M}} + (1-\theta)\rho) + (1/4)\times\rho + (1/4)\times(\theta\,\psi_{\text{M-}} + (1-\theta)\psi))$, where $\theta = 2\bar{\mu}/(1+\bar{\mu})$ is the probability that a focal adult female derived from an egg that was fertilized by a PGE sperm. Note that whilst the consanguinity of mating partners is ρ, the consanguinity of the genes that they transmit to their offspring is $\theta\,\rho_{\text{-M}} + (1-\theta)\rho$, because with probability θ the male exhibits PGE and so only transmits his maternal-origin genes, and with probability $1-\theta$ the male does not exhibit PGE and hence transmits maternal-origin and paternal-origin genes with equal probability. The conditional consanguinities of mating partners are: $\rho_{\text{-M}} = a((1/2)((1/2)+(1/2)(\theta\,\rho_{\text{-M}} + (1-\theta)\rho)) + (1/2)(\theta\,\rho_{\text{-M}} + (1-\theta)\rho))$, from the perspective of the male's maternal-origin gene; $\rho_{\text{M-}} = a((1/2)((1/2)+(1/2)(\theta\,\rho_{\text{-M}} + (1-\theta)\rho)) + (1/2)\rho)$, from the perspective of the female's maternal-origin gene; $\rho_{\text{-P}} = a((1/2)\rho + (1/2)(\theta\,\psi_{\text{M-}} + (1-$

$\theta)\psi)$), from the perspective of the male's paternal-origin gene; $\rho_{P-} = a((1/2)(\theta\,\rho_{-M} + (1-\theta)\rho) + (1/2)(\theta\,\psi_{M-} + (1-\theta)\psi))$, from the perspective of the female's paternal-origin gene; $\rho_{MM} = a((1/2) + (1/2)(\theta\,\rho_{-M} + (1-\theta)\rho))$, from the perspective of both mating partners' maternal-origin genes; $\rho_{MP} = a\rho$, from the perspective of the female's maternal-origin gene and the male's paternal-origin gene; $\rho_{PM} = a(\theta\,\rho_{-M} + (1-\theta)\rho)$, from the perspective of the female's paternal-origin gene and the male's maternal-origin gene; and $\rho_{PP} = a(\theta\,\psi_{M-} + (1-\theta)\psi)$, from the perspective of both mating partners' paternal-origin genes. The consanguinity of two males in the same mating group is $\psi = a((1/4)((1/2) + (1/2)\times(\theta\,\rho_{-M} + (1-\theta)\rho)) + (1/2)\rho + (1/4)\psi)$, and the conditional consanguinities between males are: $\psi_{M-} = a((1/2)((1/2) + (1/2)\times(\theta\,\rho_{-M} + (1-\theta)\rho)) + (1/2)\rho)$, from the perspective of one male's maternal-origin gene; $\psi_{P-} = a((1/2)\rho + (1/2)\psi)$, from the perspective of one male's paternal-origin gene; $\psi_{MM} = a((1/2) + (1/2)(\theta\,\rho_{-M} + (1-\theta)\rho))$, from the perspective of both males' maternal-origin genes; $\psi_{MP} = a\rho$, from the perspective of one male's maternal-origin gene and the other's paternal-origin gene; and $\psi_{PP} = a\psi$, from the perspective of both males' paternal-origin genes. The consanguinity of two females in the same mating group is $\zeta = a((1/4)((1/2) + (1/2)(\theta\,\rho_{-M} + (1-\theta)\rho)) + (1/2)(\theta\,\rho_{-M} + (1-\theta)\rho) + (1/4)(\theta^2\,\psi_{MM} + 2\theta(1-\theta)\psi_{M-} + (1-\theta)^2\psi))$, and the conditional consanguinities between females are: $\zeta_{M-} = a((1/2)((1/2) + (1/2)(\theta\,\rho_{-M} + (1-\theta)\rho)) + (1/2)(\theta\,\rho_{-M} + (1-\theta)\rho))$, from the perspective of one female's maternal-origin gene; $\zeta_{P-} = a((1/2)(\theta\,\rho_{-M} + (1-\theta)\rho) + (1/2)\,(\theta^2\,\psi_{MM} + 2\theta(1-\theta)\psi_{M-} + (1-\theta)^2\psi))$, from the perspective of one female's paternal-origin gene; $\zeta_{MM} = a((1/2) + (1/2)(\theta\,\rho_{-M} + (1-\theta)\rho))$, from the perspective of both females' maternal-origin genes; $\zeta_{MP} = a(\theta\,\rho_{-M} + (1-\theta)\rho)$, from the perspective of one female's maternal-origin gene and the other's paternal-origin gene; and $\zeta_{PP} = a(\theta^2\,\psi_{MM} + 2\theta(1-\theta)\psi_{M-} + (1-\theta)^2\psi)$, from the perspective of both females' paternal-origin genes. Simultaneously solving these equations yields $\rho = a(2+(2+a(2-a))\,\bar{\mu})/(16(1+\bar{\mu}) - a(14+(18-a(2+3a))\,\bar{\mu}))$, and each of the other consanguinities. And these in turn define all the consanguinities needed to solve the model (listed in Table A5.1.1).

Equilibrium level of PGE – If the condition for increase in PGE is not satisfied at $\bar{\mu} = 0$, then PGE cannot invade the population from rarity, and its equilibrium level is $\bar{\mu} = g^* = 0$. If the condition for increase in PGE is satisfied at $\bar{\mu} = 1$, then non-PGE cannot invade from rarity, and hence the equilibrium level of PGE is $g^* = 1$. Setting the LHS of the condition equal to zero, and solving for $\bar{\mu} = g^*$, obtains the intermediate equilibrium level of PGE. The equilibrium level of PGE is illustrated in Figure 4 of the main text.

5.2. X-linked genes

Classes – There are 4 classes of individual, classified according to their sex $s \in \{f,m\}$ and the grandparent of origin of their maternal-origin gene $T \in \{M,P\}$. If the individual is female, then all of her paternal-origin X-linked genes are derived from

Recipient Class	Actor Class A	A_{Mat}	A_{Pat}	A_{Mot}	A_{Fat}
fMM1	ρ_{M-}	ρ_{MM}	ρ_{MP}	$a\,\gamma$	$a\,\rho$
fMM2	$(1+\rho)/2$	1	ρ	$(1+\gamma)/2$	ρ
fMP1	ρ_{M-}	ρ_{MM}	ρ_{MP}	$a(1+\gamma)/2$	$a\,\rho$
fMP2	$(1+\rho)/2$	ρ	1	ρ	$(1+\rho)/2$
fPM1	ρ_{P-}	ρ_{PM}	ρ_{PP}	$a\,\gamma$	$a\,\eta$
fPM2	$(1+\rho)/2$	1	ρ	$(1+\gamma)/2$	ρ
fPP1	ρ_{P-}	ρ_{PM}	ρ_{PP}	$a\,\gamma$	$a\,\eta$
fPP2	$(1+\rho)/2$	ρ	1	ρ	$(1+\rho)/2$
mMM1	ρ_{M-}	ρ_{MM}	ρ_{MP}	$a(1+\gamma)/2$	$a\,\rho$
mMM2	$(1+\rho)/2$	1	ρ	$(1+\gamma)/2$	ρ
mMP1	ρ_{M-}	ρ_{MM}	ρ_{MP}	$a(1+\gamma)/2$	$a\,\rho$
mMP2	$(1+\rho)/2$	ρ	1	ρ	$(1+\rho)/2$
mPM1	ρ_{P-}	ρ_{PM}	ρ_{PP}	$a\,\gamma$	$a\,\eta$
mPM2	$(1+\rho)/2$	1	ρ	$(1+\gamma)/2$	ρ
mPP1	ρ_{P-}	ρ_{PM}	ρ_{PP}	$a\,\gamma$	$a\,\eta$
mPP2	$(1+\rho)/2$	ρ	1	ρ	$(1+\rho)/2$
mMM1′	$a\,\rho_{M-}$	$a\,\rho_{MM}$	$a\,\rho_{MP}$	$a^2(1+\gamma)/2$	$a^2\,\rho$
mMM2′	$a\,\psi_{M-}$	$a\,\psi_{MM}$	$a\,\psi_{MP}$	$a\,\psi_{MM}$	$a\,\psi_{MP}$
mMP1′	$a\,\rho_{M-}$	$a\,\rho_{MM}$	$a\,\rho_{MP}$	$a^2(1+\gamma)/2$	$a^2\,\rho$
mMP2′	$a\,\psi_{P-}$	$a\,\psi_{PM}$	$a\,\psi_{PP}$	$a\,\psi_{MP}$	$a\,\psi_{PP}$
mPM1′	$a\,\rho_{P-}$	$a\,\rho_{PM}$	$a\,\rho_{PP}$	$a\,\rho_{PM}$	$a^2\,\eta$
mPM2′	$a\,\psi_{M-}$	$a\,\psi_{MM}$	$a\,\psi_{MP}$	$a\,\psi_{MM}$	$a\,\psi_{MP}$
mPP1′	$a\,\rho_{P-}$	$a\,\rho_{PM}$	$a\,\rho_{PP}$	$a\,\rho_{PM}$	$a^2\,\eta$
mPP2′	$a\,\psi_{P-}$	$a\,\psi_{PM}$	$a\,\psi_{PP}$	$a\,\psi_{MP}$	$a\,\psi_{PP}$

Table A5.1.1. Consanguinities for the male PGE equilibrium analysis: autosomal genes. The five autosomal actor classes are the male's autosomal genes (A), his maternal-origin autosomal genes (A_{Mat}), his paternal-origin autosomal genes (A_{Pat}), his mother's autosomal genes (A_{Mot}) and his father's autosomal genes (A_{Fat}). Shown here are their consanguinities q_{sTUv} to each of the recipient classes sTUv among the male's offspring, and the consanguinities q'_{mTUv} to each of the recipient classes mTUv′, among the males competing for mates with the focal male's sons. Here, $\gamma = (\theta\,\rho_{-M} + (1-\theta)\rho)$ and $\eta = (\theta\,\psi_{-M} + (1-\theta)\psi)$.

her paternal grandmother. And if the individual is male, he has no paternal-origin X-linked genes. Thus, each class is notated in the form sTU, where U $\in$ {M,-}, i.e. fMM, fPM, mM- and mP-.

Survival – The probabilities of survival for each class are: $S_{fMM}(\phi,\mu,\alpha,\beta) = (1/4)(1-\mu) + (1/2)\mu$; $S_{fPM}(\phi,\mu,\alpha,\beta) = (1/4)(1-\mu) + (1/2)\mu$; $S_{mM-}(\phi,\mu,\alpha,\beta) = (1/4)(1-\mu)$; and $S_{mP-}(\phi,\mu,\alpha,\beta) = (1/4)(1-\mu)$.

Reproductive success – Contingent upon survival, expected reproductive success is $R_{\mathrm{fTU}}(\mu') = 1$ for females and $R_{\mathrm{mTU}}(\mu') = (1-z(\mu'))/z(\mu')$ for males, where $z(\mu') = (S_{\mathrm{mM-}}(\mu') + S_{\mathrm{mP-}}(\mu'))/(S_{\mathrm{fMM}}(\mu') + S_{\mathrm{fPM}}(\mu') + S_{\mathrm{mM-}}(\mu') + S_{\mathrm{mP-}}(\mu'))$.

Fitness – Expected fitness is given by the product of survival to maturity and expected reproductive success contingent upon surviving to maturity, i.e. $w_{\mathrm{sTU}}(\mu,\mu') = S_{\mathrm{sTU}}(\mu)\times R_{\mathrm{sTU}}(\mu')$. Average fitness among all individuals of a particular class is given by $\bar{w}_{\mathrm{sTU}}(\bar{\mu}) = w_{\mathrm{sTU}}(\bar{\mu}, \bar{\mu})$, and each individual's fitness can be expressed relative to the average of their class, by $W_{\mathrm{sTU}}(\mu,\mu', \bar{\mu}) = w_{\mathrm{sTU}}(\mu,\mu')/ \bar{w}_{\mathrm{sTU}}(\bar{\mu})$.

Gene fitness –There are 6 classes of gene, because individuals of each of the two female classes carry separate maternal-origin and paternal-origin genes, whereas individuals of each of the two male classes carry only maternal-origin genes. As before, gene classes are denoted in the form sTUv, where $v \in \{1,2\}$ according to whether the gene is of maternal (v = 1) or paternal (v = 2) origin. The relative fitness of a gene in a female is given by $W_{\mathrm{fTP1}}(\mu,\mu', \bar{\mu}) = W_{\mathrm{fTP2}}(\mu,\mu', \bar{\mu}) = W_{\mathrm{fTP}}(\mu,\mu', \bar{\mu})$ and the relative fitness of a gene in a male is given by $W_{\mathrm{mT-1}}(\mu,\mu', \bar{\mu}) = W_{\mathrm{mT-}}(\mu,\mu', \bar{\mu})$.

Reproductive value – PGE in males does not impact upon the gene-flow coefficients for X-linked genes, because males can only transmit maternal-origin X-linked genes anway. Accordingly, all of the non-zero gene-flow coefficients are equal to ½, and reproductive value is $c_{\mathrm{sTUv}} = 1/6$ for all gene classes, as in section A1.2.

PGE in males – As before, the condition for increase in male PGE is c_{fMM1} $((\mathrm{d}W_{\mathrm{fMM1}}/\mathrm{d}\mu) \times (\mathrm{d}\mu/\mathrm{d}G) \times (\mathrm{d}G/\mathrm{d}g_{\mathrm{fMM1}})) + c_{\mathrm{fMM2}}((\mathrm{d}W_{\mathrm{fMM2}}/\mathrm{d}\mu) \times (\mathrm{d}\mu/\mathrm{d}G) \times (\mathrm{d}G/\mathrm{d}g_{\mathrm{fMM2}})) + c_{\mathrm{fPM1}}((\mathrm{d}W_{\mathrm{fPM1}}/\mathrm{d}\mu) \times (\mathrm{d}\mu/\mathrm{d}G) \times (\mathrm{d}G/\mathrm{d}g_{\mathrm{fPM1}})) + c_{\mathrm{fPM2}}((\mathrm{d}W_{\mathrm{fPM2}}/\mathrm{d}\mu) \times (\mathrm{d}\mu/\mathrm{d}G) \times (\mathrm{d}G/\mathrm{d}g_{\mathrm{fPM2}})) + c_{\mathrm{mM-1}}((\mathrm{d}W_{\mathrm{mM-1}}/\mathrm{d}\mu) \times (\mathrm{d}\mu/\mathrm{d}G) \times (\mathrm{d}G/\mathrm{d}g_{\mathrm{mM-1}}) + (\mathrm{d}W_{\mathrm{mM-1}}/\mathrm{d}\mu') \times (\mathrm{d}\mu'/\mathrm{d}G') \times (\mathrm{d}G'/\mathrm{d}g_{\mathrm{mM-1}})) + c_{\mathrm{mP-1}}((\mathrm{d}W_{\mathrm{mP-1}}/\mathrm{d}\mu) \times (\mathrm{d}\mu/\mathrm{d}G) \times (\mathrm{d}G/\mathrm{d}g_{\mathrm{mP-1}}) + (\mathrm{d}W_{\mathrm{mP-1}}/\mathrm{d}\mu') \times (\mathrm{d}\mu'/\mathrm{d}G') \times (\mathrm{d}G'/\mathrm{d}g_{\mathrm{mP-1}})) > 0$, where: $\mathrm{d}\mu/\mathrm{d}G = \mathrm{d}\mu'/\mathrm{d}G' = 1$; $\mathrm{d}G/\mathrm{d}g_{\mathrm{sTUv}} = q_{\mathrm{sTUv}}$ is the consanguinity between the focal class-sTUv gene and the genes, residing at the same locus, who control PGE in the father of the focal gene's carrier; and $\mathrm{d}G'/\mathrm{d}g_{\mathrm{mTUv}} = q'_{\mathrm{mTUv}}$ is the consanguinity between the focal class-sTUv gene and the genes, residing at the same locus, who control PGE in the males who contribute offspring to the focal individual's mating group.

Consanguinity – Again, PGE in males does not impact upon the transmission of X-linked genes, because males can only transmit maternal-origin X-linked genes anway. Accordingly, the coefficients of consanguinity are exactly the same as those given in Table A1.2.2.

Equilibrium level of PGE – If the condition for increase in PGE is not satisfied at $\bar{\mu} = 0$, then PGE cannot invade the population from rarity, and its equilibrium level is $g^* = 0$. If the condition for increase in PGE is satisfied at $\bar{\mu} = 1$, then non-PGE cannot invade from rarity, and hence the equilibrium level of PGE is $g^* = 1$. Setting the LHS of the condition equal to zero, and solving for $\bar{\mu} = g^*$, obtains the intermediate

equilibrium level of PGE. The equilibrium level of PGE is illustrated in Figure 4 of the main text.

5.3. Y-linked genes

Classes – There are 2 classes of individual, classified according to their sex $s \in \{f,m\}$. If the individual is female, then she has no Y-linked genes. And if the individual is male, then all of his Y-linked genes are paternal in origin (and came from his paternal grandfather). Thus, class is notated in the form $s \in \{f,m\}$.

Survival – The probabilities of survival for each class are $S_f(\mu) = (1/2)(1-\mu) + \mu$ and $S_m(\mu) = (1/2)(1-\mu)$.

Reproductive success – Contingent upon survival, expected reproductive success is $R_f(\mu') = 1$ for females and $R_m(\mu') = (1-z(\mu'))/z(\mu')$ for males, where $z(\mu') = S_m(\mu')/(S_f(\mu') + S_m(\mu'))$.

Fitness – Expected fitness is given by the product of survival to maturity and expected reproductive success contingent upon surviving to maturity, i.e. $w_s(\mu,\mu') = S_s(\mu)\times R_s(\mu')$. The average fitness among all individuals of a particular class is given by $\bar{w}_s(\bar{\mu}) = w_s(\bar{\mu}, \bar{\mu})$, and each individual's fitness can be expressed relative to the average of their class, by $W_s(\mu,\mu', \bar{\mu}) = w_s(\mu,\mu', \bar{\mu})/ \bar{w}_s(\bar{\mu}, \bar{\mu})$.

Gene fitness – There is only one class of Y-linked gene, because females carry no Y-linked genes and males carry one class of Y-linked genes. Accordingly, the relative fitness of a Y-linked gene in a male is simply $W_m(\mu,\mu', \bar{\mu})$.

Reproductive value – Since there is only one class of Y-linked gene, all reproductive value belongs to this class: $c_m = 1$.

PGE in males – As before, the condition for increase in male PGE is c_m $((dW_m/d\mu) \times (d\mu/dG) \times (dG/dg_m) + (dW_m/d\mu') \times (d\mu'/dG') \times (dG'/dg_m)) > 0$, where: $d\mu/dG = d\mu'/dG' = 1$; $dG/dg_m = q_m$ is the consanguinity between the focal class-sTUv gene and the genes, residing at the same locus, who control PGE in the father of the focal gene's carrier; and $dG'/dg_m = q'_m$ is the consanguinity between the focal class-sTUv gene and the genes, residing at the same locus, who control PGE in the males who contribute offspring to the focal individual's mating group.

Consanguinity – PGE in males does not impact upon consanguinity of Y-linked genes, which are exactly the same as those listed in Table A1.3.2.

Equilibrium level of PGE – If the condition for increase in PGE is not satisfied at $\bar{\mu} = 0$, then PGE cannot invade the population from rarity, and its equilibrium level is $g^* = 0$. If the condition for increase in PGE is satisfied at $\bar{\mu} = 1$, then non-PGE cannot invade from rarity, and hence the equilibrium level of PGE is $g^* = 1$. Setting the LHS

of the condition equal to zero, and solving for $\bar{\mu} = g^*$, obtains the intermediate equilibrium level of PGE. The equilibrium level of PGE is illustrated in Figure 4 of the main text.

6. PGE in females under ZW inheritance: equilibrium analysis

6.1. Autosomal genes

Classes – There are 8 classes of individual, classified according to their sex s ∈ {f,m}, the grandparent of origin of their maternal-origin gene T ∈ {M,P} and the grandparent of origin of their paternal-origin gene U ∈ {M,P}. Each class is notated in the form sTU, i.e. fMM, fMP, fPM, fPP, mMM, mMP, mPM and mPP.

Survival – The zygote survival functions are obtained by substituting $\mu = \alpha = 0$ into those of section 3.1. This yields: $S_{\mathrm{fMM}}(\phi) = (1/8)(1-\phi) + (1/2)\phi$; $S_{\mathrm{fMP}}(\phi) = (1/8)(1-\phi) + (1/2)\phi$; $S_{\mathrm{fPM}}(\phi) = (1/8)(1-\phi)$; $S_{\mathrm{fPP}}(\phi) = (1/8)(1-\phi)$; $S_{\mathrm{mMM}}(\phi) = (1/8)(1-\phi)$; $S_{\mathrm{mMP}}(\phi) = (1/8)(1-\phi)$; $S_{\mathrm{mPM}}(\phi) = (1/8)(1-\phi)$; and $S_{\mathrm{mPP}}(\phi) = (1/8)(1-\phi)$.

Reproductive success – Upon survival to maturity, every female achieves a unit of expected reproductive success, and every male achieves $(1-z(\phi'))/z(\phi')$ units of expected reproductive success, where: $z(\phi') = (\sum_{\mathrm{T}\in\{\mathrm{M,P}\},\mathrm{U}\in\{\mathrm{M,P}\}} S_{\mathrm{mTU}}(\phi')) / (\sum_{\mathrm{s}\in\{\mathrm{f,m}\},\mathrm{T}\in\{\mathrm{M,P}\},\mathrm{U}\in\{\mathrm{M,P}\}} S_{\mathrm{sTU}}(\phi'))$ is the sex ratio (proportion male) in his mating group. Thus, contingent upon survival, expected reproductive success is $R_{\mathrm{fTU}}(\phi') = 1$ for females and $R_{\mathrm{mTU}}(\phi') = (1-z(\phi'))/z(\phi')$ for males.

Fitness – Expected fitness is given by the product of survival to maturity and expected reproductive success contingent upon surviving to maturity, i.e. $w_{\mathrm{sTU}}(\phi,\phi') = S_{\mathrm{sTU}}(\phi)\times R_{\mathrm{sTU}}(\phi')$. The average fitness among all individuals of a particular class is given by $\bar{w}_{\mathrm{sTU}}(\bar{\phi}) = w_{\mathrm{sTU}}(\bar{\phi}, \bar{\phi})$, where $\bar{\phi}$ is the average level of female PGE in the population. Each individual's fitness can be expressed relative to the average of their class, by $W_{\mathrm{sTU}}(\phi,\phi', \bar{\phi}) = w_{\mathrm{sTU}}(\phi,\phi')/ \bar{w}_{\mathrm{sTU}}(\bar{\phi})$.

Gene fitness –There are 16 classes of gene, because every class of individual carries one maternal-origin gene and one paternal-origin gene. Thus, gene classes are denoted in the form sTUv, where v ∈ {1,2} according to whether the gene is of maternal (v = 1) or paternal (v = 2) origin. The fitness of a gene, defined in terms of its probability of being transmitted to a potential (rather than a realised) zygote, is proportional to the fitness of the individual who carries it. Consequently, the relative fitness of a gene is equal to the relative fitness of its carrier: $W_{\mathrm{sTU1}}(\phi,\phi', \bar{\phi}) = W_{\mathrm{sTU2}}(\phi,\phi', \bar{\phi}) = W_{\mathrm{sTU}}(\phi,\phi', \bar{\phi})$.

Reproductive value – The flow of genes between classes determines each class's reproductive value. Each gene's class in any generation may provide some information and some uncertainty about that gene's origin in the previous generation. For example, genes of class fMM1 in the present generation are derived

from genes of class fTU1 in the previous generation, where T = M or P and U = M or P. The effects of PGE in a female are accounted for in the survival of her potential offspring: for example, her potential male offspring are eliminated in the event that she undergoes PGE. So the class fTU1 donating genes to the class fMM1 involves T = M or P with unequal probability, because there is possible bias regarding the source of the latter class's maternal-origin genes, and U = M or P with equal probability, because there is no bias regarding the source of their paternal genes. Specifically, T = M with probability $\bar{w}_{\mathrm{fMU1}}/(\bar{w}_{\mathrm{fMU1}} + \bar{w}_{\mathrm{fPU1}}) = (1+3\bar{\phi})/(2+2\bar{\phi})$, and T = P with probability $\bar{w}_{\mathrm{fPU1}}/(\bar{w}_{\mathrm{fMU1}} + \bar{w}_{\mathrm{fPU1}}) = (1-\bar{\phi})/(2+2\bar{\phi})$. Accordingly, $\wp_{\mathrm{fMM1}\leftarrow\mathrm{fMM1}} = (1+3\bar{\phi})/(4+4\bar{\phi})$, $\wp_{\mathrm{fMM1}\leftarrow\mathrm{fMP1}} = (1+3\bar{\phi})/(4+4\bar{\phi})$, $\wp_{\mathrm{fMM1}\leftarrow\mathrm{fPM1}} = (1-\bar{\phi})/(4+4\bar{\phi})$ and $\wp_{\mathrm{fMM1}\leftarrow\mathrm{fPP1}} = (1-\bar{\phi})/(4+4\bar{\phi})$. Note that, making the substitution $\bar{\phi} = 0$, all of these gene-flow coefficients are equal to ¼, as in section 3.1. Also note that, if the donor class involves genes in males, then there is no bias, because no male is the product of a PGE mother (all PGE events lead to the production of daughters). This defines a system of 16 linear equations, which can be written as $\mathbf{c} = \mathbf{c}\bullet\mathbf{G}$, where $\mathbf{c} = \{c_{\mathrm{fMM1}}, c_{\mathrm{fMM2}}, c_{\mathrm{fMP1}}, \ldots, c_{\mathrm{mPP2}}\}$ is the vector of class reproductive values and $\mathbf{G}$ is the gene-flow matrix. Solving (i.e. finding the left eigenvector of $\mathbf{G}$ corresponding to the eigenvalue 1) obtains $c_{\mathrm{fMU1}} = (1+3\bar{\phi})/(16-8\bar{\phi})$, $c_{\mathrm{fMU2}} = ((1-\bar{\phi})(1+3\bar{\phi}))/(8(2-\bar{\phi})(1+\bar{\phi}))$, $c_{\mathrm{fPU1}} = (1-\bar{\phi})/(8(2-\bar{\phi}))$, $c_{\mathrm{fPU2}} = (1-\bar{\phi})^2/(8(2-\bar{\phi})(1+\bar{\phi}))$ and $c_{\mathrm{mTUv}} = (1-\bar{\phi})/(8(2-\bar{\phi}))$. In the absence of female PGE ($\bar{\phi} = 0$), all gene classes have reproductive value $c_{\mathrm{sTUv}} = 1/16$. In the extreme of full female PGE ($\bar{\phi} = 1$), $c_{\mathrm{fMU1}} = ½$, $c_{\mathrm{fMU2}} = 0$, $c_{\mathrm{fPU1}} = 0$, $c_{\mathrm{fPU2}} = 0$ and $c_{\mathrm{mTUv}} = 0$, i.e. all of the population's reproductive value belongs to maternal grandmaternal genes in females (classes fMM1 and fMP1), and no gene in any male has any reproductive value.

PGE in females – The condition for increase in female PGE is $\sum_{s\in\{f,m\},T\in\{M,P\},U\in\{M,P\},v\in\{1,2\}} c_{\mathrm{sTUv}} ((\mathrm{d}W_{\mathrm{sTUv}}/\mathrm{d}\phi) \times (\mathrm{d}\phi/\mathrm{d}G) \times (\mathrm{d}G/\mathrm{d}g_{\mathrm{sTUv}}) + (\mathrm{d}W_{\mathrm{sTUv}}/\mathrm{d}\phi') \times (\mathrm{d}\phi'/\mathrm{d}G') \times (\mathrm{d}G'/\mathrm{d}g_{\mathrm{sTUv}})) > 0$, where G is the average genic value among those genes at the same locus whose expression controls PGE in the focal individual's mother, and G' is the average genic value among those genes at the same locus whose expression controls PGE in the females contributing offspring to the focal individual's mating group. Here: $\mathrm{d}\phi/\mathrm{d}G = \mathrm{d}\phi'/\mathrm{d}G' = 1$; $\mathrm{d}G/\mathrm{d}g_{\mathrm{sTUv}} = p_{\mathrm{sTUv}}$ is the consanguinity between the focal class-sTUv gene and the genes, residing at the same locus, who control PGE in the mother of the focal gene's carrier; and $\mathrm{d}G'/\mathrm{d}g_{\mathrm{sTUv}} = p'_{\mathrm{sTUv}}$ is the consanguinity between the focal class-sTUv gene and the genes, residing at the same locus, who control PGE in the females who contribute offspring to the focal individual's mating group.

Consanguinity – The coefficient of inbreeding is $\rho = a((1/4)\times((1/2) + (1/2)\times(\lambda\,\rho_{\mathrm{M-}} + (1-\lambda)\rho)) + (1/4)\times(\lambda\,\rho_{\mathrm{M-}} + (1-\lambda)\rho) + (1/4)\times\rho + (1/4)\times\psi)$, where $\lambda = 2\bar{\phi}/(1+\bar{\phi})$ is the probability that a focal adult female derived from a PGE egg. The conditional consanguinities of mating partners are: $\rho_{\mathrm{M-}} = a((1/2)((1/2)+(1/2)(\lambda\,\rho_{\mathrm{M-}} + (1-\lambda)\rho)) + (1/2)(\lambda\,\rho_{\mathrm{M-}} + (1-\lambda)\rho))$, from the perspective of the female's maternal-origin gene; $\rho_{\mathrm{-M}} = a((1/2)((1/2)+(1/2)(\lambda\,\rho_{\mathrm{M-}} + (1-\lambda)\rho)) + (1/2)\rho)$, from the perspective of the male's maternal-origin gene; $\rho_{\mathrm{P-}} = a((1/2)\rho + (1/2)\psi)$, from the perspective of the

female's paternal-origin gene; $\rho_{-P} = a((1/2)(\lambda\ \rho_{M-} + (1\text{-}\lambda)\rho) + (1/2)\psi)$, from the perspective of the male's paternal-origin gene; $\rho_{MM} = a((1/2) + (1/2)(\lambda\ \rho_{M-} + (1\text{-}\lambda)\rho))$, from the perspective of both mating partners' maternal-origin genes; $\rho_{MP} = a(\lambda\rho_{M-} + (1\text{-}\lambda)\rho)$, from the perspective of the female's maternal-origin gene and the male's paternal-origin gene; $\rho_{PM} = a\rho$, from the perspective of the female's paternal-origin gene and the male's maternal-origin gene; and $\rho_{PP} = a\ \psi$, from the perspective of both mating partners' paternal-origin genes. The consanguinity of two males in the same mating group is $\psi = a((1/4)((1/2) + (1/2)\times(\lambda\ \rho_{M-} + (1\text{-}\lambda)\rho)) + (1/2)\rho + (1/4)\psi)$, and the conditional consanguinities between males are: $\psi_{M-} = a((1/2)((1/2) + (1/2)\times(\lambda\ \rho_{M-} + (1\text{-}\lambda)\rho)) + (1/2)\rho)$, from the perspective of one male's maternal-origin gene; $\psi_{P-} = a((1/2)\rho + (1/2)\psi)$, from the perspective of one male's paternal-origin gene; $\psi_{MM} = a((1/2) + (1/2)(\lambda\ \rho_{M-} + (1\text{-}\lambda)\rho))$, from the perspective of both males' maternal-origin genes; $\psi_{MP} = a\rho$, from the perspective of one male's maternal-origin gene and the other's paternal-origin gene; and $\psi_{PP} = a\psi$, from the perspective of both males' paternal-origin genes. The consanguinity of two females in the same mating group is $\zeta = a((1/4)(\lambda^2 + (1\text{-}\lambda^2)((1/2) + (1/2)(\lambda\ \rho_{M-} + (1\text{-}\lambda)\rho)) + (1/2)(\lambda\ \rho_{M-} + (1\text{-}\lambda)\rho)\ + (1/4)\psi)$, and the conditional consanguinities between females are: $\zeta_{M-} = a((1/2)(\lambda^2 + (1\text{-}\lambda^2)((1/2) + (1/2)(\lambda\ \rho_{M-} + (1\text{-}\lambda)\rho)) + (1/2)(\lambda\ \rho_{M-} + (1\text{-}\lambda)\rho))$, from the perspective of one female's maternal-origin gene; $\zeta_{P-} = a((1/2)(\lambda\ \rho_{M-} + (1\text{-}\lambda)\rho)\ + (1/2)\psi)$, from the perspective of one female's paternal-origin gene; $\zeta_{MM} = a((\lambda^2 + (1\text{-}\lambda^2)((1/2) + (1/2)(\lambda\ \rho_{M-} + (1\text{-}\lambda)\rho)))$, from the perspective of both females' maternal-origin genes; $\zeta_{MP} = a(\lambda\ \rho_{M-} + (1\text{-}\lambda)\rho)$, from the perspective of one female's maternal-origin gene and the other's paternal-origin gene; and $\zeta_{PP} = a\ \psi$, from the perspective of both females' paternal-origin genes. Simultaneously solving these equations yields $\rho = a(4+(4\text{-}a^2)\ \bar{\phi})/(32(1+\ \bar{\phi})\ \text{-}\ a(28+(2\text{-}a(26+3a))\ \bar{\phi}))$, and each of the other consanguinities. And these in turn define all the consanguinities needed to solve the model (listed in Table A6.1.1).

Equilibrium level of PGE – If the condition for increase in PGE is not satisfied at $\bar{\phi} = 0$, then PGE cannot invade the population from rarity, and its equilibrium level is $g^* = 0$. If the condition for increase in PGE is satisfied at $\bar{\phi} = 1$, then non-PGE cannot invade from rarity, and hence the equilibrium level of PGE is $g^* = 1$. Setting the LHS of the condition equal to zero, and solving for $\bar{\phi} = g^*$, obtains the intermediate equilibrium level of PGE. The equilibrium level of PGE is illustrated in Figure 4 of the main text.

6.2. Z-linked genes

Classes – There are 4 classes of individual, classified according to their sex $s \in \{f,m\}$ and the grandparent of origin of their paternal-origin gene $U \in \{M,P\}$. If the individual is female, she has no maternal-origin Z-linked genes. And if the individual is male, then all of his maternal-origin Z-linked genes are derived from his maternal grandfather. Thus, each class is notated in the form sTU, where $T \in \{M,\text{-}\}$, i.e. f-M, f-P, mPM and mPP.

Recipient Class	Actor Class A	A_{Mat}	A_{Pat}	A_{Mot}	A_{Fat}
fMM1	$(1+\nu)/2$	1	ν	$(1+\nu)/2$	ν
fMM2	$\rho_{\text{-M}}$	ρ_{MM}	ρ_{PM}	$a(1+\nu)/2$	$a\,\rho$
fMP1	$(1+\nu)/2$	1	ν	$(1+\nu)/2$	ν
fMP2	$\rho_{\text{-P}}$	ρ_{MP}	ρ_{PP}	$a\,\rho$	$a\,\psi$
fPM1	$(1+\nu)/2$	ν	1	ρ	$(1+\rho)/2$
fPM2	$\rho_{\text{-M}}$	ρ_{MM}	ρ_{PM}	$a(1+\nu)/2$	$a\,\rho$
fPP1	$(1+\nu)/2$	ν	1	ρ	$(1+\rho)/2$
fPP2	$\rho_{\text{-P}}$	ρ_{MP}	ρ_{PP}	$a\,\rho$	$a\,\psi$
mMM1	$(1+\nu)/2$	1	ν	$(1+\nu)/2$	ν
mMM2	$\rho_{\text{-M}}$	ρ_{MM}	ρ_{PM}	$a(1+\nu)/2$	$a\,\rho$
mMP1	$(1+\nu)/2$	1	ν	$(1+\nu)/2$	ν
mMP2	$\rho_{\text{-P}}$	ρ_{MP}	ρ_{PP}	$a\,\rho$	$a\,\psi$
mPM1	$(1+\nu)/2$	ν	1	ρ	$(1+\rho)/2$
mPM2	$\rho_{\text{-M}}$	ρ_{MM}	ρ_{PM}	$a(1+\nu)/2$	$a\,\rho$
mPP1	$(1+\nu)/2$	ν	1	ρ	$(1+\rho)/2$
mPP2	$\rho_{\text{-P}}$	ρ_{MP}	ρ_{PP}	$a\,\rho$	$a\,\psi$
mMM1′	$a(1+\nu)/2$	a	$a\,\nu$	$a(1+\nu)/2$	$a\,\nu$
mMM2′	$a\rho_{\text{-M}}$	$a\,\rho_{MM}$	$a\,\rho_{PM}$	$a^2(1+\nu)/2$	$a^2\,\rho$
mMP1′	$a(1+\nu)/2$	a	$a\,\nu$	$a(1+\nu)/2$	$a\,\nu$
mMP2′	$a\rho_{\text{-P}}$	$a\,\rho_{MP}$	$a\,\rho_{PP}$	$a^2\rho$	$a^2\,\psi$
mPM1′	$a(1+\nu)/2$	$a\,\nu$	a	$a\,\rho$	$a(1+\rho)/2$
mPM2′	$a\rho_{\text{-M}}$	$a\,\rho_{MM}$	$a\,\rho_{PM}$	$a^2(1+\nu)/2$	$a^2\,\rho$
mPP1′	$a(1+\nu)/2$	$a\,\nu$	a	$a\,\rho$	$a(1+\rho)/2$
mPP2′	$a\rho_{\text{-P}}$	$a\,\rho_{MP}$	$a\,\rho_{PP}$	$a^2\,\rho$	$a^2\,\psi$

Table A6.1.1. Consanguinities for the female PGE equilibrium analysis: autosomal genes. The five autosomal actor classes are the female's autosomal genes (A), her maternal-origin autosomal genes (A_{Mat}), her paternal-origin autosomal genes (A_{Pat}), her mother's autosomal genes (A_{Mot}) and her father's autosomal genes (A_{Fat}). Shown here are their consanguinities p_{sTUv} to each of the recipient classes sTUv among the focal female's offspring, and the consanguinities p'_{mTUv} to each of the recipient classes mTUv′, among the males competing for mates with the focal female's sons. Here, $\nu = (\lambda\,\rho_{M\text{-}} + (1\text{-}\lambda)\rho)$.

Survival – The probabilities of survival for each class are: $S_{f\text{-}M}(\phi) = (1/4)(1\text{-}\phi) + (1/2)\phi$; $S_{f\text{-}P}(\phi) = (1/4)(1\text{-}\phi) + (1/2)\phi$; $S_{mPM}(\phi) = (1/4)(1\text{-}\phi)$; and $S_{mPP}(\phi) = (1/4)(1\text{-}\phi)$.

Reproductive success – Contingent upon survival, expected reproductive success is $R_{fTU}(\phi') = 1$ for females and $R_{mTU}(\phi') = (1\text{-}z(\phi'))/z(\phi')$ for males, where $z(\phi') = (S_{mPM}(\phi') + S_{mPP}(\phi'))/(S_{f\text{-}M}(\phi') + S_{f\text{-}P}(\phi') + S_{mPM}(\phi') + S_{mPP}(\phi'))$.

Fitness – Expected fitness is given by the product of survival to maturity and expected reproductive success contingent upon surviving to maturity, i.e. $w_{sTU}(\phi,\phi')$

= $S_{\mathrm{sTU}}(\phi)\times R_{\mathrm{sTU}}(\phi')$. The average fitness among all individuals of a particular class is given by $\bar{w}_{\mathrm{sTU}}(\bar{\phi}) = w_{\mathrm{sTU}}(\bar{\phi}, \bar{\phi})$. Each individual's fitness can be expressed relative to the average of their class, by $W_{\mathrm{sTU}}(\phi,\phi', \bar{\phi}) = w_{\mathrm{sTU}}(\phi,\phi')/\ \bar{w}_{\mathrm{sTU}}(\bar{\phi})$.

Gene fitness –There are 6 classes of gene, because individuals of each of the two female classes carry only paternal-origin genes, whereas individuals of each of the two male classes carry separate maternal-origin and paternal-origin genes. As before, gene classes are denoted in the form sTUv, where v ∈ {1,2} according to whether the gene is of maternal (v = 1) or paternal (v = 2) origin. The relative fitness of a gene in a female is given by $W_{\mathrm{f\text{-}U2}}(\phi,\phi', \bar{\phi}) = W_{\mathrm{f\text{-}U}}(\phi,\phi', \bar{\phi})$ and the relative fitness of a gene in a male is given by $W_{\mathrm{mPU1}}(\phi,\phi', \bar{\phi}) = W_{\mathrm{mPU2}}(\phi,\phi', \bar{\phi}) = W_{\mathrm{mPU}}(\phi,\phi', \bar{\phi})$.

Reproductive value – PGE in females does not impact upon the gene-flow coefficients for Z-linked genes, because females can only transmit paternal-origin Z-linked genes anway. Accordingly, all of the non-zero gene-flow coefficients are equal to ½, and reproductive value is $c_{\mathrm{sTUv}} = 1/6$ for all gene classes, as in section A3.2.

PGE in females – Following the same procedure as before, the condition for increase in female PGE is $c_{\mathrm{f\text{-}M2}}$ $((\mathrm{d}W_{\mathrm{f\text{-}M2}}/\mathrm{d}\phi) \times (\mathrm{d}\phi/\mathrm{d}G) \times (\mathrm{d}G/\mathrm{d}g_{\mathrm{f\text{-}M2}})) + c_{\mathrm{f\text{-}P2}}$ $((\mathrm{d}W_{\mathrm{f\text{-}P2}}/\mathrm{d}\phi) \times (\mathrm{d}\phi/\mathrm{d}G) \times (\mathrm{d}G/\mathrm{d}g_{\mathrm{f\text{-}P2}})) + c_{\mathrm{mPM1}}$ $((\mathrm{d}W_{\mathrm{mPM1}}/\mathrm{d}\phi) \times (\mathrm{d}\phi/\mathrm{d}G) \times (\mathrm{d}G/\mathrm{d}g_{\mathrm{mPM1}}) + (\mathrm{d}W_{\mathrm{mPM1}}/\mathrm{d}\phi') \times (\mathrm{d}\phi'/\mathrm{d}G') \times (\mathrm{d}G'/\mathrm{d}g_{\mathrm{mPM1}})) + c_{\mathrm{mPM2}}$ $((\mathrm{d}W_{\mathrm{mPM2}}/\mathrm{d}\phi) \times (\mathrm{d}\phi/\mathrm{d}G) \times (\mathrm{d}G/\mathrm{d}g_{\mathrm{mPM2}}) + (\mathrm{d}W_{\mathrm{mPM2}}/\mathrm{d}\phi') \times (\mathrm{d}\phi'/\mathrm{d}G') \times (\mathrm{d}G'/\mathrm{d}g_{\mathrm{mPM2}})) + c_{\mathrm{mPP1}}$ $((\mathrm{d}W_{\mathrm{mPP1}}/\mathrm{d}\phi) \times (\mathrm{d}\phi/\mathrm{d}G) \times (\mathrm{d}G/\mathrm{d}g_{\mathrm{mPP1}}) + (\mathrm{d}W_{\mathrm{mPP1}}/\mathrm{d}\phi') \times (\mathrm{d}\phi'/\mathrm{d}G') \times (\mathrm{d}G'/\mathrm{d}g_{\mathrm{mPP1}})) + c_{\mathrm{mPP2}}$ $((\mathrm{d}W_{\mathrm{mPP2}}/\mathrm{d}\phi) \times (\mathrm{d}\phi/\mathrm{d}G) \times (\mathrm{d}G/\mathrm{d}g_{\mathrm{mPP2}}) + (\mathrm{d}W_{\mathrm{mPP2}}/\mathrm{d}\phi') \times (\mathrm{d}\phi'/\mathrm{d}G') \times (\mathrm{d}G'/\mathrm{d}g_{\mathrm{mPP2}})) > 0$, where: $\mathrm{d}\phi/\mathrm{d}G = \mathrm{d}\phi'/\mathrm{d}G' = 1$; $\mathrm{d}G/\mathrm{d}g_{\mathrm{sTUv}} = p_{\mathrm{sTUv}}$ is the consanguinity between the focal class-sTUv gene and the genes, residing at the same locus, who control PGE in the mother of the focal gene's carrier; and $\mathrm{d}G'/\mathrm{d}g_{\mathrm{mTUv}} = p'_{\mathrm{mTUv}}$ is the consanguinity between the focal class-sTUv gene and the genes, residing at the same locus, who control PGE in the females who contribute offspring to the focal individual's mating group.

Consanguinity – Again, PGE in females does not impact upon the transmission of Z-linked genes, because females can only transmit maternal-origin Z-linked genes anway. Accordingly, the coefficients of consanguinity are exactly the same as those given in Table A3.2.1.

Equilibrium level of PGE – If the condition for increase in PGE is not satisfied at $\bar{\phi} = 0$, then PGE cannot invade the population from rarity, and its equilibrium level is $g^* = 0$. If the condition for increase in PGE is satisfied at $\bar{\phi} = 1$, then non-PGE cannot invade from rarity, and hence the equilibrium level of PGE is $g^* = 1$. Setting the LHS of the condition equal to zero, and solving for $\bar{\phi} = g^*$, obtains the intermediate equilibrium level of PGE. The equilibrium level of PGE is illustrated in Figure 4 of the main text.

6.3. W-linked genes

Classes – There are 2 classes of individual, classified according to their sex s $\in$ {f,m}. If the individual is female, then all of her W-linked genes are maternal in origin (and came from her maternal grandmother). And if the individual is male, he has no W-linked genes. Thus, class is notated in the form s $\in$ {f,m}.

Survival – The probabilities of survival for each class are $S_f(\phi) = (1/2)(1-\phi) + \phi$ and $S_m(\phi) = (1/2)(1-\phi)$.

Reproductive success – Contingent upon survival, expected reproductive success is $R_f(\phi') = 1$ for females and $R_m(\phi') = (1-z(\phi'))/z(\phi')$ for males, where $z(\phi') = S_m(\phi')/(S_f(\phi') + S_m(\phi'))$.

Fitness – Expected fitness is given by the product of survival to maturity and expected reproductive success contingent upon surviving to maturity, i.e. $w_{sTU}(\phi,\phi') = S_{sTU}(\phi)\times R_{sTU}(\phi')$. The average fitness among all individuals of a particular class is given by $\bar{w}_{sTU}(\bar{\phi}) = w_{sTU}(\bar{\phi}, \bar{\phi})$. Each individual's fitness can be expressed relative to the average of their class, by $W_{sTU}(\phi,\phi', \bar{\phi}) = w_{sTU}(\phi,\phi')/ \bar{w}_{sTU}(\bar{\phi})$.

Gene fitness – There is only one class of W-linked gene, because females carry only one class of W-linked gene and males carry no W-linked genes. Accordingly, the relative fitness of a W-linked gene in a female is simply $W_f(\phi,\phi', \bar{\phi})$.

Reproductive value – Since there is only one class of W-linked gene, all reproductive value belongs to this class: $c_f = 1$.

PGE in females – Following the same procedure as before, the condition for increase in female PGE is $c_f\ ((dW_f/d\phi) \times (d\phi/dG) \times (dG/dg_f)) > 0$. Once again: $d\phi/dG$ is an arbitrary mapping between genic value and phenotypic value, and can be set to 1; and $dG/dg_f = p_f$ is the consanguinity between the focal gene and the genes, residing at the same locus, who control PGE in the mother of the focal gene's carrier.

Consanguinity – PGE in females does not impact upon consanguinity of W-linked genes, which are exactly the same as those listed in Table A3.3.1.

Equilibrium level of PGE – If the condition for increase in PGE is not satisfied at $\bar{\phi} = 0$, then PGE cannot invade the population from rarity, and its equilibrium level is $g^* = 0$. If the condition for increase in PGE is satisfied at $\bar{\phi} = 1$, then non-PGE cannot invade from rarity, and hence the equilibrium level of PGE is $g^* = 1$. Setting the LHS of the condition equal to zero, and solving for $\bar{\phi} = g^*$, obtains the intermediate equilibrium level of PGE. The equilibrium level of PGE is illustrated in Figure 4 of the main text.

7. PGE in males under XY/XO inheritance: equilibrium analysis with a new mode of sex determination

7.1. Autosomal genes

Classes – There are 8 classes of individual, classified according to their sex s ∈ {f,m}, the grandparent of origin of their maternal-origin gene T ∈ {M,P} and the grandparent of origin of their paternal-origin gene U ∈ {M,P}. Each class is notated in the form sTU, i.e. fMM, fMP, fPM, fPP, mMM, mMP, mPM and mPP.

Survival – If sex allocation z is determined independently of genotype, then the zygote survival functions are $S_{\text{fMM}}(\mu,z) = (1\text{-}z)((1/4)(1\text{-}\mu) + (1/2)\mu)$; $S_{\text{fMP}}(\mu,z) = (1\text{-}z)(1/4)(1\text{-}\mu)$; $S_{\text{fPM}}(\mu,z) = (1\text{-}z)((1/4)(1\text{-}\mu) + (1/2)\mu)$; $S_{\text{fPP}}(\mu,z) = (1\text{-}z)(1/4)(1\text{-}\mu)$; $S_{\text{mMM}}(\mu,z) = z((1/4)(1\text{-}\mu)+(1/2)\mu)$; $S_{\text{mMP}}(\mu,z) = z(1/4)(1\text{-}\mu)$; $S_{\text{mPM}}(\mu,z) = z((1/4)(1\text{-}\mu)+(1/2)\mu)$; and $S_{\text{mPP}}(\mu,z) = z(1/4)(1\text{-}\mu)$.

Reproductive success – Upon survival to maturity, every female achieves a unit of expected reproductive success, and every male achieves $(1\text{-}z')/ z'$ units of expected reproductive success, where z' is the sex ratio of the group in which they compete for mates. Thus, contingent upon survival, expected reproductive success is $R_{\text{fTU}}(z') = 1$ for females and $R_{\text{mTU}}(z') = (1\text{-} z')/z'$ for males.

Fitness – Expected fitness is given by the product of survival to maturity and expected reproductive success contingent upon surviving to maturity, i.e. $w_{\text{sTU}}(\mu,z,z') = S_{\text{sTU}}(\mu,z)\times R_{\text{sTU}}(z')$. The average fitness among all individuals of a particular class is given by $\bar{w}_{\text{sTU}}(\bar{\mu}, \bar{z}) = w_{\text{sTU}}(\bar{\mu}, \bar{z}, \bar{z})$, and each individual's fitness can be expressed relative to the average of their class, by $W_{\text{sTU}}(\mu,z,z', \bar{z}) = w_{\text{sTU}}(\mu,z,z')/ \bar{w}_{\text{sTU}}(\bar{\mu}, \bar{z})$.

Gene fitness –There are 16 classes of gene, because every class of individual carries one maternal-origin gene and one paternal-origin gene. Thus, gene classes are denoted in the form sTUv, where v ∈ {1,2} according to whether the gene is of maternal (v = 1) or paternal (v = 2) origin. The fitness of a gene, defined in terms of its probability of being transmitted to a potential (rather than a realised) zygote, is proportional to the fitness of the individual who carries it. Consequently, the relative fitness of a gene is equal to the relative fitness of its carrier: $W_{\text{sTU1}}(\mu,z,z', \bar{z}) = W_{\text{sTU2}}(\mu,z,z', \bar{z}) = W_{\text{sTU}}(\mu,z,z', \bar{z})$.

Reproductive value – The flow of genes between classes determines each class's reproductive value. Each gene's class in any generation may provide some information and some uncertainty about that gene's origin in the previous generation. For example, genes of class fMM1 in the present generation are derived from genes of class fTU1 in the previous generation, where T = M or P and U = M or P. The effects of PGE in a male are accounted for in the survival of his potential offspring: for example, his potential male offspring are eliminated in the event that

he undergoes PGE. So the class fTU1 donating genes to the class fMM1 involves T = M or P with equal probability, because there is no bias regarding the source of their maternal genes, but U = M or P with unequal probability, because there is possible bias regarding the source of their paternal genes. Specifically, U = M with probability $\bar{w}_{fTM1}/(\bar{w}_{fTM1} + \bar{w}_{fTP1}) = (1+\bar{\mu})/2$, and U = P with probability $\bar{w}_{fTP1}/(\bar{w}_{fTM1} + -\bar{w}_{fTP1}) = (1-\bar{\mu})/2$. Accordingly, $\wp_{fMM1\leftarrow fMM1} = (1+\bar{\mu})/4$, $\wp_{fMM1\leftarrow fPM1} = (1+\bar{\mu})/4$, $\wp_{fMM1\leftarrow fMP1} = (1-\bar{\mu})/4$ and $\wp_{fMM1\leftarrow fPP1} = (1-\bar{\mu})/4$. Note that, making the substitution $\bar{\mu} = 0$, all of these gene-flow coefficients are equal to ¼, as in section 1.1. This defines a system of 16 linear equations, which can be written as $\mathbf{c} = \mathbf{c}\bullet\mathbf{G}$, where $\mathbf{c} = \{c_{fMM1}, c_{fMM2}, c_{fMP1}, \ldots, c_{mPP2}\}$ is the vector of class reproductive values and **G** is the gene-flow matrix. Solving (i.e. finding the left eigenvector of **G** corresponding to the eigenvalue 1) obtains $c_{fTMv} = (1+\bar{\mu})^2/(16+8\bar{\mu})$, $c_{fTPv} = (1-\bar{\mu}^2)/(16+8\bar{\mu})$, $c_{mTM1} = (1+\bar{\mu})^2/(16+8\bar{\mu})$, $c_{mTM2} = (1-\bar{\mu}^2)/(16+8\bar{\mu})$, $c_{mTP1} = (1-\bar{\mu}^2)/(16+8\bar{\mu})$, and $c_{mTP2} = (1-\bar{\mu})^2/(16+8\bar{\mu})$.

PGE in males – The condition for increase in male PGE is $\sum_{s\in\{f,m\},T\in\{M,P\},U\in\{M,P\},v\in\{1,2\}} c_{sTUv} (dW_{sTUv}/d\mu) \times (d\mu/dG) \times (dG/dg_{sTUv}) > 0$, where G is the average genic value among those genes at the same locus whose expression controls PGE in the focal individual's father. Here: $d\mu/dG = 1$ and $dG/dg_{sTUv} = q_{sTUv}$ is the consanguinity between the focal class-sTUv gene and the genes, residing at the same locus, who control PGE in the father of the focal gene's carrier. The consanguinities will depend upon which class or classes of genes control PGE in males -- see the Consanguinity section below, and Table A7.1.1, for details.

Consanguinity – Note that there are no sex differences in genes carried (but there are sex differences in genes transmitted), so $\psi = \zeta = \rho$. The coefficient of inbreeding is $\rho = a((1/4)\times((1/2) + (1/2)\times(\bar{\mu}\rho_{-M} + (1-\bar{\mu})\rho)) + (1/2)\times(\bar{\mu}\rho_{-M} + (1-\bar{\mu})\rho) + (1/4)\times(\bar{\mu}^2\rho_{MM} + 2\bar{\mu}(1-\bar{\mu})\rho_{M-} + (1-\bar{\mu})^2\rho))$. Note that, whilst the consanguinity of mating partners is ρ, the consanguinity of the genes that they transmit to their offspring is $\bar{\mu}\rho_{-M} + (1-\bar{\mu})\rho$, because with probability $\bar{\mu}$ the male exhibits PGE and so only transmits his maternal-origin genes, and with probability $1-\bar{\mu}$ the male does not exhibit PGE and hence transmits maternal-origin and paternal-origin genes with equal probability. The conditional consanguinities of mating partners are: $\rho_{-M} = \rho_{M-} = a((1/2)\times((1/2) + (1/2)\times(\bar{\mu}\rho_{-M} + (1-\bar{\mu})\rho)) + (1/2)\times(\bar{\mu}\rho_{-M} + (1-\bar{\mu})\rho))$, from the perspective of one partner's maternal-origin gene; $\rho_{-P} = \rho_{P-} = a((1/2)\times(\bar{\mu}\rho_{-M} + (1-\bar{\mu})\rho) + (1/2)\times(\bar{\mu}^2\rho_{MM} + 2\bar{\mu}(1-\bar{\mu})\rho_{M-} + (1-\bar{\mu})^2\rho))$, from the perspective of one partner's paternal-origin gene; $\rho_{MM} = a((1/2) + (1/2)\times(\bar{\mu}\rho_{-M} + (1-\bar{\mu})\rho))$, from the perspective of both mating partners' maternal-origin genes; $\rho_{MP} = \rho_{PM} = a(\bar{\mu}\rho_{-M} + (1-\bar{\mu})\rho)$, from the perspective of one partner's maternal-origin gene and the other partner's paternal-origin gene; and $\rho_{PP} = a(\bar{\mu}^2\rho_{MM} + 2\bar{\mu}(1-\bar{\mu})\rho_{M-} + (1-\bar{\mu})^2\rho)$, from the perspective of both mating partners' paternal-origin genes. Simultaneously solving these equations yields $\rho = a(2+a(3-a\bar{\mu}^2)\bar{\mu})/(16-a(14-(6-a(3-\bar{\mu})(1-\bar{\mu})-4\bar{\mu})\bar{\mu}))$, and each of the other consanguinities. And these in turn define all the consanguinities needed to solve the model (listed in Table A7.1.1).

	Actor Class				
**Recipient Class**	A	A_{Mat}	A_{Pat}	A_{Mot}	A_{Fat}
fMM1	ρ_{M-}	ρ_{MM}	ρ_{MP}	$a\,(1+\sigma)/2$	$a\,\rho$
fMM2	$(1+\sigma)/2$	1	σ	$(1+\sigma)/2$	ρ
fMP1	ρ_{M-}	ρ_{MM}	ρ_{MP}	$a(1+\sigma)/2$	$a\,\rho$
fMP2	$(1+\sigma)/2$	σ	1	σ	$(1+\sigma)/2$
fPM1	ρ_{P-}	ρ_{PM}	ρ_{PP}	$a\,\sigma$	$a\,\sigma$
fPM2	$(1+\sigma)/2$	1	σ	$(1+\sigma)/2$	ρ
fPP1	ρ_{P-}	ρ_{PM}	ρ_{PP}	$a\,\sigma$	$a\,\sigma$
fPP2	$(1+\sigma)/2$	σ	1	σ	$(1+\sigma)/2$
mMM1	ρ_{M-}	ρ_{MM}	ρ_{MP}	$a(1+\sigma)/2$	$a\,\rho$
mMM2	$(1+\sigma)/2$	1	σ	$(1+\sigma)/2$	ρ
mMP1	ρ_{M-}	ρ_{MM}	ρ_{MP}	$a(1+\sigma)/2$	$a\,\rho$
mMP2	$(1+\sigma)/2$	σ	1	σ	$(1+\sigma)/2$
mPM1	ρ_{P-}	ρ_{PM}	ρ_{PP}	$a\,\sigma$	$a\,\sigma$
mPM2	$(1+\sigma)/2$	1	σ	$(1+\sigma)/2$	ρ
mPP1	ρ_{P-}	ρ_{PM}	ρ_{PP}	$a\,\sigma$	$a\,\sigma$
mPP2	$(1+\sigma)/2$	σ	1	σ	$(1+\sigma)/2$

Table A7.1.1. Consanguinities for the male PGE equilibrium analysis: autosomal genes. The five autosomal actor classes are the male's autosomal genes (A), his maternal-origin autosomal genes (A_{Mat}), his paternal-origin autosomal genes (A_{Pat}), his mother's autosomal genes (A_{Mot}) and his father's autosomal genes (A_{Fat}). Shown here are their consanguinities q_{sTUv} to each of the recipient classes sTUv among the male's offspring, and the consanguinities q'_{mTUv} to each of the recipient classes mTUv′, among the males competing for mates with the focal male's sons. Here, $\sigma = (\bar{\mu}\,\rho_{-M} + (1-\bar{\mu})\rho)$.

Equilibrium level of PGE – If the condition for increase in PGE is not satisfied at $\bar{\mu} = 0$, then PGE cannot invade the population from rarity, and its equilibrium level is $g^* = 0$. If the condition for increase in PGE is satisfied at $\bar{\mu} = 1$, then non-PGE cannot invade from rarity, and hence the equilibrium level of PGE is $g^* = 1$. Setting the LHS of the condition equal to zero, and solving for $\bar{\mu} = g^*$, obtains the intermediate equilibrium level of PGE.

8. Combined action of multiple genic actors with conflicting interests

8.1. Selection under conflict

Total selective change – As outlined in the above sections, a gene that promotes GE is favoured by natural selection if $\mathrm{d}W/\mathrm{d}g > 0$ and disfavoured by natural selection if $\mathrm{d}W/\mathrm{d}g < 0$. The corresponding selective change in the frequency of GE is $(\mathrm{d}W/\mathrm{d}g) \times \mathrm{var}(g)$, where $\mathrm{var}(g)$ is the additive genetic variance in GE contributed by that gene position. If several genes of the same type (e.g. unimprinted autosomal genes) impact upon the GE phenotype, then the corresponding selective change in the frequency of GE is $(\mathrm{d}W/\mathrm{d}g) \times \mathrm{var}(g)$, where $\mathrm{var}(g)$ is the total additive genetic

variance in GE contributed by those gene positions. And if several genes of different types (e.g. unimprinted autosomal genes and imprinted maternal-origin genes and paternal-origin genes) impact upon the GE phenotype, then the corresponding selective change in the frequency of GE is $\sum_{t\in T}(\mathrm{d}W/\mathrm{d}g)_t\, \pi_t\, \mathrm{var}(g)$, where T is the set of all types of gene position impacting upon the GE phenotype, $\mathrm{var}(g)$ is the total additive genetic variance in GE contributed by those gene positions, $(\mathrm{d}W/\mathrm{d}g)_t$ is the marginal fitness associated with gene positions of type t, and π_t is the proportion of the total additive genetic variance that is contributed by gene positions of type t.

Condition for increase – The condition for natural selection to favour an increase in the frequency of GE is $\sum_{t\in T}(\mathrm{d}W/\mathrm{d}g)_t\, \pi_t > 0$, where the LHS of the condition represents a weighted average of the interests of the different genic types.

Equilibrium point – Note that $(\mathrm{d}W/\mathrm{d}g)_{t^\circ} \leq \sum_{t\in T}(\mathrm{d}W/\mathrm{d}g)_t\, \pi_t \leq (\mathrm{d}W/\mathrm{d}g)_{t^*}$, where $t^\circ \in T$ is the type of gene position least-strongly favouring (or most-strongly disfavouring) GE – i.e. $(\mathrm{d}W/\mathrm{d}g)_{t^\circ} \leq (\mathrm{d}W/\mathrm{d}g)_t$ for all $t \in T$ – and $t^* \in T$ is the type of gene position most-strongly favouring (or least-strongly disfavouring) GE – i.e. $(\mathrm{d}W/\mathrm{d}g)_{t^*} \geq (\mathrm{d}W/\mathrm{d}g)_t$ for all $t \in T$. Hence, $\sum_{t\in T}(\mathrm{d}W/\mathrm{d}g)_t\, \pi_t \geq 0$ when $(\mathrm{d}W/\mathrm{d}g)_{t^\circ} = 0$, and $\sum_{t\in T}(\mathrm{d}W/\mathrm{d}g)_t\, \pi_t \leq 0$ when $(\mathrm{d}W/\mathrm{d}g)_{t^*} = 0$, such that the equilibrium level of GE under conflict is bounded by the equilibria favoured by the extremist parties.

8.2. The parliament of genes

Interests of maternal-origin and paternal-origin genes cancel – The marginal fitness for a maternal-origin gene at a diploid locus may be expressed as $(\mathrm{d}W/\mathrm{d}g)_M = -c + \sum_{i\in I} b_i\, p_{iM}$, i.e. its inclusive-fitness effect, where $-c$ is the impact an increase in gene expression has on its own replicative success, b_i is the impact on the replicative success of its social partner $i \in I$, and p_{iM} is its consanguinity with this social partner. The marginal fitness for the corresponding paternal-origin gene may be expressed as $(\mathrm{d}W/\mathrm{d}g)_P = -c + \sum_{i\in I} b_i\, p_{iP}$, where p_{iP} need not be equal to p_{iM} on account of social partners being more related through one parent than through the other. This asymmetry in consanguinity coefficients, and hence inclusive-fitness effects, is the reason for intragenomic conflict between maternal-origin and paternal-origin genes. If maternal-origin and paternal-origin genes contribute equally to additive genetic variance ($\pi_M = \pi_P = \pi$), then $\sum_{t\in\{M,P\}}(\mathrm{d}W/\mathrm{d}g)_t\, \pi_t > 0$ is equivalent to $-c + \sum_{i\in I} b_i\, p_i > 0$ where $p_i = (p_{iM}+p_{iP})/2$ is the consanguinity that has not been conditioned upon parent of origin, and this inequality is equivalent to $(\mathrm{d}W/\mathrm{d}g)_U > 0$, where U denotes the corresponding genes that lack information as to their parent of origin. For example, considering the evolution of PGE in males under XY/XO sex determination (section 1 above, and SM2 section 1), the marginal fitness for maternal-origin autosomal genes is $(\mathrm{d}W/\mathrm{d}g)_M = (4-8\beta-a(4-5\beta-a(4-2a(1-\beta)-3\beta)))/(2(8-7a))$ and the marginal fitness for paternal-origin autosomal genes is $(\mathrm{d}W/\mathrm{d}g)_P = -(4-a(4-\beta+a(2a(1-\beta)-\beta)))/(2(8-7a))$ and the average of these two exactly recovers the marginal fitness for unimprinted autosomal genes, $(\mathrm{d}W/\mathrm{d}g)_U = (a^2(1-\beta)-2\beta+a\beta)/(8-7a)$. Accordingly, the combined action of multiple maternal-origin and paternal-

origin genes is expected to give an equilibrium level of GE that approximately coincides with that favoured by their unimprinted counterparts.

Majority party dominates the conflict – The weighted-average marginal fitness $\sum_{t \in T}(dW/dg)_t \, \pi_t \rightarrow (dW/dg)_{t'}$ as $\pi_{t'} \rightarrow 1$. That is, the interests of the majority party dominate the conflict. If the individual's own autosomal genes (including unimprinted genes and imprinted genes of maternal-origin and paternal-origin; see above) contribute most of the additive genetic variance in GE, for example on account of their numerical superiority over the sex chromosomes and also because the individual's own genome is necessarily closely involved with the process of GE whereas its parents' genomes can act only at a distance and so have less control, then the majority interest will be equivalent to that of the individual's autosomal genes lacking information as to their parent of origin.
